# Regularity of the Optimal Sets for a Class of Integral Shape Functionals

**DOI:** 10.1007/s00205-024-01984-y

**Published:** 2024-05-15

**Authors:** Giuseppe Buttazzo, Francesco Paolo Maiale, Dario Mazzoleni, Giorgio Tortone, Bozhidar Velichkov

**Affiliations:** 1 Dipartimento di Matematica, Università di Pisa, Largo Bruno Pontecorvo, 5, 56127 Pisa, Italy; 2 Gran Sasso Science Institute, Viale F. Crispi 7, 67100 L’Aquila, Italy; 3 Dipartimento di Matematica, University of Pavia, Via Ferrata 5, 27100 Pavia, Italy

## Abstract

We prove the first regularity theorem for the free boundary of solutions to shape optimization problems involving integral functionals, for which the energy of a domain $\Omega$ is obtained as the integral of a cost function *j*(*u*, *x*) depending on the solution *u* of a certain PDE problem on $\Omega$. The main feature of these functionals is that the minimality of a domain $\Omega$ cannot be translated into a variational problem for a single (real or vector valued) state function. In this paper we focus on the case of affine cost functions $j(u,x)=-g(x)u+Q(x)$, where *u* is the solution of the PDE $-\Delta u=f$ with Dirichlet boundary conditions. We obtain the Lipschitz continuity and the non-degeneracy of the optimal *u* from the inwards/outwards optimality of $\Omega$ and then we use the stability of $\Omega$ with respect to variations with smooth vector fields in order to study the blow-up limits of the state function *u*. By performing a triple consecutive blow-up, we prove the existence of blow-up sequences converging to homogeneous stable solution of the one-phase Bernoulli problem and according to the blow-up limits, we decompose $\partial \Omega$ into a singular and a regular part. In order to estimate the Hausdorff dimension of the singular set of $\partial \Omega$ we give a new formulation of the notion of stability for the one-phase problem, which is preserved under blow-up limits and allows to develop a dimension reduction principle. Finally, by combining a higher order Boundary Harnack principle and a viscosity approach, we prove $C^\infty$ regularity of the regular part of the free boundary when the data are smooth.

## Introduction

This paper is dedicated to the regularity of the optimal shapes, solutions to shape optimization problems of the form

$$\begin{aligned} \text{ min }\big \{J(A)\ :\ A\in \mathcal {A}\big \}, \end{aligned}$$

where $\mathcal {A}$ is an admissible class of open, Lebesgue measurable or quasi-open subsets of $\mathbb {R}^d$, and where $J:\mathcal {A}\rightarrow \mathbb {R}$ is a given shape functional, with *J*(*A*) usually depending on the solution of a PDE on the domain *A*. This kind of minimization problems arise in different models in Biology, Engineering and Physics (see for example [6, 21] for an overview) and have been extensively studied from both numerical and theoretical points of view. In particular, there are two classes of shape optimization problems of the form above with long history, both leading to overdetermined elliptic PDE problems with Dirichlet boundary conditions.

The first class involves the so-called *spectral functionals*, that is, functionals depending on the eigenvalues of the Dirichlet Laplacian as

$$\begin{aligned} J(A)=\varphi \big (\lambda _1(A),\dots ,\lambda _k(A)\big )+|A|, \end{aligned}$$

where $\varphi :\mathbb {R}^k\rightarrow \mathbb {R}$ is a real-valued function and |*A*| denotes the Lebesgue measure of *A*. The associated shape optimization problems

$$\begin{aligned} \text{ min }\Big \{\varphi \big (\lambda _1(A),\dots ,\lambda _k(A)\big )+|A|\ :\ A\subset \mathbb {R}^d\Big \}, \end{aligned}$$

have a long history and are related to the classical question “Can one hear the shape of the drum?” and, more generally, to the interplay between the geometry of the domains and the spectrum of the Dirichlet Laplacian. The first results on the characterization of the optimal shapes, for the first and the second Dirichlet eigenvalues, go back to the works of Faber–Krahn (1922) and Krahn–Szegö (1923), and consist in finding explicit minimizers (balls and unions of disjoint balls), which is only possible in some special cases as $J=\lambda _1$ and $J=\lambda _2$. Today, thanks to theory developed by Buttazzo and Dal Maso [10], and to the more recent results [5, 30], it is well-known that, for monotone functionals $\varphi$, minimizers exist in a class of measurable (quasi-open) sets. The regularity of the optimal shapes has also been extensively studied; we refer to [4] and [34] for the case of optimal sets of $\lambda _1$ in a box, to [32] for the optimal sets of $\lambda _2$ in a box, and to [8, 25, 26, 31] (see also [15, 32]) for functionals involving higher eigenvalues $\lambda _k$.

The second class of functionals involves *integral shape functionals*, namely, for every bounded open set $A\subset \mathbb {R}^d$ we define

$$\begin{aligned} J(A):=\int _A j(u_A,x)\,dx, \end{aligned}$$

where the cost function

$$\begin{aligned} j:\mathbb {R}\times \mathbb {R}^d\rightarrow \mathbb {R}\, \end{aligned}$$

is fixed and the state function $u_A$ is the (weak) solution of the PDE

1.1 $$\begin{aligned} -\Delta u=f\quad \text {in}\quad A,\qquad u\in H^1_0(A), \end{aligned}$$

the force term $f:\mathbb {R}^d\rightarrow \mathbb {R}$ being also a prescribed function.

Optimization problems for integral shape functionals arise in Optimal Control and in models from Mechanics, in which the optimization criteria $j(u_A,x)$ takes into account external factors and forces that might appear not immediately, but only after the design is complete and the state function $u_A$ is already fixed. This type of problems pops up also in population dynamics, when one aims to optimize the population size. As in the case of spectral functionals, also for integral functionals with monotone cost, the existence of optimal shapes in bounded domains follows from the general theory of Buttazzo and Dal Maso, and again the solutions belong to the large class of measurable (or quasi-open) sets. This existence problem was studied, in a more general framework, in [13]. A general existence result in the class of open sets, was proved recently in [11] and in [12]. Precisely, it was shown that if *D* is a bounded open set in $\mathbb {R}^d$ and if the function *j* satisfies some suitable growth assumptions, then the shape optimization problem

$$\begin{aligned} \text{ min }\Big \{\int _A j(u_A,x)\,dx\ :\ A\ \text {open},\ A\subset D\Big \}, \end{aligned}$$

has a solution $\Omega \subset D$, $\Omega$-open.

On the other hand, even if the existence theory is quite well understood, there is no regularity theory for the minimizers of integral shape functionals, even in the simplest case

$$\begin{aligned} j(u,x)=-g(x)u+Q(x),\quad \text {with}\quad g\ne f, \end{aligned}$$

the regularity of the optimal sets was out of reach.

In this paper we prove the first general regularity result for the optimal shapes of integral functionals. In order to make our main result (Theorem 1.2) easier to read, we introduce the following definition.

### Definition 1.1

Let *D* be an open set in $\mathbb {R}^d$. For $k\in \mathbb {N}\setminus \{0\}$, $\alpha \in [0,1]$ and $N\in \mathbb {N}$, we call a set $\Omega \subset D$ $(k,\alpha , N)$-*regular in D* if the free boundary $\partial \Omega \cap D$ is the disjoint union of a regular part $Reg(\partial \Omega )$ and a (possibly empty) singular part $\text { Sing}(\partial \Omega )$ such that:

- $\text { Reg}(\partial \Omega )$ is a relatively open subset of $\partial \Omega \cap D$ and locally a graph of a $C^{k,\alpha }$-regular function;
- $\text { Sing}(\partial \Omega )$ is a closed subset of $\partial \Omega \cap D$ and has the following properties:
  - If $d<N$, then $\text { Sing}(\partial \Omega )$ is empty.
  - If $d\ge N$, then the Hausdorff dimension of $\text { Sing}(\partial \Omega )$ is at most $d-N$, namely
    $$\begin{aligned} \mathcal {H}^{d-N+{\varepsilon }}\big (\text { Sing}(\partial \Omega )\big )=0\quad \text {for every }{\varepsilon }>0. \end{aligned}$$

Moreover, if the regular part $\text { Reg}(\partial \Omega )$ is $C^\infty$, then we say that $\Omega$ is $(\infty , N)$-*regular in D*.

The main result of the present paper is the following.

### Theorem 1.2

Let *D* be a bounded open set in $\mathbb {R}^d$, where $d\ge 2$. Let

$$\begin{aligned} f:D\rightarrow \mathbb {R},\quad g:D\rightarrow \mathbb {R},\quad Q:D\rightarrow \mathbb {R}, \end{aligned}$$

be given non-negative functions. Suppose that the following conditions hold:

- $$f,g\in L^\infty (D)\cap C^2(D)$$;
- there are constants $C_1,C_2 > 0$ such that
  1.2 $$\begin{aligned} 0\le C_1 g\le f\le C_2 g\quad \text{ in }\quad D. \end{aligned}$$
- $Q\in C^{2}(D)$ and there are a positive constants $c_Q,C_Q$ such that
  $$\begin{aligned} 0<c_Q\le Q(x)\leqq C_Q\quad \text {for every}\quad x\in D. \end{aligned}$$

Then, there is $\alpha \in (0,1)$ such that every solution $\Omega \subset D$ to the shape optimization problem

1.3 $$\begin{aligned} \text{ min }\bigg \{\int _A \Big (-g(x)u_A+Q(x)\Big )\,dx\ :\ A\subset D,\ A\ \text{ open }\bigg \}, \end{aligned}$$

is $(1,\alpha ,5)$-regular in *D*. Moreover, if $f,g,Q\in C^\infty (D)$, then $\Omega$ is $(\infty ,5)$-regular in *D*.

### Proof

The definitions of the regular and the singular parts, $\text { Reg}(\partial \Omega )$ and $\text { Sing}(\partial \Omega )$, of the free boundary $\partial \Omega \cap D$ are given in Sect. 5. The $C^{1,\alpha }$ regularity of $\text { Reg}(\partial \Omega )$ is proved in Theorem 6.4, while the $C^\infty$ regularity follows from Proposition 6.5. The bounds on the dimension of $\text { Sing}(\partial \Omega )$ are given in Theorem 8.1. $\square$

### Remark 1.3

(On the bound on the dimension of the singular set $\text { Sing}(\partial \Omega )$) In Sect. 7 we develop a theory about the regularity of the stable global solutions of the one-phase Bernoulli problem (the definition of global stable solution is given in Definition 7.3); we show that there is a critical dimension $d^*$ (see Definition 7.7), in which a singular global solution appears for the first time (see Theorem 7.8), and we prove that the $d^*$ can be only 5, 6, or 7 (see Theorem 7.9). In Sect. 8 (Theorem 8.1) we use this results to show the following bounds on the singular part $\text { Sing}(\partial \Omega )$ of an optimal set $\Omega$, solution to (1.3):

- if $d<d^*$, then $\text { Sing}(\partial \Omega )$ is empty;
- if $d\ge d^*$, then the Hausdorff dimension of $\text { Sing}(\partial \Omega )$ is at most $d-d^*$.

In particular, since (by Theorem 7.9) $d^*\ge 5$, we get that:

- if $d<5$, then $\text { Sing}(\partial \Omega )$ is empty;
- if $d\ge 5$, then the Hausdorff dimension of $\text { Sing}(\partial \Omega )$ is at most $d-5$.

In other words, the precise statement of Theorem 1.2 is that under the conditions (a)–(b)–(c), the optimal sets are $(1,\alpha ,d^*)$-regular, where $d^*$ is the critical dimension from Definition 7.7.

### Remark 1.4

(On the assumptions (a)–(b)–(c) in Theorem 1.2) The $C^2$ regularity assumption in (a) is technical and is related to the use we make of the second order variations of the functional *J* along vector fields (see Sect. 2). The assumption (b) is used in the proofs of the Lipschitz continuity and the non-degeneracy of the state function $u_\Omega$; we notice that (b) is automatically satisfied when *f* and *g* are both bounded from above and from below by positive constants. The bounds from above and below on the weight *Q* in (c) are usual in the class of Bernoulli-type free boundary problems; these bounds are necessary for the Lipschitz continuity and the non-degeneracy of $u_\Omega$ (see Sect. 3), which are essential ingredients for the blow-up analysis in Sect. 4. The $C^2$ regularity of *Q*, on the other hand, is used again in the computation of the second variation in Sect. 2 and the passage to the blow-up limit in the proof of Theorem 8.1.

### Remark 1.5

(On the existence of optimal sets) In [11, Theorem 1.1] it was proved that if *D* is a bounded open subset of $\mathbb {R}^d$ and if *f*, *g*, *Q* satisfy the following conditions:

- $$f,g\in L^\infty (D)$$;
- there are positive constants $C_1\le C_2$ such that $0\le C_1g\le f\le C_2g$ in *D*;
- $$Q\in L^\infty (D)$$, $$Q\ge 0$$ in *D*,

then, there is an open set $\Omega \subset D$ solution to the shape optimization problem (1.3).

The presence of the inclusion constraint $\Omega \subset D$ is essential for the existence theory for general shape optimization problems (see for instance [10] and [11]). In the case of integral functionals with affine cost, as the one in (1.3), the inclusion constraint can be removed. In the next theorem, which we prove in Sect. 9, we show that optimal sets exist in $\mathbb {R}^d$. Moreover, we prove that the optimal sets in $\mathbb {R}^d$ are bounded, which implies that they are solutions to (1.3) in some sufficiently large ball $D:=B_R$, so the regularity of the free boundary in $\mathbb {R}^d$ is a consequence of Theorem 1.2.

### Theorem 1.6

In $\mathbb {R}^d$, $d\ge 2$, let $f,g,Q:\mathbb {R}^d\rightarrow \mathbb {R}$ be non-negative functions.

Suppose that the following conditions hold:

- $$f,g\in L^\infty (\mathbb {R}^d)\cap L^1(\mathbb {R}^d)\cap C^2(\mathbb {R}^d)$$ and that
  $$\begin{aligned} f(x)>0\quad \text{ and }\quad g(x)>0\quad \text{ for } \text{ every }\quad x\in \mathbb {R}^d. \end{aligned}$$
- $Q\in C^{2}(\mathbb {R}^d)$ and there are positive constants $c_Q,C_Q$ such that
  $$\begin{aligned} 0<c_Q\le Q(x)\leqq C_Q\quad \text {for every}\quad x\in \mathbb {R}^d. \end{aligned}$$

Then, there is an open set $\Omega \subset \mathbb {R}^d$, which is a solution to the shape optimization problem

1.4 $$\begin{aligned} \text{ min }\bigg \{\int _A \Big (-g(x)u_A+Q(x)\Big )\,dx\ :\ A\subset \mathbb {R}^d,\ A\ \text{ open },\ |A|<+\infty \bigg \}, \end{aligned}$$

and every solution $\Omega$ to (1.4) is bounded and $(1,\alpha ,5)$-regular in $\mathbb {R}^d$. Moreover, if $f,g,Q\in C^\infty (\mathbb {R}^d)$, then $\Omega$ is $(\infty ,5)$-regular in $\mathbb {R}^d$.

### Integral Shape Functionals in the Case $f=cg$

In this section we briefly discuss the case in which *f* and *g* are proportional, which is the only instance of integral functional studied in the literature. Precisely, we claim that if *f* and *g* are such that

$$\begin{aligned} g=\frac{1}{2\lambda ^2} f\quad \text {for some constant}\quad \lambda >0, \end{aligned}$$

the shape optimization problem (1.3) is equivalent to the Bernoulli free boundary problem

1.5 $$\begin{aligned} \text {min}\bigg \{\int _{D}\Big (\frac{1}{2}|\nabla u|^{2}-f(x)u+\lambda ^{2}Q(x)\mathbbm {1}_{\{u\ne 0\}}\Big )\,dx\ :\ u\in H^1_{0}(D)\bigg \}. \end{aligned}$$

Fix a solution $u\in H^1_0(D)$ to (1.5), for which the set $\{u\ne 0\}$ is open, and fix an optimal set $\Omega$ for (1.3) with a state function $u_\Omega$. Since *u* satisfies

$$\begin{aligned} -\Delta u=f\quad \text {in}\quad \{u\ne 0\}\,\qquad u\in H^{1}_{0}(\{u\ne 0\}), \end{aligned}$$

by integrating by parts, we have that

$$\begin{aligned} \int _{D}\Big (\frac{1}{2}|\nabla u|^{2}-f(x)u+\lambda ^2Q(x)\mathbbm {1}_{\{u\ne 0\}}\Big )\,dx&=\int _{D}\Big (-\frac{1}{2} f(x)u+\lambda ^2Q(x)\mathbbm {1}_{\{u\ne 0\}}\Big )\,dx\\  &=\lambda ^2\int _{\Omega _{u}}\big (-g(x)u+Q(x)\big )\,dx=\lambda ^2J(\{u\ne 0\})\,. \end{aligned}$$

Analogously, since $\{u_\Omega \ne 0\}\subset \Omega$ and *Q* is positive, we have

$$\begin{aligned} \int _{D}\Big (\frac{1}{2}|\nabla u_\Omega |^2-f(x)u_\Omega +\lambda ^2Q(x)\mathbbm {1}_{\{u_\Omega \ne 0\}}\Big )\,dx\le \lambda ^2 J(\Omega ). \end{aligned}$$

Thus, if *u* is a solution to (1.5), then $J(\{u\ne 0\})\le J(\Omega )$ and so, $\{u\ne 0\}$ is a solution to (1.3). Conversely, if $\Omega$ minimizes (1.3), then $u_\Omega$ is a minimizer of (1.5). Thus, for proportional *f* and *g*, the problem (1.3) is equivalent to (1.5); moreover, we notice that the argument above does not require the positivity of *f* and *g*, so the equivalence of the problems (1.5) and (1.3) holds also when *f* and *g* change sign.

The regularity of the solutions to the free boundary problem (1.5) is nowadays well-understood (at least when *Q* satisfies the condition (c) of Theorem 1.2). When $f\ge 0$, the regularity of $\partial \Omega$ follows from the regularity theory for the one-phase Bernoulli problem (see [2, 14, 18–20, 22, 39]). If the right-hand side *f* changes sign, then (1.5) becomes a two-phase Bernoulli problem, for which the regularity of the free boundary was obtained recently in [35] and [16].

Finally, we notice that when *f* and *g* are not proportional, the state function $u_\Omega$ of an optimal set $\Omega$ (that is, a solution to the shape optimization problem (1.3)) is not a minimizer of a free boundary functional as the one in (1.5). In particular, this implies that one can test the optimality of $u_\Omega$ only with functions ${\widetilde{u}}$ which are themselves state functions of some $\widetilde{\Omega }$. This means that a function ${\widetilde{u}}$, that differs from *u* only in a small ball $B_r$, cannot be used to test the optimality of $u_\Omega$ (truncations, harmonic replacements and radial extensions in small balls are not admitted), which makes most of the classical free boundary regularity results impossible to apply.

### Adjoint State and Optimality Condition on the Free Boundary

Let us go back to the general case when *f* and *g* are not proportional. We will show that the optimality condition on the boundary $\partial \Omega \cap D$ of an optimal open set $\Omega$ for (1.3) leads to a free boundary problem involving the state function $u_\Omega$. In order to see this, we introduce the adjoint state function $v_\Omega$ as follows: for every open set $A\subset D$ we will denote by $v_A$ the weak solution to the problem

1.6 $$\begin{aligned} -\Delta v_A=g\quad \text {in}\quad A,\qquad v_A\in H^1_0(A). \end{aligned}$$

By an integration by parts one can see that

$$\begin{aligned} \int _{D}gu_A\,dx=\int _D\nabla u_A\cdot \nabla v_A\,dx=\int _{D}fv_A\,dx, \end{aligned}$$

which means that the two state variables $u_A$ and $v_A$ are interchangeable. Precisely, an open set $\Omega \subset D$ is a solution to (1.3) if and only if it minimizes

$$\begin{aligned} \text{ min }\bigg \{\int _A \Big (-f(x)v_A+Q(x)\Big )\,dx\ :\ A\subset D,\ A\ \text{ open }\bigg \}. \end{aligned}$$

Sometimes, it is more convenient to consider simultaneously the two state functions, by using the following equivalent formulation, which is symmetric in $v_\Omega$ and $u_\Omega$

$$\begin{aligned} \text{ min }\bigg \{\int _A \Big (\nabla u_A\cdot \nabla v_A-f(x)v_A-g(x)u_A+Q(x)\Big )\,dx\ :\ A\subset D,\ A\ \text{ open }\bigg \}. \end{aligned}$$

Throughout the paper we will denote the functional from (1.3) by $\mathcal {F}$. Precisely, we set

1.7 $$\begin{aligned} \mathcal {F}(\Omega ;D):=\int _D \Big (-g(x)u_\Omega +Q(x)\mathbbm {1}_\Omega \Big )\,dx, \end{aligned}$$

which after an integration by parts has the symmetric form

$$\begin{aligned} \mathcal {F}(\Omega ;D)=\int _{D}\Big (\nabla u_\Omega \cdot \nabla v_\Omega \, - g(x)u_\Omega - f(x) v_\Omega +Q(x)\mathbbm {1}_\Omega \Big )\,dx, \end{aligned}$$

so if $\xi \in C^\infty _c(D;\mathbb {R}^d)$ is a smooth compactly supported vector field in *D* and $\Omega _t:=(Id+t\xi )(\Omega )$, then the first variation of $\mathcal {F}$ along $\xi$ is given by (see Lemma 2.6)

$$\begin{aligned} \delta \mathcal {F}(\Omega ;D)[\xi ]:&=\frac{\partial }{\partial t}\bigg |_{t=0}\mathcal {F}({\Omega _t},D)\\&=\int _\Omega \Big (\big (\nabla u_\Omega \cdot \nabla v_\Omega +Q(x) \big ){{\,\textrm{div}\,}}\xi -\nabla u_\Omega \cdot \big ((\nabla \xi )+(D\xi )\big )\nabla v_\Omega \Big )\,dx\\&\quad -\int _\Omega \Big (u_\Omega {{\,\textrm{div}\,}}(g\xi ) + v_\Omega {{\,\textrm{div}\,}}(f\xi )\Big )\,dx\,. \end{aligned}$$

Moreover, if $\Omega$ is a minimizer and $\partial \Omega$ is smooth, then an integration by parts gives that

$$\begin{aligned} \delta \mathcal {F}(\Omega ;D)[\xi ]=\int _{\partial \Omega } (\nu \cdot \xi )\big (Q-|\nabla u_\Omega ||\nabla v_\Omega |\big )\,d\mathcal H^{d-1}=0\quad \text {for every}\quad \xi \in C^\infty _c(D;\mathbb {R}^d), \end{aligned}$$

so the state functions $u_\Omega$ and $v_\Omega$ are (at least formally) solutions to the free boundary system

1.8 $$\begin{aligned} {\left\{ \begin{array}{ll} -\Delta u_\Omega =f\quad \text {in}\quad \Omega ,\\ -\Delta v_\Omega =g\quad \text {in}\quad \Omega ,\\ u_\Omega =v_\Omega =0\quad \text {and}\quad |\nabla u_\Omega ||\nabla v_\Omega |=Q\quad \text {on}\quad D\cap \partial \Omega . \end{array}\right. } \end{aligned}$$

#### Remark 1.7

An epsilon-regularity theorem for viscosity solutions of the above system was proved recently in [28]. Precisely, it was shown that if:

- $u_\Omega ,v_\Omega :D\rightarrow \mathbb {R}$ are continuous and non-negative functions such that
  $$\begin{aligned} \Omega =\{u_\Omega>0\}=\{v_\Omega >0\}; \end{aligned}$$
- $(\Omega ,u_\Omega ,v_\Omega )$ is a viscosity solutions to (1.8) ;
- $u_\Omega$ and $v_\Omega$ are ${\varepsilon }$-*flat* (in a suitable sense) in a ball $B_r(x_0)\subset D$ centered on $\partial \Omega \,$;

then the free boundary $\partial \Omega$ is $C^{1,\alpha }$-regular in $B_{{r}/2}(x_0)$.

We notice that an epsilon-regularity theorem for a similar free boundary system, with $f\equiv g\equiv 0$, was studied in [1] in the context of a different shape optimization problem. Precisely, in [1], by using the minimality of $\Omega$, it was shown that it is an NTA domain, so by the Boundary Harnack Principle for harmonic functions, the system reduces to a one-phase problem for the function $u_\Omega$, for which the epsilon-regularity theorem is known by [2] and [18]. In Sect. A we will detail how our results can be applied to the problem of [1].

#### Remark 1.8

The free boundary system (1.8) emphasizes that, despite the use of the adjoint variable $v_\Omega$, it is not possible to reformulate the shape optimization problem (1.3) in terms of an integral functional depending on $u_\Omega$ or the couple $(u_\Omega ,v_\Omega )$. Precisely, no functional of the form

$$\begin{aligned} \int F(\nabla u, \nabla v, u, v, x)+ Q(x)\mathbbm {1}_{\{u^2+v^2>0\}}\,dx\, \end{aligned}$$

has Euler-Lagrange equations given by (1.8). The same holds for the equations satisfied by the blow-up limits of $(u_\Omega ,v_\Omega )$. Indeed, since the couple $(u_\Omega ,v_\Omega )$ satisfies (1.8), any 1-homogeneous blow-up limit $(u_0,v_0)$ would be a solution of the same system with $f=g\equiv 0$ and $Q\equiv const\,>0$, but this system is not the Euler-Lagrange system of an integral functional of the above type. This peculiarity represents one of the major challenges for studying the regularity of the free boundary and requires rewriting the optimality and stability conditions in terms of inner variations.

### Regularity of the Free Boundary and Dimension of the Singular Set

The key steps in the proof of Theorem 1.2 are the following:

- Prove that if $\Omega$ is an optimal set for (1.3), then it is a viscosity solution to (1.8). This, in combination with the epsilon-regularity result cited in Remark 1.7, will imply that the *flat* part of the free boundary is smooth.
- Show that there exists a critical dimension $d^*\in \{5,6,7\}$ such that in dimension $d< d^*$ all the points on $\partial \Omega \cap D$ are regular.
- Prove that a Federer-type dimension reduction principle holds for solutions to (1.3).

There are two main difficulties in following the program outlined in the three points above.

The first difficulty is in the fact that the first order optimality condition

$$\begin{aligned} \delta \mathcal {F}(\Omega ;D)[\xi ]=0\quad \text {for every}\quad \xi \in C^\infty _c(D;\mathbb {R}^d), \end{aligned}$$

is not leading to a monotonicity formula for $u_\Omega$ and $v_\Omega$; in particular, we do not know if the blow-up limits of $u_\Omega$ and $v_\Omega$ are in general homogeneous.

The second obstruction comes from the impossibility to make external perturbations of $u_\Omega$ and $v_\Omega$ (that is, in light of Remark 1.8 no perturbations of the form ${\widetilde{u}}=u_\Omega +\phi$ and ${\widetilde{v}}=v_\Omega +\psi$ are allowed), so the only information conserved along blow-up sequences is the one contained in the internal variations of $\Omega$ along smooth vector fields.

We overcome these difficulties by using the first and the second variation of $\mathcal {F}$. Indeed, suppose that $\Omega$ is optimal in *D* and consider the flow $\Phi _t$ associated to a compactly supported vector field $\xi \in C^\infty _c(D;\mathbb {R}^d)$. Then, setting $\Omega _t:=\Phi _t(\Omega )$, we get that the function

$$\begin{aligned} t\mapsto \mathcal {F}(\Omega _t;D), \end{aligned}$$

has a minumum in $t=0$, so we have

1.9 $$\begin{aligned} \frac{\partial }{\partial t}\Big |_{t=0}\mathcal {F}(\Omega ;D)=0\qquad \text {and}\qquad \frac{\partial ^2}{\partial t^2}\Big |_{t=0}\mathcal {F}(\Omega ;D)\ge 0, \end{aligned}$$

that is, $\Omega$ is a stable critical point of the shape functional

$$\begin{aligned} \Omega \mapsto \mathcal {F}(\Omega ;D). \end{aligned}$$

We prove that this notion is stable under blow-up limits at free boundary points $x_0\in \partial \Omega$:

$$\begin{aligned} u_0(x)=\lim _{n\rightarrow \infty }\frac{u_\Omega (x_0+r_nx)}{r_n}\qquad \text {and}\qquad v_0(x)=\lim _{n\rightarrow \infty }\frac{v_\Omega (x_0+r_nx)}{r_n}. \end{aligned}$$

Thus $\Omega _0=\{u_0>0\}=\{v_0>0\}$ is a stable critical point for the same functional, but this time with $f\equiv g\equiv 0$ and $Q\equiv Q(x_0)$. Now, since $u_0,v_0:\mathbb {R}^d\rightarrow \mathbb {R}$ are harmonic on $\Omega _0$, we can apply the Boundary Harnack Principle from [29] to obtain that the ratio

$$\begin{aligned} \frac{u_0}{v_0}:\Omega _0\rightarrow \mathbb {R}\end{aligned}$$

is Hölder continuous in $\Omega _0$, up to $\partial \Omega _0$. After a second blow-up (this time in zero), we get that the positivity set $\Omega _{00}$ of the functions

$$\begin{aligned} u_{00}(x)=\lim _{m\rightarrow \infty }\frac{u_{0}(r_mx)}{r_m}\qquad \text {and}\qquad v_{00}(x)=\lim _{m\rightarrow \infty }\frac{v_{0}(r_mx)}{r_m}, \end{aligned}$$

is a stable critical point (in every ball $B_R\subset \mathbb {R}^d$) for the functional $\mathcal {F}(\cdot ,B_R)$, still with $f\equiv g\equiv 0$ and $Q\equiv Q(x_0)$. Moreover, by the Boundary Harnack Principle, we also have that $u_{00}$ and $v_{00}$ are proportional. Thus, $u_{00}$ is (up to a constant) a stable critical point (in the sense of (1.9)) for the Alt–Caffarelli’s one-phase functional

$$\begin{aligned} \mathcal G(u;B_R):=\int _{B_R}|\nabla u|^2\,dx+|\{u>0\}\cap B_R|\quad \text {in every ball}\quad B_R\subset \mathbb {R}^d, \end{aligned}$$

so the third blow-up in zero

$$\begin{aligned} u_{000}(x)=\lim _{k\rightarrow \infty }\frac{u_{00}(r_kx)}{r_k}\, \end{aligned}$$

is a 1-homogeneous stable critical point for the Alt–Caffarelli’s functional $\mathcal G$.

The existence of a homogeneous blow-up $u_{000}$ is a key element in the proof of Theorem 1.2. Indeed, it allows to prove (see Proposition 6.3) that the state functions $u_\Omega$ and $v_\Omega$ are viscosity solutions to the system (1.8) by showing that if we take a free boundary point admitting a one-sided tangent ball, then the homogeneous blow-up limits $u_{000}$ and $v_{000}$ constructed above are half-plane solutions. This, in combination with the epsilon-regularity theorem cited in Remark 1.7, implies that, in a neighborhood of any boundary point admitting a half-plane solution as blow-up limit, the free boundary is $C^{1,\alpha }$-regular; we will call these points *regular points* (see Sect. 5), while the remaining part of the free boundary (if any) will be called *singular*. Finally, we notice that the smoothness of the regular part requires only the criticality of $\Omega$ (the first part of (1.9)).

It is natural to expect that the stability of $\Omega$ (the second part of (1.9)) leads to an estimate on the dimension of the singular set; in fact, the bounds on the critical dimension (the dimension in which a singularity appears for the first time) for minimizers of the one-phase Alt–Caffarelli functional rely (see [14] and [22]) on the well-known *stability inequality* of Caffarelli-Jerison-Kenig, which was originally obtained in [14] through a particular second order variation of the functional $\mathcal G$. On the other hand, this stability inequality is not easy to handle when it comes to passing to blow-up limits and developing a dimension reduction principle. In Sect. 7, we give a different formulation of the stability, which uses the second variation along vector fields, as defined in (1.9). Again in Sect. 7, we show that our notion of stability allows to develop a dimension reduction principle and that there is a critical dimension $d^*$, which is the smallest dimension admitting one-homogeneous stable solutions with singularity (see Theorem 7.8). Then, in Proposition 7.12, we prove that on smooth cones (that is, cones with isolated singularity) our notion of stability is equivalent to the stability inequality of Caffarelli-Jerison-Kenig. Thus, we obtain the bound $5\le d^*\le 7$ on the critical dimension $d^*$ as a consequence of the results of Jerison-Savin [22] and De Silva-Jerison [19]. Finally, in Theorem 8.1, we prove the bounds on the singular set from Theorem 1.2 by (again) a triple blow-up argument, which allows to transfer the dimension bounds from Theorem 7.8 to the singular set of the solutions to (1.3).

#### Remark 1.9

The methodology we developed for the analysis of (1.3) can be applied, with some natural adjustments, to various free boundary and shape optimization problems. In fact, once the local behavior of the state variables close to the free boundary is known, the triple blow-up analysis combined with the notion of stability along domain variations can be applied in an almost straightforward manner. We mention some recent contributions where the authors leveraged our methodology, adapting it to different problems, see [7, 23, 33]. In particular, in [7] the ${\varepsilon }$-regularity theory was applied to a spectral shape optimization problem, while in [33] and [23] our approach to the stable critical points was applied to free boundary problems.

### Plan of the Paper

In Sect. 2 we compute the first and second variations of the functional $\mathcal {F}$. In Sect. 3 we prove the non-degeneracy and Lipschitz continuity of the state variables. In Sect. 4 we perform a blow-up analysis by proving the existence of triple blow-up sequences converging to a homogeneous limit.

In Sect. 5, we use the blow-up limits from Sect. 4 to define the decomposition of the free boundary into a regular part and a singular part.

In Sect. 6 we prove that the state functions $u_\Omega , v_\Omega$ of an optimal set $\Omega$ for (1.3) are viscosity solutions of the free boundary system (1.8). Using this information, in Theorem 6.4, we prove that the regular part of the free boundary is $C^{1,\alpha }$-smooth.

In Sect. 7 we define the notion of a global stable solution to the one-phase Bernoulli (Alt–Caffarelli) problem and we study the dimension of the singular sets for these global stable solutions. We notice that this section can also be read indipendently and that, together with Sect. 2, it contains the key results for the analysis of the singular set.

In Sect. 8 we use the first and the second variations from Sect. 2, the triple blow-up procedure from Sect. 4 and the theory from Sect. 7 in order to prove the dimension bounds on the singular set. This concludes the proof of Theorem 1.2, which follows from Theorem 8.1, Theorem 6.4 and Proposition 6.5.

In Sect. 9 we address the existence of optimal sets in $\mathbb {R}^d$ and we prove Theorem 1.6. Ultimately, in Appendix A we apply the analysis of Sects. 7 and 8 to optimal sets arising sets arising from the heat conduction problem studied in [1].

### Notations

In the whole paper we use the notation

$$\begin{aligned} B_r(x_0)=\{x\in \mathbb {R}^d: |x-x_0|<r\}, \end{aligned}$$

for the ball of radius *r* centered at point $x_0$ and, when $x_0=0$, we write $B_r=B_r(0)$; we denote by $\omega _d$ the Lebesgue measure of a ball of radius one in $\mathbb {R}^d$. For any set $A\subset \mathbb {R}^d$, we set

$$\begin{aligned} \mathbbm {1}_A(x):={\left\{ \begin{array}{ll} 1, & \text {if }\ x\in A,\\ 0, & \text {if }\ x\in \mathbb {R}^d\setminus A. \end{array}\right. } \end{aligned}$$

Given a non-negative function *u* we often denote its positivity set as $\Omega _u:=\{u>0\}$.

We denote by $H^1(\mathbb {R}^d)$ the set of Sobolev functions in $\mathbb {R}^d$, that is, the closure of the smooth functions with bounded support $C^\infty _c(\mathbb {R}^d)$ with respect to the usual Sobolev norm

$$\begin{aligned} \Vert \varphi \Vert _{H^1}^2=\int _{\mathbb {R}^d}\big (|\nabla \varphi |^2+\varphi ^2\big )\,dx. \end{aligned}$$

Given an open set $\Omega \subset \mathbb {R}^d$, we define the Sobolev space $H^1_0(\Omega )$ as the closure, with respect to $\Vert \cdot \Vert _{H^1}$, of the space $C^\infty _c(\Omega )$ of smooth functions compactly supported in $\Omega$. Thus, every $u\in H^1_0(\Omega )$ is identically zero outside $\Omega$ and we have the inclusion $H^1_0(\Omega )\subset H^1(\mathbb {R}^d)$.

We will sometimes use the following notation for minimum and maximum of two real numbers:

$$\begin{aligned} \text {min}\{a,b\}=a\wedge b,\qquad \max \{a,b\}=a\vee b,\qquad a,b\in \mathbb {R}. \end{aligned}$$

Given a function $u:\mathbb {R}^d\rightarrow \mathbb {R}$ we will denote by $\nabla u$ and *Du* the vectors column and row with components the partial derivatives of *u*, while $D^2u$ will be the Hessian matrix of *u*. Given a vector field $F:\mathbb {R}^d\rightarrow \mathbb {R}^d$ with components $F_k$, $k=1,\dots ,d$ we will denote by $\nabla F$ the $d\times d$ matrix with columns $\nabla F_k$, $k=1,\dots , d$ and rows $(\partial _jF_1,\partial _jF_2,\dots ,\partial _jF_d)$. By convention $DF:=(\nabla F)^T$, where for any matrix $M\in \mathbb {R}^{d\times d}$, we will denote by $M^T$ its transpose. Given a vector field $V:\mathbb {R}^d\rightarrow \mathbb {R}^d$ and a matrix with variable coefficients $M=(m_{ij})_{ij}:\mathbb {R}^d\rightarrow \mathbb {R}^{d\times d}$ we will denote by $(V\cdot \nabla )(M)$ the $d\times d$ matrix with variable coefficients $V\cdot \nabla m_{ij}$.

## First and Second Variations Under Inner Perturbations

In this section we compute the first and the second variations (with respect to perturbations with compactly supported vector fields) of the functional $\mathcal {F}$ from (1.7). Both variations will be fundamental tools in the study of the blow-up limits of the state variables $u_\Omega$ and $v_\Omega$ on a domain $\Omega$, which is optimal for (1.3).

### First and Second Variation of the State Function

In Lemma 2.5 we compute the expansion of the state variable $u_\Omega$ (solution to (1.1)) with respect to smooth perturbations of a set $\Omega$. We first prove Lemma 2.2 and Lemma 2.3, where we compute the expansion of a one-parameter family of solutions to PDEs on the same domain $\Omega$.

#### Remark 2.1

In what follows we will denote by $\mathbb {R}^{d\times d}$ the space of $d\times d$ square matrices with real coefficients. Given a real matrix $A=(a_{ij})_{ij}\in \mathbb {R}^{d\times d}$, we define its norm in the space $\mathbb {R}^{d\times d}$ as

$$\begin{aligned} \Vert A\Vert _{\mathbb {R}^{d\times d}}:=\bigg (\sum _{i=1}^d\sum _{j=1}^da_{ij}^2\bigg )^{1/2}, \end{aligned}$$

and we notice that for every vector $V\in \mathbb {R}^d$, we have $|AV|\le \Vert A\Vert _{\mathbb {R}^{d\times d}}|V|$, where |*V*| is the usual Euclidean norm of *V*. Next, let $\Omega$ be a measurable set in $\mathbb {R}^d$. Given a matrix $A:\Omega \rightarrow \mathbb {R}^{d\times d}$ with variable coefficients $a_{ij}:\Omega \rightarrow \mathbb {R}$, we say that

$$\begin{aligned} A\in L^\infty (\Omega ;\mathbb {R}^{d\times d}), \end{aligned}$$

if $a_{ij}\in L^\infty (\Omega )$ for every $1\le i,j\le d$. We define the norm $\Vert \cdot \Vert _{L^\infty (\Omega ;\mathbb {R}^{d\times d})}$ as

$$\begin{aligned} \Vert A\Vert _{L^\infty (\Omega ;\mathbb {R}^{d\times d})}:=\big \Vert \Vert A\Vert _{\mathbb {R}^{d\times d}}\big \Vert _{L^\infty (\Omega )}=\bigg \Vert \sum _{i=1}^d\sum _{j=1}^da_{ij}^2\bigg \Vert _{L^\infty (\Omega )}^{1/2}. \end{aligned}$$

#### Lemma 2.2

(First order expansion of solutions to PDEs) Let $\Omega$ be a bounded open set in $\mathbb {R}^d$. Let the functions

$$\begin{aligned} f:&\mathbb {R}\rightarrow L^2(\Omega ),&t\mapsto f_t,\\ A:&\mathbb {R}\rightarrow L^\infty (\Omega ;\mathbb {R}^{d\times d}),&t\mapsto A_t, \end{aligned}$$

be such that:

- $A_t(x)$ is a symmetric matrix for every $(t,x)\in \mathbb {R}\times \Omega$ and there is a symmetric matrix $\delta A\in L^\infty (\Omega ;\mathbb {R}^{d\times d})$ such that
  $$\begin{aligned} A_t=\text { Id}+t(\delta A)+o(t)\quad \text {in }L^\infty (\Omega ;\mathbb {R}^{d\times d}). \end{aligned}$$
- there is $\delta f\in L^2(\Omega )$ such that
  $$\begin{aligned} f_t=f_0+t(\delta f)+o(t)\quad \text {in }L^2(\Omega ). \end{aligned}$$

Then, for every *t* small enough there is a unique solution $u_t$ to the problem

2.1 $$\begin{aligned} -{{\,\textrm{div}\,}}(A_t\nabla u_t)=f_t\quad \text {in }\Omega ,\qquad u_t\in H^1_0(\Omega ), \end{aligned}$$

and

$$\begin{aligned} u_t=u_0+t(\delta u)+o(t)\quad \text {in }H^1_0(\Omega ), \end{aligned}$$

where $\delta u$ is the unique weak solution in $H^1_0(\Omega )$ to the PDE

2.2 $$\begin{aligned} -\Delta (\delta u)-{{\,\textrm{div}\,}}((\delta A)\nabla u_0)=\delta f\quad \text {in }\Omega ,\qquad \delta u\in H^1_0(\Omega ). \end{aligned}$$

#### Proof

Clearly $u_0\in H^1_0(\Omega )$ is the solution to $-\Delta u_0=f_0$ in $\Omega$. We set $w_t:=\frac{1}{t}(u_t-u_0)$. We will prove that $w_t$ converges to $\delta u$ strongly in $H^1_0(\Omega )$.

We notice that (2.1) can be written as

$$\begin{aligned} -{{\,\textrm{div}\,}}((\text { Id}+(A_t-\text { Id}))\nabla (u_0+tw_t))=f_0+(f_t-f_0)\quad \text {in}\quad \Omega . \end{aligned}$$

So, using the equation for $u_0$ and dividing by *t*, we get

2.3 $$\begin{aligned} -\Delta w_t-{{\,\textrm{div}\,}}\Big (\frac{1}{t}(A_t-\text { Id})\nabla u_0\Big )-{{\,\textrm{div}\,}}\Big ((A_t-\text { Id})\nabla w_t\Big )=\frac{1}{t}(f_t-f_0)\qquad \text {in }\Omega .\nonumber \\ \end{aligned}$$

If we fix ${\varepsilon }>0$, we can choose *t* small enough such that

$$\begin{aligned}  &   \Vert A_t-\text { Id}\Vert _{L^\infty (\Omega ;\mathbb {R}^{d\times d})}\le {\varepsilon }\,\quad \left\| \frac{1}{t}(A_t-\text { Id})-\delta A\right\| _{L^\infty (\Omega ;\mathbb {R}^{d\times d})}\\  &   \le {\varepsilon }\,\quad \left\| \frac{1}{t}(f_t-f_0)-\delta f\right\| _{L^2(\Omega )}\le {\varepsilon }. \end{aligned}$$

Thus, by testing (2.3) with $w_t$, we obtain

$$\begin{aligned} \int _\Omega |\nabla w_t|^2\,dx=\,&-\int _\Omega \nabla w_t\cdot \frac{1}{t}(A_t-\text { Id})\nabla u_0\,dx\\&-\int _\Omega \nabla w_t\cdot (A_t-\text { Id})\nabla w_t\,dx+\int _\Omega \frac{1}{t}(f_t-f_0)w_t\,dx\\ \le&\Big ({\varepsilon }+\Vert \delta A\Vert _{L^\infty (\Omega ;\mathbb {R}^{d\times d})}\Big )\Vert \nabla w_t\Vert _{L^2}\Vert \nabla u_0\Vert _{L^2}\\&\quad +{\varepsilon }\Vert \nabla w_t\Vert _{L^2}^2+\Big ({\varepsilon }+\Vert \delta f\Vert _{L^2(\Omega )}\Big )\Vert w_t\Vert _{L^2}. \end{aligned}$$

Now, by the Poincaré inequality,

$$\begin{aligned} \Vert \varphi \Vert _{L^2(\Omega )}^2\le C_d|\Omega |^{{2}/{d}}\Vert \nabla \varphi \Vert _{L^2(\Omega )}^2\quad \text {for every}\quad \varphi \in H^1_0(\Omega ), \end{aligned}$$

and the equation for $u_0$, we have that

$$\begin{aligned} \Vert \nabla u_0\Vert _{L^2(\Omega )}^2=\int _\Omega f_0u_0\,dx\le \Vert f_0\Vert _{L^2(\Omega )}\Vert u_0\Vert _{L^2(\Omega )}\le \Vert f_0\Vert _{L^2(\Omega )}C_d|\Omega |^{{1}/{d}}\Vert \nabla u_0\Vert _{L^2(\Omega )}, \end{aligned}$$

which gives the bound

$$\begin{aligned} \Vert \nabla u_0\Vert _{L^2(\Omega )}\le C_d|\Omega |^{{1}/{d}}\Vert f_0\Vert _{L^2(\Omega )}. \end{aligned}$$

Thus, we deduce that

$$\begin{aligned} \int _\Omega |\nabla w_t|^2\,dx&\le C_d|\Omega |^{{1}/{d}}\Vert f_0\Vert _{L^2(\Omega )}\Big ({\varepsilon }+\Vert \delta A\Vert _{L^\infty (\Omega ;\mathbb {R}^{d\times d})}\Big )\Vert \nabla w_t\Vert _{L^2}\\&\qquad +{\varepsilon }\Vert \nabla w_t\Vert _{L^2}^2+\Big ({\varepsilon }+\Vert \delta f\Vert _{L^2(\Omega )}\Big )C_d|\Omega |^{{1}/{d}}\Vert \nabla w_t\Vert _{L^2}, \end{aligned}$$

and so, for *t* (and ${\varepsilon }<1$) small enough

$$\begin{aligned}  &   \left( \int _\Omega |\nabla w_t|^2\,dx\right) ^{1/2}\\  &   \quad \le \frac{C_d|\Omega |^{{1}/{d}}}{(1-{\varepsilon })}\Big (1+\Vert f_0\Vert _{L^2(\Omega )}+\Vert f_0\Vert _{L^2(\Omega )}\Vert \delta A\Vert _{L^\infty (\Omega ;\mathbb {R}^{d\times d})}+\Vert \delta f\Vert _{L^2(\Omega )}\Big ). \end{aligned}$$

Thus, for every sequence $t_n\rightarrow 0$, there is a subsequence for which $w_{t_n}$ converges as $n\rightarrow \infty$, strongly in $L^2(\Omega )$ and weakly in $H^1_0(\Omega )$, to some function $w_\infty$. Passing to the limit the Eq. (2.3) we get that $w_\infty$ is also a solution to (2.2). Thus $w_\infty =\delta u$. In particular, this implies that $w_t$ converges as $t\rightarrow 0$, strongly in $L^2(\Omega )$ and weakly in $H^1_0(\Omega )$, to $\delta u$. Finally, in order to prove that the convergence is strong, we test again (2.3) with $w_t$:

$$\begin{aligned} \limsup _{t\rightarrow \infty }\int _\Omega |\nabla w_t|^2\,dx&=\lim _{t\rightarrow 0}\int _\Omega \nabla w_t\cdot \frac{1}{t}(A_t-Id)\nabla u_0\,dx+\lim _{t\rightarrow 0}\int _\Omega \frac{1}{t}(f_t-f_0)w_t\,dx\\&=\int _\Omega \nabla (\delta u)\cdot \delta A\nabla u_0\,dx+\int _\Omega \delta f\delta u\,dx=\int _\Omega |\nabla (\delta u)|^2\,dx\,. \end{aligned}$$

Combining this estimate with the lower semi-continuity of the $H^1$ norm, we get

$$\begin{aligned} \lim _{t\rightarrow \infty }\int _\Omega |\nabla w_t|^2\,dx=\int _\Omega |\nabla (\delta u)|^2\,dx\, \end{aligned}$$

which implies that $w_t$ converges to $\delta u$ strongly in $H^1_0(\Omega )$. $\square$

#### Lemma 2.3

(Second order expansion of solutions to PDEs) Let $\Omega$ be a bounded open set in $\mathbb {R}^d$. Let the functions

$$\begin{aligned} f:\mathbb {R}\rightarrow L^2(\Omega )\quad \text {and}\quad A:\mathbb {R}\rightarrow L^\infty (\Omega ;\mathbb {R}^{d\times d}) \end{aligned}$$

be such that:

- $A_t(x)$ is a symmetric matrix for every $(t,x)\in \mathbb {R}\times \Omega$ and there are symmetric matrices $\delta A\in L^\infty (\Omega ;\mathbb {R}^{d\times d})$ and $\delta ^2A\in L^\infty (\Omega ;\mathbb {R}^{d\times d})$ such that
  $$\begin{aligned} A_t=\text { Id}+t\,\delta A+t^2\delta ^2A+o(t^2)\quad \text {in }L^\infty (\Omega ;\mathbb {R}^{d\times d}); \end{aligned}$$
- there are $\delta f\in L^2(\Omega )$ and $\delta ^2f\in L^2(\Omega )$ such that
  $$\begin{aligned} f_t=f_0+t\,\delta f+t^2\delta ^2f+o(t^2)\quad \text {in }L^2(\Omega ). \end{aligned}$$

Then, for every *t* small enough there is a unique solution $u_t$ to the problem

$$\begin{aligned} -{{\,\textrm{div}\,}}(A_t\nabla u_t)=f_t\quad \text {in }\Omega ,\qquad u_t\in H^1_0(\Omega ), \end{aligned}$$

and

$$\begin{aligned} u_t=u_0+t\,\delta u+t^2\delta ^2u+o(t^2)\quad \text {in }H^1_0(\Omega ), \end{aligned}$$

where $\delta u\in H^1_0(\Omega )$ is the solution to (2.2) and where $\delta ^2u\in H^1_0(\Omega )$ solves the PDE

2.4 $$\begin{aligned} -\Delta (\delta ^2 u)={{\,\textrm{div}\,}}((\delta A)\nabla (\delta u))+{{\,\textrm{div}\,}}((\delta ^2 A)\nabla u_0)+\delta ^2 f\quad \text {in }\Omega , \quad \delta ^2 u\in H^1_0(\Omega ).\nonumber \\ \end{aligned}$$

#### Proof

Let $w_t:=\frac{1}{t}(u_t-u_0)$ be as in the proof of Lemma 2.2. We set

$$\begin{aligned} v_t:=\frac{1}{t}(w_t-\delta u)\in H^1_0(\Omega ). \end{aligned}$$

We will prove that $v_t$ converges strongly in $H^1_0(\Omega )$ to $\delta ^2u$. From the equation for $w_t$, we have

$$\begin{aligned} -\Delta (\delta u+tv_t)-{{\,\textrm{div}\,}}\Big (\frac{1}{t}(A_t-\text { Id})\nabla u_0\Big )-{{\,\textrm{div}\,}}\Big ((A_t-\text { Id})\nabla (\delta u+tv_t)\Big )=\frac{1}{t}(f_t-f_0). \end{aligned}$$

Thus, using the Eq. (2.2) for $\delta u$ ($-\Delta (\delta u)-{{\,\textrm{div}\,}}(\delta A\nabla u_0)=\delta f$), we get

$$\begin{aligned}&-\Delta v_t-{{\,\textrm{div}\,}}\Big (\frac{1}{t^2}(A_t-\text { Id}-t\delta A)\nabla u_0\Big )\\&\quad -{{\,\textrm{div}\,}}\Big (\frac{1}{t}(A_t-\text { Id})\nabla (\delta u+tv_t)\Big )=\frac{1}{t^2}(f_t-f_0-t\delta f). \end{aligned}$$

Now, reasoning as in Lemma 2.2, we get that the family $v_t$ is uniformly bounded in $H^1_0(\Omega )$ and converges as $t\rightarrow 0$ strongly in $L^2(\Omega )$ and weakly in $H^1_0(\Omega )$ to the solution $\delta ^2u$ of (2.4). In order to obtain the strong $H^1$ convergence, we compute

$$\begin{aligned} \limsup _{t\rightarrow \infty }\int _\Omega |\nabla v_t|^2\,dx&=-\lim _{t\rightarrow 0}\int _\Omega \nabla v_t\cdot \frac{1}{t^2}(A_t- \text{ Id }-t\delta A)\nabla u_0\,dx\\  &\qquad -\lim _{t\rightarrow 0}\int _\Omega \nabla v_t\cdot \frac{1}{t}(A_t- \text{ Id})\nabla (\delta u)\,dx\\  &\qquad +\lim _{t\rightarrow 0}\int _\Omega \frac{1}{t^2}(f_t-f_0-t\delta f)v_t\,dx\\  &=-\int _\Omega \nabla (\delta ^2 u)\cdot (\delta ^2 A)\nabla u_0\,dx\\  &\qquad -\int _\Omega \nabla (\delta ^2u)\cdot (\delta A)\nabla (\delta u)\,dx+\int _\Omega (\delta ^2 f)(\delta ^2 u)\,dx\\  &=\int _\Omega |\nabla (\delta ^2 u)|^2\,dx\,, \end{aligned}$$

which concludes the proof. $\square$

#### Remark 2.4

We recall that if $M=(m_{ij})_{1\le i,j\le d}\in \mathbb {R}^{d\times d}$ is a matrix with constant coefficients, then the following Taylor expansions in $\mathbb {R}^{d\times d}$ (with respect to the norm $\Vert \cdot \Vert _{\mathbb {R}^{d\times d}}$)

$$\begin{aligned}  &   (\text { Id}+tM)^{-1}=\text { Id}-tM+t^2M^2+o(t^2), \\  &   \det (\text { Id}+tM)=1+t\,\text { tr}(M)+\frac{t^2}{2}\Big (\big (\text { tr}(M)\big )^2-\text { tr}(M^2)\Big )+o(t^2), \end{aligned}$$

where $\text { Id}$ is the identity matrix in $\mathbb {R}^{d\times d}$ and $\text { tr}(M)$ is the trace $\text { tr}(M):=\sum _{i=1}^dm_{ii}$. As a consequence, if $M\in L^\infty (\Omega ;\mathbb {R}^{d\times d})$ is a matrix with variable coefficients, then we have the expansions

$$\begin{aligned} (\text { Id}+tM)^{-1}= &   \text { Id}-tM+t^2M^2+o(t^2)\quad \text {in }L^\infty (\Omega ;\mathbb {R}^{d\times d}), \\ \det (\text { Id}+tM)= &   1+t\,\text { tr}(M)+\frac{t^2}{2}\Big (\big (\text { tr}(M)\big )^2-\text { tr}(M^2)\Big )\\  &   +o(t^2)\quad \text {in }L^\infty (\Omega ;\mathbb {R}^{d\times d}). \end{aligned}$$

In particular, this implies that given two matrices $M,N\in L^\infty (\Omega ;\mathbb {R}^{d\times d})$, we have:

$$\begin{aligned} \big (\text { Id}+tM+t^2N+o(t^2)\big )^{-1}&=\text { Id}-tM+t^2(M^2-N)+o(t^2)\,;\\ \det \big (\text { Id}+tM+t^2N+o(t^2)\big )&=\det (\text { Id}+tM)\det (\text { Id}+t^2N)+o(t^2)\\&=1+t\,\text { tr}(M)+\frac{t^2}{2}\Big (\big (\text { tr}(M)\big )^2-\text { tr}(M^2)+2\text { tr}(N)\Big )+o(t^2)\,, \end{aligned}$$

and so,

$$\begin{aligned}&\big (\text { Id}+tM+t^2N+o(t^2)\big )^{-1}\big (\text { Id}+tM+t^2N+o(t^2)\big )^{-T}\\&\det \big (\text { Id}+tM+t^2N+o(t^2)\big )\\&\quad =\text { Id}+t\Big (-M-M^T+\text { tr}(M)\text { Id}\Big )\\&\quad \quad +t^2\Big (MM^T+M^2+(M^{T})^2-(N+N^T)-(M+M^T)\,\text { tr}(M)\Big )\\&\quad \quad +\frac{t^2}{2}\Big (\big (\text { tr}(M)\big )^2-\text { tr}(M^2)+2\text { tr}(N)\Big )\text { Id}+o(t^2). \end{aligned}$$

#### Proposition 2.5

Let *D* be a bounded open set in $\mathbb {R}^d$ and let $f \in C^2(D)$ with $f\ge 0$ in *D*. Given an open set $\Omega \subset D$ and a compactly supported vector field $\xi \in C^\infty _c(D;\mathbb {R}^d)$, we consider the associated flow $\Phi :\mathbb {R}\times \mathbb {R}^d\rightarrow \mathbb {R}^d$, determined by the family of ODEs (for every $x\in D$)

2.5 $$\begin{aligned} {\left\{ \begin{array}{ll} \partial _t\Phi _t(x)=\xi \big (\Phi _t(x)\big )\quad \text {for every }t\in \mathbb {R}\\ \Phi _0(x)=x. \end{array}\right. } \end{aligned}$$

We consider the family of open sets $\Omega _t:= \Phi _t(\Omega )$ and the corresponding state variables $u_{\Omega _t}$ given by (1.1). We define $\delta u_\Omega$ and $\delta ^2 u_\Omega$ to be the weak solutions in $H^1_0(\Omega )$ to the PDEs

$$\begin{aligned} -\Delta (\delta u_\Omega )={{\,\textrm{div}\,}}((\delta A)\nabla u_\Omega )+\delta f\quad \text {in}\quad \Omega , \qquad \delta u_\Omega \in H^1_0(\Omega ), \end{aligned}$$

and

$$\begin{aligned} -\Delta (\delta ^2 u_\Omega )={{\,\textrm{div}\,}}((\delta A)\nabla (\delta u_\Omega ))+{{\,\textrm{div}\,}}((\delta ^2 A)\nabla u_\Omega )+\delta ^2 f\quad \text {in}\quad \Omega , \qquad \delta ^2 u_\Omega \in H^1_0(\Omega ), \end{aligned}$$

where the matrices $\delta A\in L^\infty (D;\mathbb {R}^{d\times d})$ and $\delta ^2A\in L^\infty (D;\mathbb {R}^{d\times d})$ are given by

2.6 $$\begin{aligned} \begin{aligned} \delta A&:=-D\xi -\nabla \xi +({{\,\textrm{div}\,}}\xi )\text { Id}\,,\\ \delta ^2 A&:= (D\xi )\,(\nabla \xi )+\frac{1}{2}\big (\nabla \xi \big )^2+\frac{1}{2}(D\xi )^2-\frac{1}{2}(\xi \cdot \nabla )\big [\nabla \xi +D\xi \big ]\\&\qquad -\big (\nabla \xi +D\xi \big ){{\,\textrm{div}\,}}\xi +\text { Id}\frac{({{\,\textrm{div}\,}}\xi )^2+\xi \cdot \nabla ({{\,\textrm{div}\,}}\xi )}{2}\,, \end{aligned} \end{aligned}$$

while the variations $\delta f\in L^2(D)$ and $\delta ^2f\in L^2(D)$ of the right-hand side *f* are:

2.7 $$\begin{aligned} \begin{aligned} \delta f&:={{\,\textrm{div}\,}}(f\xi )\,,\\ \delta ^2 f&:= \frac{1}{2} \xi \cdot (D^2f)\xi +\frac{1}{2}\nabla f\cdot D\xi [\xi ]+f\frac{({{\,\textrm{div}\,}}\xi )^2+\xi \cdot \nabla [{{\,\textrm{div}\,}}\,\xi ]}{2}+(\nabla f \cdot \xi ){{\,\textrm{div}\,}}\xi \,. \end{aligned} \end{aligned}$$

Then,

$$\begin{aligned} u_{\Omega _t}\circ \Phi _t = u_\Omega + t (\delta u_\Omega ) + t^2 (\delta ^2 u_\Omega ) + o(t^2)\qquad \text{ in } H^1_0(D). \end{aligned}$$

#### Proof

We set $u_t:=u_{\Omega _t}\circ \Phi _t$. Then, $u_t\in H^1_0(\Omega )$ and $u_{\Omega _t}=u_t\circ \Phi _t^{-1}$.

Moreover, by a change of variables, we have that $u_t$ is satisfies the PDE

$$\begin{aligned} -{{\,\textrm{div}\,}}\big (A_t\nabla u_t\big )=f_t\quad \text {in}\quad \Omega ,\qquad u_t\in H^1_0(\Omega ), \end{aligned}$$

where the matrix $A_t$ and the function $f_t$ are defined as

$$\begin{aligned} f_t:=f(\Phi _t)|\text { det}(D\Phi _t)|\qquad \text {and}\qquad A_t:=(D\Phi _t)^{-1}(D\Phi _t)^{-T}|\text { det}(D\Phi _t)|, \end{aligned}$$

where for a vector field $F:\mathbb {R}^d\rightarrow \mathbb {R}^d$ with components $F_k$, $k=1,\dots ,d$, we denote by

*DF* the matrix with rows $DF_k=(\nabla F_k)^T$. We next compute the second order Taylor expansion of $D\Phi _t$ in $t=0$. By differentiating the equation for the flow $\Phi _t$, we get

$$\begin{aligned} {\left\{ \begin{array}{ll} \partial _t(D\Phi _t)=D\xi (\Phi _t)D\Phi _t\quad \text {for every}\quad t\in \mathbb {R},\\ D\Phi _0=\text { Id}. \end{array}\right. } \end{aligned}$$

Then, taking another derivative in *t*, we get

$$\begin{aligned} \partial _{tt}(D\Phi _t)&=\partial _t\big [D\xi (\Phi _t)D\Phi _t\big ]\\  &=\partial _t\big [D\xi (\Phi _t)\big ]D\Phi _t+D\xi (\Phi _t)\partial _t\big [D\Phi _t\big ]\\  &=\big (\partial _t\Phi _t\cdot \nabla \big )\big [D\xi \big ](\Phi _t)D\Phi _t+D\xi (\Phi _t)D\xi (\Phi _t)D\Phi _t\\  &=\big (\xi (\Phi _t)\cdot \nabla \big )\big [D\xi \big ](\Phi _t)D\Phi _t+D\xi (\Phi _t)D\xi (\Phi _t)D\Phi _t\,, \end{aligned}$$

where for a vector field *F* and a matrix $M=(m_{ij})_{ij}$, we use the notation $(F\cdot \nabla )[M]$ for the matrix with coefficients $F\cdot \nabla m_{ij}$. Finally, taking $t=0$, we get

$$\begin{aligned} \frac{\partial ^2}{\partial t^2}\Big |_{t=0}(D\Phi _t)=(\xi \cdot \nabla )[D\xi ]+(D\xi )^2, \end{aligned}$$

and the Taylor expansion

$$\begin{aligned} D\Phi _t=\text { Id}+tD\xi +\frac{t^2}{2}\Big ((\xi \cdot \nabla )[D\xi ]+(D\xi )^2\Big )+o(t^2). \end{aligned}$$

By the expansions from Remark 2.4, we get

$$\begin{aligned} \begin{aligned} A_t&=\text { Id}+ t (\delta A) + t^2 (\delta ^2 A) + o(t^2)\quad \text {in }L^\infty (D;\mathbb {R}^{d\times d}),\\ f_t&= f + t (\delta f) + t^2 (\delta ^2 f) + o(t^2)\quad \text {in }L^2(D), \end{aligned} \end{aligned}$$

where $\delta A$, $\delta ^2A$, $\delta f$, $\delta ^2f$ are given by (2.6) and (2.7). Thus, the claim follows from Lemmas 2.2 and 2.3. $\square$

### First and Second Variation of $\mathcal {F}$

In the next lemma, we compute the first derivative of the functional $\mathcal {F}$ along inner variations with compact support in *D*.

#### Lemma 2.6

(First variation of $\mathcal {F}$ along inner perturbations) Let *D* be a bounded open set in $\mathbb {R}^d$ and let $f,g,Q\in C^1(D)$. Let $\Omega \subset D$ be open and $\xi \in C^\infty _c(D;\mathbb {R}^d)$ be a vector field with compact support in *D*. Let $\Phi _t$ be the flow of the vector field $\xi$ defined by (2.5) and set $\Omega _t:= \Phi _t(\Omega )$. Then

2.8 $$\begin{aligned} \begin{aligned} \frac{\partial }{\partial t}\bigg |_{t=0}\mathcal {F}({\Omega _t},D)=\,&\int _\Omega \left( \nabla u_\Omega \cdot \nabla v_\Omega +Q\right) {{\,\textrm{div}\,}}\xi \\&+\xi \cdot \nabla Q- \nabla u_\Omega \cdot ((\nabla \xi ) + (D\xi ))\nabla v_\Omega \, dx\\&-\int _\Omega u_\Omega {{\,\textrm{div}\,}}(g\xi ) + v_\Omega {{\,\textrm{div}\,}}(f\xi )\, dx. \end{aligned} \end{aligned}$$

Moreover, if $\partial \Omega$ is $C^2$-regular in a neighborhood of the support of $\xi$, then

2.9 $$\begin{aligned} \frac{\partial }{\partial t}\bigg |_{t=0}\mathcal {F}({\Omega _t},D) = \int _{\partial \Omega } (\nu \cdot \xi )\big (Q-|\nabla u_\Omega ||\nabla v_\Omega |\big )\, d\mathcal {H}^{d-1}, \end{aligned}$$

where $\nu$ is the outer unit normal to $\partial \Omega$.

#### Proof

By applying Lemma 2.5 to $u_t=u_{\Omega _t}\circ \Phi _t$ and $v_t=v_{\Omega _t}\circ \Phi _t,$ we get that

$$\begin{aligned} u_t=u_\Omega +t(\delta u_\Omega )+o(t)\quad \text {and}\quad v_t=v_\Omega +t(\delta v_\Omega )+o(t)\quad \text{ in }\quad H^1_0(D), \end{aligned}$$

where $\delta u_\Omega$ and $\delta v_\Omega$ are the solutions to

2.10 $$\begin{aligned} \begin{aligned} -\Delta (\delta u_\Omega )&={{\,\textrm{div}\,}}((\delta A)\nabla u_{\Omega })+\delta f\quad \text {in }\Omega \ ,\qquad \delta u_\Omega \in H^1_0(\Omega )\,,\\ -\Delta (\delta v_\Omega )&={{\,\textrm{div}\,}}((\delta A)\nabla v_{\Omega })+\delta g \quad \text {in }\Omega \ ,\qquad \delta v_\Omega \in H^1_0(\Omega )\,, \end{aligned} \end{aligned}$$

with $\delta f:={{\,\textrm{div}\,}}(f\xi )$, $\delta g:={{\,\textrm{div}\,}}(g\xi )$, and $\delta A$ as in Lemma 2.5. Therefore, setting

$$\begin{aligned} f_t:=f(\Phi _t)|\text { det}(D\Phi _t)|\,\quad g_t:=g(\Phi _t)|\text { det}(D\Phi _t)|\, \\ Q_t:=Q(\Phi _t)|\text { det}(D\Phi _t)| \quad \text {and}\quad A_t:=(D\Phi _t)^{-1}(D\Phi _t)^{-T}|\text { det}(D\Phi _t)|, \end{aligned}$$

we get

$$\begin{aligned} \mathcal {F}({\Omega _t},D)&=\,\int _{\Omega _t}\Big (\nabla u_{\Omega _t}\cdot \nabla v_{\Omega _t}\,-g u_{\Omega _t}-fv_{\Omega _t}+Q\Big )\,dy\\&=\int _\Omega \Big (\nabla u_t\cdot A_t \nabla v_t-g_t u_t-f_t v_t +Q_t\Big )\,dx\\&=\mathcal {F}(\Omega ,D) + t \int _{\Omega }\Big (\nabla (\delta u_\Omega ) \cdot \nabla v_{\Omega } + \nabla u_{\Omega } \cdot \nabla (\delta v_\Omega )-g(\delta u_\Omega )-f(\delta v_\Omega )\Big )\,dx\\&\quad +t\int _\Omega \Big (\nabla u_\Omega \cdot (\delta A)\nabla v_\Omega -u_\Omega (\delta g)-v_\Omega (\delta f)+(Q{{\,\textrm{div}\,}}\xi + \nabla Q \cdot \xi )\Big )\,dx+o(t)\\&=\mathcal {F}(\Omega ,D) + t \int _\Omega \Big (\nabla u_\Omega \cdot (\delta A)\nabla v_{\Omega }-u_\Omega (\delta g) -v_{\Omega } (\delta f) +{{\,\textrm{div}\,}}(Q\xi )\Big )\,dx + o(t) \end{aligned}$$

where in the first equality we applied the change of variables $y=\Phi _t(x)$ and in the last one we use the equations $-\Delta u_\Omega = f$ and $-\Delta v_\Omega =g$ in $\Omega$. Substituting with the expression for $\delta A$ from (2.6), we obtain (2.8).

Suppose now that $\partial \Omega$ is $C^2$-smooth. Since in $\Omega$ we have the identity

$$\begin{aligned}&\left( \nabla u_\Omega \cdot \nabla v_\Omega \right) {{\,\textrm{div}\,}}\xi -\nabla u_\Omega \cdot ((\nabla \xi ) + (D\xi ))\nabla v_\Omega \\&\quad = {{\,\textrm{div}\,}}\Big (\xi (\nabla u_\Omega \cdot \nabla v_\Omega )-(\nabla u_\Omega \cdot \xi )\nabla v_\Omega - (\nabla v_\Omega \cdot \xi )\nabla u_\Omega \Big ) \\&\qquad +(\nabla v_\Omega \cdot \xi )\Delta u_\Omega + (\nabla u \cdot \xi )\Delta v_\Omega , \end{aligned}$$

by integrating by parts we get

2.11 $$\begin{aligned} \begin{aligned}&\delta \mathcal {F}(\Omega , D)[\xi ] =\\  &=\int _\Omega {{\,\text {div}\,}}\Big (\xi \big ((\nabla u_\Omega \cdot \nabla v_\Omega )+Q\big )-(\nabla u_\Omega \cdot \xi )\nabla v_\Omega - (\nabla v_\Omega \cdot \xi )\nabla u_\Omega \Big )\,dx\\  &\quad -\int _\Omega \Big ((\nabla u_\Omega \cdot \xi )g + u_\Omega {{\,\text {div}\,}}(g\xi ) + (\nabla v_\Omega \cdot \xi )f +v_\Omega {{\,\text {div}\,}}(f\xi )\Big )\, dx\\  &=\int _{\partial \Omega } \Big ((\nu \cdot \xi )((\nabla u \cdot \nabla v)+Q)-(\nabla u \cdot \xi )(\nu \cdot \nabla v) - (\nabla v \cdot \xi )(\nu \cdot \nabla u)\Big )\,d\mathcal {H}^{d-1}. \end{aligned} \end{aligned}$$

Since $u_\Omega$ and $v_\Omega$ are positive in $\Omega$ and vanish on $\partial \Omega$, we have that

$$\begin{aligned} \nabla u_\Omega = -\nu |\nabla u_\Omega |\quad \text {and}\quad \nabla v_\Omega = -\nu |\nabla v_\Omega |\quad \text {on}\quad \partial \Omega , \end{aligned}$$

and so

$$\begin{aligned} \delta \mathcal {F}(\Omega , D)[\xi ] =&\int _{\partial \Omega } (\nu \cdot \xi )(|\nabla u_\Omega ||\nabla v_\Omega |+Q)-|\nabla u_\Omega | (\nu \cdot \xi )|\nabla v_\Omega |\\&- |\nabla v_\Omega |(\nu \cdot \xi )|\nabla u_\Omega |\,d\mathcal {H}^{d-1}\\ =&\int _{\partial \Omega } (\nu \cdot \xi )(Q-|\nabla u_\Omega ||\nabla v_\Omega |)\, d\mathcal {H}^{d-1}, \end{aligned}$$

which concludes the proof. $\square$

#### Remark 2.7

(First variation and stationary domains) Given a bounded open set *D*, functions *f*, *g* and *Q* on *D*, and the functional $\mathcal {F}$ defined in (1.7), we will use the notation $\delta \mathcal {F}(\Omega ,D)[\xi ]$ for the first variation of $\mathcal {F}$ at $\Omega$ along a smooth compactly suppported vector field $\xi \in C^\infty _c(D;\mathbb {R}^d)$. Precisely, we set

$$\begin{aligned} \begin{aligned} \delta \mathcal {F}(\Omega ,D)[\xi ]&:=\, \int _\Omega \left( \nabla u_\Omega \cdot \nabla v_\Omega + Q \right) {{\,\textrm{div}\,}}\xi + \xi \cdot \nabla Q- \nabla u_\Omega \cdot ((\nabla \xi ) + (D\xi ))\nabla v_\Omega \, dx\\&\quad -\int _\Omega u_\Omega {{\,\textrm{div}\,}}(g\xi ) + v_\Omega {{\,\textrm{div}\,}}(f\xi )\, dx. \end{aligned} \end{aligned}$$

We will say that an open set $\Omega \subset D$ is stationary (or a critical point) for $\mathcal {F}$ in *D* if

$$\begin{aligned} \delta \mathcal {F}(\Omega ,D)[\xi ]=0\quad \text {for every}\quad \xi \in C^\infty _c(D;\mathbb {R}^d). \end{aligned}$$

By (2.9), if $\Omega$ is stationary in *D* and the boundary $\partial \Omega \cap D$ is smooth, then

$$\begin{aligned} |\nabla u_\Omega ||\nabla v_\Omega |=Q\quad \text {on}\quad \partial \Omega \cap D. \end{aligned}$$

Finally, we notice that, by Lemma 2.5, any minimizer of (1.3) is stationary for $\mathcal {F}$.

#### Proposition 2.8

(Second variation of $\mathcal {F}$ along inner perturbations) Let $D\subset \mathbb {R}^d$ be a bounded open set in $\mathbb {R}^d$ and let $f,g,Q\in C^2(D)$. Let $\Omega \subset D$ be an open set and $\xi \in C^\infty _c(D;\mathbb {R}^d)$ be a smooth vector field with compact support. Let $\Phi _t$ be the flow of the vector field $\xi$ defined by (2.5) and set $\Omega _t:= \Phi _t(\Omega )$. Then

2.12 $$\begin{aligned} \begin{aligned} \frac{1}{2}\frac{\partial ^2}{\partial t^2}\bigg |_{t=0}\mathcal {F}({\Omega _t},D)&=\, \int _\Omega \Big (\nabla u_\Omega \cdot (\delta ^2 A)\nabla v_\Omega -\nabla (\delta u_\Omega )\cdot \nabla (\delta v_\Omega )\\&\quad - (\delta ^2 f) v_\Omega - (\delta ^2 g) u_\Omega +\delta ^2Q\Big ) dx\,, \end{aligned} \end{aligned}$$

where $\delta ^2 A,\delta ^2 f$, $\delta ^2 g$, $\delta u_\Omega$ and $\delta v_\Omega$ are the ones defined in Lemma 2.5 and where

$$\begin{aligned} \delta ^2Q:=(\xi \cdot \nabla Q){{\,\textrm{div}\,}}\xi +\frac{1}{2}\xi \cdot D^2 Q \xi +\frac{1}{2}Q({{\,\textrm{div}\,}}\xi )^2+\frac{1}{2}Q\xi \cdot \nabla ({{\,\textrm{div}\,}}\xi ). \end{aligned}$$

#### Proof

By applying Lemma 2.5 to $u_t:=u_{\Omega _t}\circ \Phi _t$ and $v_t:=v_{\Omega _t}\circ \Phi _t$, we get that

$$\begin{aligned}  &   u_t = u_{\Omega } + t (\delta u_\Omega ) + t^2 (\delta ^2 u_\Omega )+o(t^2),\\  &   \quad v_t = v_{\Omega } + t (\delta v_\Omega ) + t^2 (\delta ^2 v_\Omega )+o(t^2) \quad \text{ in }\quad H^1_0(\Omega ). \end{aligned}$$

with $\delta u_\Omega ,\delta v_\Omega$ satisfying (2.10) and $\delta ^2 u_\Omega ,\delta ^2 v_\Omega \in H^1_0(\Omega )$ such that

$$\begin{aligned} -\Delta (\delta ^2 u_\Omega )&={{\,\textrm{div}\,}}((\delta A)\nabla (\delta u_\Omega ))+{{\,\textrm{div}\,}}((\delta ^2 A)\nabla u_\Omega )+\delta ^2 f\quad \text {in }\Omega \ ,\qquad \delta ^2u_\Omega \in H^1_0(\Omega )\,,\\ -\Delta (\delta ^2 v_\Omega )&={{\,\textrm{div}\,}}((\delta A)\nabla (\delta v_\Omega ))+{{\,\textrm{div}\,}}((\delta ^2 A)\nabla v_\Omega )+\delta ^2 g\quad \text {in }\Omega \ ,\qquad \delta ^2v_\Omega \in H^1_0(\Omega )\,. \end{aligned}$$

Thus, by computing the second order (in *t*) Taylor expansion of

$$\begin{aligned} \mathcal {F}({\Omega _t},D) =\, \int _{\Omega _t}\Big (\nabla u_{t}\cdot A_t \nabla v_t-g_t u_{t} -f_t v_t +Q_t\Big )\, dx\,, \end{aligned}$$

we get that

$$\begin{aligned}&\frac{1}{2} \frac{\partial ^2}{\partial t^2}\bigg |_{t=0}\mathcal {F}({\Omega _t},D)=\\  &= \int _{\Omega } \nabla (\delta ^2 u_\Omega )\cdot \nabla v_\Omega +\nabla u_\Omega \cdot (\delta ^2 A)\nabla v_\Omega +\nabla u_\Omega \cdot \nabla (\delta ^2 v_\Omega )\, dx\\  &\quad +\int _{\Omega }\nabla (\delta u_\Omega )\cdot \nabla (\delta v_\Omega )+\nabla (\delta u_\Omega )\cdot (\delta A)\nabla v_\Omega +\nabla u_\Omega \cdot (\delta A)\nabla (\delta v_\Omega )\,dx\\  &\quad -\int _{\Omega }f(\delta ^2 v)+(\delta f)(\delta v_\Omega )+(\delta ^2 f)v_\Omega +g(\delta ^2 u_\Omega ) +(\delta g)(\delta u_\Omega )+(\delta ^2 g)u_\Omega \,dx\\  &\quad +\int _\Omega \delta ^2Q\,dx. \end{aligned}$$

Thus, we only have to show that we can write the above expression as in (2.12). By using $\delta v_\Omega$ as a test function in the equation for $\delta u_\Omega$ and vice versa, we obtain

$$\begin{aligned} \int _{\Omega }\nabla (\delta v_\Omega )\cdot (\delta A)\nabla u_\Omega \,dx-\int _\Omega (\delta v_\Omega ) (\delta f)\,dx=-\int _{\Omega } \nabla (\delta u_\Omega )\cdot \nabla (\delta v_\Omega )\,dx \\ \int _{\Omega }\nabla (\delta u_\Omega )\cdot (\delta A)\nabla v_\Omega \,dx-\int _\Omega (\delta u_\Omega )(\delta g)\,dx=-\int _{\Omega } \nabla (\delta u_\Omega )\cdot \nabla (\delta v_\Omega )\,dx. \end{aligned}$$

Then, by testing the equations for $u_\Omega$ and $v_\Omega$ respectively with $\delta ^2v_\Omega$ and $\delta ^2u_\Omega$, we get

$$\begin{aligned} \int _\Omega \nabla (\delta ^2 u_\Omega )\cdot \nabla v_\Omega \,dx=\int _\Omega f\,\delta ^2 v\,dx\quad \text {and}\quad \int _{\Omega } \nabla (\delta ^2 v_\Omega )\cdot \nabla u_\Omega \,dx=\int _\Omega g\,\delta ^2 u_\Omega \,dx. \end{aligned}$$

Using this identities in the expression of the second derivative, we get precisely (2.12). $\square$

#### Remark 2.9

(Second variation and stable critical domains) Given a bounded open set *D*, functions *f*, *g* and *Q* on *D*, and the functional $\mathcal {F}$ from (1.7), we will indicate by $\delta ^2 \mathcal {F}(\Omega ,D)[\xi ]$ the second variation of $\mathcal {F}$ at $\Omega$ along a smooth compactly supported vector field $\xi \in C^\infty _c(D;\mathbb {R}^d)$. Precisely, we set

$$\begin{aligned} \delta ^2 \mathcal {F}(\Omega ,D)[\xi ]:=&\int _\Omega \nabla u_\Omega \cdot (\delta ^2 A)\nabla v_\Omega -\nabla (\delta u_\Omega )\cdot \nabla (\delta v_\Omega )\\&- (\delta ^2 f) v_\Omega - (\delta ^2 g) u_\Omega +\delta ^2Q\,dx\,. \end{aligned}$$

In particular, we notice that for every open set $\Omega$, we have

$$\begin{aligned} \mathcal {F}({\Omega _t},D)=\mathcal {F}(\Omega ,D)+t\delta \mathcal {F}(\Omega ,D)[\xi ]+t^2 \delta ^2\mathcal {F}(\Omega ,D)[\xi ]+o(t^2), \end{aligned}$$

where $\Omega _t=\Phi _t(\Omega )$ (with $\Phi _t$ the flow associated to the vector field $\xi$) and $\delta \mathcal {F}(\Omega ,D)[\xi ]$ is the first variation (2.11).

We will say that an open set $\Omega \subset D$ is a* stable critical point* for $\mathcal {F}$ in *D* if

$$\begin{aligned} \delta \mathcal {F}(\Omega ,D)[\xi ]=0\quad \text {and}\quad \delta ^2 \mathcal {F}(\Omega ,D)[\xi ]\ge 0\quad \text {for every}\quad \xi \in C^\infty _c(D;\mathbb {R}^d). \end{aligned}$$

By Lemmas 2.5 and 2.8, any minimizer of (1.3) is a stable critical point for $\mathcal {F}$.

## Lipschitz Regularity and Non-degeneracy of the State Functions

In this section we study the regularity of the state functions $u_\Omega$ and $v_\Omega$ on an optimal domain $\Omega$, as well as their behavior close to the free boundary $\partial \Omega$. Consequently, we prove that the set $\Omega$ satisfies some density estimates.

### Assumptions on *D*, *f*, *g*, and *Q*

Throughout this subsection we will assume that:

- *D* is a open subset of $\mathbb {R}^d$, with $d\ge 2$;
- $f,g\in L^\infty (D)$ are two functions such that
  $$\begin{aligned} \Vert f\Vert _{L^\infty }+\Vert g\Vert _{L^\infty }\le M\quad \text {and}\quad 0\le C_1g\le f\le C_2g\quad \text {on }D, \end{aligned}$$
  for some positive constants $M,C_1,C_2>0$.
- $Q\in L^\infty (D)$ is such that
  $$\begin{aligned} 0<c_Q\le Q\le C_Q\quad \text {on}\quad D. \end{aligned}$$

### Inwards and Outwards Minimality Conditions

We will use the following notation. Given a set $A\subset \mathbb {R}^d$, and functions $f\in L^2(A)$ and $\varphi \in H^1(A)$, we set

$$\begin{aligned} E_f(\varphi ,A):=\frac{1}{2}\int _A|\nabla \varphi |^2\,dx-\int _Af(x)\varphi \,dx. \end{aligned}$$

#### Proposition 3.1

Let $D\subset \mathbb {R}^d$ and $f,g,Q\in L^\infty (D)$ be as in Sect. 3.1 and let $\Omega \subset D$ be an open set that minimizes (1.3) in *D*. Then, the solution $u_\Omega$ to (1.1) has the following properties.

(i) Outwards minimality. For every open set $\widetilde{\Omega }\subset D$ such that $\Omega \subset \widetilde{\Omega }$ we have
  $$\begin{aligned} E_f(u,D) + \frac{C_2C_Q}{2}|\Omega |\le E_f(\phi ,D)+\frac{C_2C_Q}{2}|\widetilde{\Omega }|\qquad \text{ for } \text{ every } \phi \in H^1_0(\widetilde{\Omega }). \end{aligned}$$
  In particular, for every $B_r(x_0)\subset D$, we have
  3.1 $$\begin{aligned} E_f(u,B_r(x_0))\le E_f(\phi ,B_r(x_0)) + \frac{C_2C_Q}{2}\omega _d r^d, \end{aligned}$$
  for every $\phi \in H^1(B_r(x_0))$ such that $\phi -u_\Omega \in H^1_0(B_r(x_0))$.
(ii) Inwards minimality. For every open set $\omega \subset \Omega$ we have
  3.2 $$\begin{aligned} E_f(u,D) + \frac{C_1c_Q}{2}|\Omega |\le E_f(\phi ,D) + \frac{C_1c_Q}{2}|\omega |\qquad \text{ for } \text{ every } \phi \in H^1_0(\omega ).\nonumber \\ \end{aligned}$$

#### Proof

Suppose that the open set $\widetilde{\Omega }\subset D$ contains $\Omega$. Then, the optimality of $\Omega$ implies that

$$\begin{aligned} \int _\Omega \Big (-g(x)u_\Omega +Q(x)\Big )\,dx\le \int _{\widetilde{\Omega }}\Big (-g(x)u_{\widetilde{\Omega }}+Q(x)\Big )\,dx, \end{aligned}$$

which can be written as

$$\begin{aligned} \int _D g(x)\big (u_{\widetilde{\Omega }}-u_\Omega \big )\,dx\le \int _{\widetilde{\Omega }\setminus \Omega }Q(x)\,dx. \end{aligned}$$

Now, the positivity of *f* implies that $u_{\widetilde{\Omega }}\ge u_\Omega$ on *D*, so we get

$$\begin{aligned} \frac{1}{C_2}\int _D f(x)\big (u_{\widetilde{\Omega }}-u_\Omega \big )\,dx\le \int _D g(x)\big (u_{\widetilde{\Omega }}-u_\Omega \big )\,dx\le \int _{\widetilde{\Omega }\setminus \Omega }Q(x)\,dx\le C_Q|\widetilde{\Omega }\setminus \Omega |, \end{aligned}$$

which after rearranging the terms gives

$$\begin{aligned} -\frac{1}{2}\int _\Omega f(x)u_\Omega +\frac{C_2C_Q}{2}|\Omega |\le -\frac{1}{2}\int _{\widetilde{\Omega }}f(x)u_{\widetilde{\Omega }}+\frac{C_2C_Q}{2}|\widetilde{\Omega }|, \end{aligned}$$

which after an integration by parts on $\widetilde{\Omega }$ and $\Omega$, reads as

$$\begin{aligned} E_f(u_\Omega ,D)+\frac{C_2C_Q}{2}|\Omega |\le E_f(u_{\widetilde{\Omega }},D)+\frac{C_2C_Q}{2}|\widetilde{\Omega }|. \end{aligned}$$

Finally, since $u_{\widetilde{\Omega }}$ minimizes the energy $E_f(\cdot ,D )$ among all functions in $H^1_0(\widetilde{\Omega })$, we obtain (i).

The proof of (ii) is similar. Let $\omega \subset \Omega$ and let $u_\omega$ be the associated state function. Then, using the optimality of $\Omega$ and the bounds on *f*, *g* and *Q*, we get

$$\begin{aligned} c_Q\big (|\Omega |-|\omega |\big )&\le \int _{\Omega \setminus \omega }Q(x)\,dx\le \int _D g\big (u_\Omega -u_\omega \big )\,dx\\&\le \frac{1}{C_1}\int _D f\big (u_\Omega -u_\omega \big )\,dx=\frac{2}{C_1}\Big (E_f(u_\omega ,D)-E_f(u_\Omega ,D)\Big ), \end{aligned}$$

which implies (ii) since $u_\omega$ minimizes $E_f(\cdot ,D )$ in $H^1_0(\omega )$. $\square$

#### Remark 3.2

The state variable $$v_\Omega $$ satisfies analogous inwards/outwards minimality conditions for the functional $$E_g(\cdot ,D)$$, where the constants $$C_1$$ and $$C_2$$ are replaced by $$1/C_2$$ and $$ 1/C_1$$.

### Lipschitz Continuity and Non-degeneracy

As a consequence of Proposition 3.1 we obtain the Lipschitz continuity and the non-degeneracy of $u_\Omega$ (and of $v_\Omega$).

#### Corollary 3.3

Let $D\subset \mathbb {R}^d$ and $f,g,Q\in L^\infty (D)$ be as in Sect. 3.1. If $\Omega \subset D$ is optimal for (1.3), then the state functions $u_\Omega$ and $v_\Omega$ are locally Lipschitz in *D* with Lipschitz constants depending on *d*, $C_1$, $C_2$, *M* and $C_Q$.

#### Proof

By Proposition 3.1 (i), $u_\Omega$ satisfies (3.1) for any $B_r(x_0)\subset D$, so $u_\Omega$ is an almost-minimizer in the sense of [8, Definition 3.1]. Thus, by [8, Theorem 3.3], $u_\Omega$ is locally Lipschitz continuous in *D* with Lipschitz constant depending on *d* and the bounds from above on $\left\| {f} \right\| _{L^\infty }$ and $C_2C_Q$. $\square$

#### Lemma 3.4

Let $D\subset \mathbb {R}^d$ and $f,g,Q\in L^\infty (D)$ be as in Sect. 3.1 and let $\Omega \subset D$ be an optimal set for (1.3) and $u_\Omega$ be the associated state function. Then, there are constants $C_0, r_0>0$, depending on *d*, $C_1$, $c_Q$ and *M*, such that the following implication holds

$$\begin{aligned} \Big (\,\left\| {u_\Omega } \right\| _{L^\infty (B_{r}(x_0))}\leqq C_0 r\,\Big ) \Rightarrow \Big (\, u_\Omega \equiv 0 \quad \text { in } B_{{r}/2}(x_0)\,\Big ), \end{aligned}$$

for every $x_0 \in \overline{\Omega }\cap D$ and every $r \in (0,r_0]$. In other words, if $x_0\in {\overline{\Omega }}$ and $B_r(x_0)\subset D$, then

$$\begin{aligned} \sup _{B_r(x_0)} u_\Omega \ge C_0r. \end{aligned}$$

#### Proof

By Proposition 3.1 (ii), we have that for every $\omega \subset \Omega$

$$\begin{aligned} E_f(u_\Omega ,D)+\frac{c_QC_1}{2}|\Omega |\le E_f(u_\omega ,D)+\frac{c_QC_1}{2}|\omega |. \end{aligned}$$

Thus, the claim follows by [2, Lemma 4.4], [9, Lemma 3.3], or [20, Lemma 2.8]. $\square$

### Density Estimates on the Boundary of $\Omega$

An a consequence of the Lipschitz continuity and the non-degeneracy of $u_\Omega$ and $v_\Omega$, we obtain density estimates for the optimal set $\Omega$.

#### Proposition 3.5

Let $D\subset \mathbb {R}^d$ and $f,g,Q\in L^\infty (D)$ be as in Sect. 3.1. Then, there are ${\varepsilon }_0, r_0>0$ (depending on $C_1$, $C_2$, *M*, *d*, $c_Q$, $C_Q$) such that for every set $\Omega \subset D$ optimal for (1.3)

3.3 $$\begin{aligned} {\varepsilon }_0|B_r|\leqq |B_r(x_0)\cap \Omega | \leqq (1-{\varepsilon }_0)|B_r|. \end{aligned}$$

for every ball $B_r(x_0)\subset D$ of radius $r\le r_0$ centered on $\partial \Omega$.

#### Proof

Assume that $x_0=0\in \partial \Omega$. The lower estimate is an immediate consequence of the Lipschitz continuity (Lemma 3.3) and the non-degeneracy (Lemma 3.3) of $u_\Omega$. The upper bound can be obtained as in [2]. Precisely, consider the solution *h* to

$$\begin{aligned} -\Delta h =M\quad \text{ in } B_r\,\qquad h = u_\Omega \quad \text{ on } D\setminus B_r. \end{aligned}$$

Since $\Delta u_\Omega +f\ge 0$ in $\mathbb {R}^d$, we get that $-\Delta (h-u_\Omega )\ge M-f\geqq 0$ in $B_r$. In particular, we have that $u_\Omega \leqq h$ and $\{u_\Omega>0\}\subset \{h>0\}$ in $B_r$. Thus, testing the optimality (3.1) of $u_\Omega$ with *h*,

$$\begin{aligned} \frac{C_2C_Q}{2}|B_r \cap \{u_\Omega =0\}|&\ge E_f(u_\Omega ,B_r) - E_f(h,B_r) \\  &= \frac{1}{2} \int _{B_r}|\nabla (u_\Omega -h)|^2\,dx\\  &\quad + \int _{B_r} \Big (\nabla h\cdot \nabla (u_\Omega -h) - f(u_\Omega -h)\Big )\, dx\\  &\ge \frac{1}{2} \int _{B_r}|\nabla (u_\Omega -h)|^2\, dx. \end{aligned}$$

By the Poincaré and Cauchy-Schwarz inequalities, we have

$$\begin{aligned} \int _{B_r}|\nabla (u_\Omega -h)|^2\, dx \ge \frac{C_d}{|B_r|}\left( \frac{1}{r} \int _{B_r}(h-u_\Omega )\,dx\right) ^2, \end{aligned}$$

so in order to prove the upper bound in (3.3), we only need to show that $\frac{1}{r^{d+1}} \int _{B_{r}}(h-u_\Omega )\,dx$ is bounded from below by a positive constant. Notice that, by the non-degeneracy of $u_\Omega$, we have

$$\begin{aligned} \widetilde{C}r \leqq \sup _{B_{r/2}} u_\Omega \leqq \sup _{B_{r/2}}h. \end{aligned}$$

On the other hand, since $h(x)+\frac{M}{2d}|x|^2$ is harmonic in $B_R$, the Harnack inequality in $B_r$ implies

$$\begin{aligned} \widetilde{C}r \le \sup _{B_{r/2}}h\le C_d\big (h(x)+Mr^2\big )\quad \text {for every}\quad x\in B_{{r}/2}. \end{aligned}$$

Thus, by taking $r_0$ such that $2C_dr_0M\le {\widetilde{C}}$, we get that $h \geqq C_d\widetilde{C} r = \overline{C}r$ in $B_{{r}/2}.$ On the other hand, if *L* is the Lipschitz constant of $u_\Omega$, then for any ${\varepsilon }\in (0,1)$, $u_\Omega \leqq L {\varepsilon }r$ in $B_{{\varepsilon }r}$. Then

$$\begin{aligned} \int _{B_r}(h-u_\Omega )\,dx\ge \int _{B_{{\varepsilon }r}} (h-u_\Omega )\,dx\ge ({\bar{C}} r-L{\varepsilon }r)|B_{{\varepsilon }r}|, \end{aligned}$$

which concludes the proof after choosing ${\varepsilon }\le 1/2$ small enough. $\square$

### An Estimate on the Level Sets of $u_\Omega$

We conclude the section with this auxiliary result that will play a crucial role in Sect. 4.3 in the proof of the existence of homogeneous blow-up limits.

#### Lemma 3.6

Let $D\subset \mathbb {R}^d$ and $f,g,Q\in L^\infty (D)$ be as in Sect. 3.1; let $\Omega$ be a solution to (1.3) and let $u_\Omega$ be the associated state function. Then, there are constants $C>0$ and $r_0>0$, depending only on $d,M,C_1,C_2,c_Q,C_Q$, such that

3.4 $$\begin{aligned} |\{0<u<rt\}\cap B_r(x_0)|\leqq C t |B_r|, \end{aligned}$$

for every $B_r(x_0)\subset D$ centered on $\partial \Omega$ and every $t\in (0,1)$.

#### Proof

The estimate is contained in the proof of [11, Theorem 1.10]; we sketch the idea for the sake of completeness. Let $x_0 = 0 \in \partial \Omega$ and $t>0$. We fix a function $\eta \in C^\infty _c(B_{2r})$ such that $0\le \eta \le 1$ in $B_{2r}$ and $\eta \equiv 1$ in $B_r$, and we use the competitor

$$\begin{aligned} \phi = \eta (u_\Omega - rt)^+ + (1-\eta ) u_\Omega . \end{aligned}$$

to test the optimality condition (3.2) in $B_{2r}$. Since

$$\begin{aligned} \phi = u_\Omega - rt \eta \quad \text { in } \{u_\Omega >rt \}\qquad \text{ and }\qquad \phi = (1-\eta ) u_\Omega \quad \text { in } \{0\leqq u_\Omega \leqq rt\}, \end{aligned}$$

we get

$$\begin{aligned} E_f(\phi ,B_{2r})&= \int _{\{u_\Omega>rt\}\cap B_{2r}}\bigg (\frac{1}{2}|\nabla u_\Omega |^2+ \frac{(rt)^2}{2}|\nabla \eta |^2-rt\nabla u_\Omega \cdot \nabla \eta - fu_\Omega + frt\eta \bigg )\,dx\\  &\quad + \int _{\{0\leqq u_\Omega \leqq rt\}\cap B_{2r}}\bigg (\frac{(1-\eta )^2}{2}|\nabla u_\Omega |^2+ \frac{u_\Omega ^2}{2}|\nabla \eta |^2-(1-\eta )u_\Omega \nabla u_\Omega \cdot \nabla \eta - f u_\Omega + f u_\Omega \eta \bigg )\,dx\\  &\leqq \, E_f(u_\Omega ,B_{2r}) - rt \int _{\{u_\Omega >rt\}\cap B_{2r}} \nabla u_\Omega \cdot \nabla \eta \,dx + \int _{B_{2r}}\bigg (\frac{(rt)^2}{2}|\nabla \eta |^2+ frt\eta \bigg )\,dx\\  &\quad + \int _{\{0\leqq u_\Omega \leqq rt\}\cap B_{2r}}\bigg (\frac{(1-\eta )^2-1}{2}|\nabla u_\Omega |^2-(1-\eta )u_\Omega \nabla u_\Omega \cdot \nabla \eta \bigg )\,dx\\  &\leqq \, E_f(u_\Omega ,B_{2r}) + C_d\Big (t\Vert \nabla u_\Omega \Vert _{L^{\infty }} +rt\left\| {f} \right\| _{L^\infty } +t^2\Big )|B_r|. \end{aligned}$$

Thus (3.2) implies

$$\begin{aligned} \frac{C_1c_Q}{2}|\{0<u_\Omega \le rt\}\cap B_r|&\le \frac{C_1c_Q}{2}\Big (|\{u_\Omega>0\}\cap B_{2r}|-|\{\varphi >0\}\cap B_{2r}|\Big )\\&\le E_f(\phi ,D)-E_f(u_\Omega ,D)\\&\le C_d\Big (t\Vert \nabla u\Vert _{L^{\infty }} +rt\left\| {f} \right\| _{L^\infty } +t^2\Big )|B_r|. \end{aligned}$$

Therefore, we get

$$\begin{aligned} \frac{C_1c_Q}{2}|B_{r} \cap \{0\leqq u\leqq rt\}| \leqq Ct|B_r| \end{aligned}$$

with $C>0$ depending on *d*, $\Vert u_\Omega \Vert _{L^\infty (B_{2r})}$, *M*, $C_1$ and $c_Q$. $\square$

## Compactness and Convergence of Blow-Up Sequences

Take an optimal set $\Omega$ for (1.3) in some $D\subset \mathbb {R}^d$, and consider the corresponding state functions $u=u_\Omega$ and $v=v_\Omega$. For any $x_0 \in \partial \Omega \cap D$ and any sequence $r_k \rightarrow 0^+$, we set

$$\begin{aligned} u_{x_0,r_k}(x):= \frac{1}{r_k}u(x_0 +r_k x),\quad v_{x_0,r_k}(x):= \frac{1}{r_k}v(x_0 +r_k x),\quad \Omega _{x_0,r_k}:= \frac{\Omega - x_0 }{r_k}. \end{aligned}$$

Since *u* and *v* vanish in $x_0$ and are Lipschitz (in a neighborhood of $x_0$), we have that $u_{x_0,r_k}$ and $v_{x_0,r_k}$ vanish in 0 and (for large *k*) are uniformly Lipschitz in any ball $B_R$. Thus, there are functions $u_0,v_0:\mathbb {R}^d\rightarrow \mathbb {R}$ and subsequences of $u_{x_0,r_{k}}$ and $v_{x_0,r_{k}}$ that converge locally uniformly in $\mathbb {R}^d$ respectively to $u_0$ and $v_0$. As usual we say that $u_0$ and $v_0$ are blow-up limits of *u* and *v* in $x_0$; and we recall that they might depend on the sequence $r_{k}$. We notice that the blow-up limits of *u* and *v* will always be taken along the same sequence $r_k\rightarrow 0$.

The main results are Propositions 4.3 and 4.7. In Proposition 4.3 we list the properties of any couple of functions $u_0,v_0$ obtained as blow-up limits of *u*, *v*, while in Proposition 4.7 we show that there is at least one sequence $r_k\rightarrow 0$ that provides blow-up limits which are 1-homogeneous and stationary for the one-phase Alt–Caffarelli functional.

### A General Lemma About the Convergence of Blow-Up Sequences

The construction of the blow-up limit from Proposition 4.7 will require taking three consecutive blow-ups. We give here a general lemma, which we will use several times in this section.

#### Lemma 4.1

Let $B_{2R}$ be a ball in $\mathbb {R}^d$. Let $u_n:{\overline{B}}_{2R}\rightarrow \mathbb {R}$ be a sequence of non-negative Lipschitz functions converging uniformly to a Lipschitz function $u_\infty :{\overline{B}}_{2R}\rightarrow \mathbb {R}$, and suppose that there is a constant $L>0$ such that

$$\begin{aligned} \Vert \nabla u_n\Vert _{L^\infty (B_{2R})}\le L\quad \text {for every}\quad n\ge 1. \end{aligned}$$

Then, the following holds.

(i) Suppose that there is a constant ${\tilde{C}}>0$ such that, for every $n\ge 1$,
  4.1 $$\begin{aligned} \sup _{B_r(x_0)} u_n \ge \tilde{C} r\quad \text {for every}\quad x_0\in B_R\cap \overline{\{u_n>0\}}\quad \text {and every}\quad r\in (0,R).\nonumber \\ \end{aligned}$$
  Then
  4.2 $$\begin{aligned} \sup _{B_r(x_0)} u_\infty \ge \tilde{C} r\quad \text {for every}\quad x_0\in B_R\cap \overline{\{u_\infty >0\}}\quad \text {and every}\quad r\in (0,R),\nonumber \\ \end{aligned}$$
  and
  4.3 $$\begin{aligned} \mathbbm {1}_{\{u_n>0\}}\rightarrow \mathbbm {1}_{\{u_\infty >0\}}\quad \text {pointwise a.e. in }B_R. \end{aligned}$$
(ii) Suppose that there is a constant ${\tilde{M}}>0$ such that, for every $n\ge 1$, we have the bound
  4.4 $$\begin{aligned} \left| \int _{B_{2R}}\nabla u_n\cdot \nabla \varphi \,dx\right| \le \tilde{M} \Vert \varphi \Vert _{L^\infty (B_{2R})}\quad \text {for every }\varphi \in C^{0,1}_c(B_{2R}), \end{aligned}$$
  where $C^{0,1}_c(B_{2R})$ is the space of Lipschitz functions with compact support in $B_{2R}$. Then $u_n$ converges to $u_\infty$ strongly in $H^1(B_R)$.

#### Proof

We first prove (i). Suppose that $x_0\in B_R\cap \overline{\{u_\infty >0\}}$. Then, there is a sequence $x_n\rightarrow x_0$ of points $x_n\in B_R\cap \overline{\{u_\infty >0\}}$. Let $r_n:=r-|x_n-x_0|$. By (4.1), there is a point $y_n\in B_{r_n}(x_n)\subset B_r(x_0)$ such that $u_n(y_n)\ge {\widetilde{C}}r_n$. Thus, by the uniform convergence of $u_n$, we get (4.2). In order to prove (4.3), we first notice that by the pointwise convergence of $u_n$ to $u_\infty$, we have that

$$\begin{aligned} \mathbbm {1}_{\{u_\infty>0\}}(x_0)=1\ \Rightarrow \ \mathbbm {1}_{\{u_n>0\}}(x_0)=1\quad \text{ for } \text{ large } \text{ n }, \end{aligned}$$

so it is sufficient to prove that the set

$$\begin{aligned} S:=\Big \{x_0\in B_{R}\ :\ \mathbbm {1}_{\{u_\infty>0\}}(x_0)=0\ \text{ and } \ \limsup _{n\rightarrow \infty }\mathbbm {1}_{\{u_{n}>0\}}(x_0)=1\Big \}, \end{aligned}$$

is of measure zero. Now, by (4.1), we get that for every $r\in (0,R)$ there is a sequence $y_n\in {\overline{B}}_r(x_0)$ such that $u_n(y_n)\ge \tilde{C}r$. Then, by the uniform convergence of $u_n$, we have that there is $y_\infty \in {\overline{B}}_r(x_0)$ such that $u_\infty (y_\infty )\ge \tilde{C}r$. Then, by the Lipschitz continuity of $u_\infty$, the ball $B_{\tilde{C} r/L}(y_\infty )$ is contained in $\{u_\infty >0\}$. Since *r* is arbitrary, we get that the Lebesgue density of $\{u_\infty >0\}$ in $x_0$ cannot be zero, so the set *S* has zero measure.

We next prove (ii). Since $u_n$ is uniformly bounded in $H^1(B_{2R})$, we have that $u_n$ converges to $u_\infty$ weakly in $H^1(B_{2R})$. Thus, the estimate (4.4) holds also for $u_\infty$, that is

$$\begin{aligned} \left| \int _{B_{2R}}\nabla u_\infty \cdot \nabla \varphi \,dx\right| \le \tilde{M} \Vert \varphi \Vert _{L^\infty (B_{2R})}\quad \text {for every }\varphi \in C^{0,1}_c(B_{2R}). \end{aligned}$$

Choose a function $\varphi \in C^\infty _c(B_{2R})$ such that $\varphi \equiv 1$ in $B_R$. Then,

$$\begin{aligned} \int _{B_R}|\nabla (u_n-u_\infty )|^2\,dx&\le \int _{B_{2R}}|\nabla (\varphi (u_n-u_\infty ))|^2\,dx\\&=\int _{B_{2R}}|\nabla \varphi |^2(u_n-u_\infty )^2\,dx\\&\quad +\int _{B_{2R}}\nabla (u_n-u_\infty )\cdot \nabla \big (\varphi ^2(u_n-u_\infty )\big )\,dx\\&\le \int _{B_{2R}}|\nabla \varphi |^2(u_n-u_\infty )^2\,dx+2\tilde{M}\Vert \varphi ^2(u_n-u_\infty )\Vert _{L^\infty (B_{2R})}. \end{aligned}$$

Since the right-hand side converges to zero, we get the claim. $\square$

Finally, we notice that the above lemma can be applied to the state functions $u_\Omega$ and $v_\Omega$ of an optimal domain. This is a consequence of the following lemma.

#### Lemma 4.2

Let $B_{2R}\subset \mathbb {R}^d$ and $u\in H^1(B_{2R})$ be a non-negative function with the following properties.

- There is a function $f\in L^\infty (B_{2R})$ such that
  $$\begin{aligned} -\Delta u=f\quad \text {in }\Omega _u:=\{u>0\},\qquad u=0\quad \text {on }\partial \Omega _u\cap B_{2R}, \end{aligned}$$
  in the sense that
  $$\begin{aligned} \int _{B_{2R}}\nabla u\cdot \nabla \varphi \,dx=\int _{B_{2R}}\varphi f\,dx\quad \text {for every }\varphi \in H^1_0(B_{2R})\quad \text {with}\quad \varphi =0\quad \text {in }B_{2R}\setminus \Omega _u. \end{aligned}$$
- There is $\Lambda >0$ such that, for every non-negative $\varphi \in H^1_0(B_{2R})$,
  $$\begin{aligned} E_f(u,B_{2R})+\frac{\Lambda }{2}|B_{2R}\cap \{u>0\}|\le E_f(u+\varphi ,B_{2R})+\frac{\Lambda }{2}|B_{2R}\cap \{u+\varphi >0\}|. \end{aligned}$$

Then, for every $\varphi \in C^{0,1}_c(B_{R})$, we have

$$\begin{aligned} \left| \int _{B_{R}}\nabla u\cdot \nabla \varphi \,dx\right| \le C_d\Big (1+\Lambda +R\Vert f\Vert _{L^\infty (B_{2R})}\Big )R^{d-1}\Vert \varphi \Vert _{L^\infty (B_{R})}. \end{aligned}$$

#### Proof

We only sketch the proof and we refer to [38, Chapter 3] for the details. By (a) we have that

$$\begin{aligned} \mu :=\Delta u+|f| \end{aligned}$$

is a positive Radon measure on $B_{2R}$, where

$$\begin{aligned} \int _{B_{2R}}\varphi \,d\mu :=\int _{B_{2R}}\Big (-\nabla u\cdot \nabla \varphi +\varphi |f|\Big )\,dx\qquad \text {for every }\varphi \in C^{0,1}_c(B_{2R}). \end{aligned}$$

By testing the optimality condition in (*b*) with a function $\varphi \in C^{0,1}_c(B_{2R})$, we get

$$\begin{aligned} \int _{B_{2R}}\varphi \,d\mu =\int _{B_{2R}}\Big (-\nabla u\cdot \nabla \varphi +\varphi f\Big )\,dx\le \int _{B_{2R}}|\nabla \varphi |^2\,dx+\Lambda |B_{2R}|, \end{aligned}$$

and choosing $\varphi =R\phi$ with $\phi \equiv 1$ in $B_{R}$, we obtain

$$\begin{aligned} \mu (B_{R})\le \int _{B_{2R}}\phi \,d\mu \le C_d(1+\Lambda )R^{d-1}. \end{aligned}$$

As a consequence, for every $\varphi \in C^{0,1}_c(B_R)$, we have

$$\begin{aligned}  &   \left| \int _{B_{R}}\nabla u\cdot \nabla \varphi \,dx\right| \le \int _{B_R}|\varphi |\,d\mu \\  &   +\int _{B_{R}}|\varphi | |f|\,dx\le C_d\Big (1+\Lambda +R\Vert f\Vert _{L^\infty }\Big )R^{d-1}\Vert \varphi \Vert _{L^\infty (B_R)}. \end{aligned}$$

which concludes the proof. $\square$

### First Blow-Up

In this subsection we list the properties of any couple of blow-ups

$$\begin{aligned} u_0,v_0:\mathbb {R}^d\rightarrow \mathbb {R}\end{aligned}$$

of the state functions $u_\Omega$ and $v_\Omega$ at a boundary point $x_0\in \partial \Omega \cap D$. The qualitative properties (Lipschitz continuity, non-degeneracy, density estimates) of $u_\Omega$ and $v_\Omega$ are conserved under blow-up limits; the stationarity condition also passes to the limit; the main difference is that $u_0$ and $v_0$ are harmonic where they are positive.

#### Proposition 4.3

Let $D\subset \mathbb {R}^d$ be a bounded open set, let $\Omega$ be a solution to (1.3) with *f*, *g*, *Q* as in Theorem 1.2, and let $u:=u_\Omega$ and $v:=v_\Omega$ be the state functions on $\Omega$ defined in (1.1) and (1.6). We consider a point $x_0\in \partial \Omega \cap D$ and blow-up sequences $u_{x_0,r_k},v_{x_0,r_k}$ of *u*, *v* converging locally uniformly in $\mathbb {R}^d$ to blow-up limits $u_0,v_0\in C^{0,1}(\mathbb {R}^d)$. Then

1. taking $C_{1}$ and $C_{2}$ to be the constants from (1.2), we have
  $$\begin{aligned} C_1v_0\le u_0\le C_2v_0\quad \text {on }\mathbb {R}^d; \end{aligned}$$
2. the functions $u_0$ are $v_0$ are harmonic in the open set $\Omega _0:=\{u_0>0\}=\{v_0>0\}$.
3. there are constants ${\varepsilon }_0>0$ and $C>0$ such that
  4.5 $$\begin{aligned} {\varepsilon }_0 |B_r|\le |B_r(x_0)\cap \Omega _0|\le (1-{\varepsilon }_0)|B_r|, \end{aligned}$$
  for every $x_0\in \partial \Omega _0$, $r>0$, and
  4.6 $$\begin{aligned} |\{0<u_0<rt\}\cap B_r(x_0)|\leqq Ct|B_r|, \end{aligned}$$
  for every $$x_0\in \partial \Omega _0, r>0,t>0$$;
4. $0\in \partial \Omega _0$ and there is a constant $C_0 > 0$ such that
  $$\begin{aligned} \sup _{B_r(x_0)} u_0 \ge C_0 r\quad \text {and}\quad \sup _{B_r(x_0)} v \ge C_0 r\quad \text {for every}\quad x_0\in {\overline{\Omega }}_0\quad \text {and every}\quad r>0; \end{aligned}$$
5. there is a constant $\Lambda >0$ depending on $C_1,C_2,C_Q$ and *d* such that, for every $R>0$,
  $$\begin{aligned} \left| \int _{B_{R}}\nabla u_0\cdot \nabla \varphi \,dx\right| +\left| \int _{B_{R}}\nabla v_0\cdot \nabla \varphi \,dx\right| \le \Lambda R^{d-1}\Vert \varphi \Vert _{L^\infty (B_{R})}; \end{aligned}$$
6. for every compactly supported smooth vector field $\xi \in C^\infty _c(\mathbb {R}^d;\mathbb {R}^d)$, we have
  $$\begin{aligned} \int _{\mathbb {R}^d} \Big (- \nabla u_{0} \cdot \big ((\nabla \xi ) + (D\xi )\big )\nabla v_{0}+\big (\nabla u_{0}\cdot \nabla v_{0}+ Q(x_0)\mathbbm {1}_{\Omega _0} \big ){{\,\textrm{div}\,}}\xi \Big )\, dx=0. \end{aligned}$$

#### Proof

For simplicity, we set

$$\begin{aligned} u_k:=u_{x_0,r_k},\ v_k:=v_{x_0,r_k},\ f_k:=f_{x_0,r_k},\ g_k:=g_{x_0,r_k}\ \text { and }\ \Omega _k:= (\Omega -x_0)/r_k, \end{aligned}$$

and we notice that

$$\begin{aligned} -\Delta u_k=r^2_kf_k\quad \text {and}\quad -\Delta v_k=r^2_kg_k\quad \text {in }\Omega _k. \end{aligned}$$

The first two claims are just a consequence of the locally uniform convergence of $u_k$ and $v_k$ to $u_0$ and $v_0$. By Proposition 3.1, Corollary 3.3 and Lemma 3.4, we already know that *u* and *v* fulfill the assumptions of Lemma 4.2. Therefore, (4) and (5) follow from Lemma 4.1, while (6) follows from Lemma 2.6 and Remark 2.7, and the strong $H^1$ convergence of $u_k$ and $v_k$.

We next prove (3). By Proposition 3.5 and rescaling, we know that, for every $k>0$,

$$\begin{aligned} {\varepsilon }_0|B_r|\le |B_r(x_0)\cap \{u_k>0\}|\le (1-{\varepsilon }_0)|B_r|,\quad \text {for }r<r_0/r_k,\ x_0\in \partial \Omega _k, \end{aligned}$$

so, by the strong convergence of $\mathbbm {1}_{\Omega _k}$ to $\mathbbm {1}_{\Omega _0}$ in $L^1_{loc}(\mathbb {R}^d)$ (see Lemma 4.1), we get the density estimate (4.5) for $\Omega _0$. Similarly, by rescaling (3.4) we obtain

$$\begin{aligned} |\{0<u_k<rt\}\cap B_r(x_0)|\leqq C t |B_r| \quad \text{ for }\quad r<\frac{r_0}{r_k},\ x_0\in \partial \Omega _k,\ t>0, \end{aligned}$$

which, passing to the limit as $k\rightarrow \infty$, gives (4.6). $\square$

### Second Blow-Up

Consider blow-up limits $u_{0},v_{0}$ as in the previous subsection and let

$$\begin{aligned} u_{00},v_{00}:\mathbb {R}^d\rightarrow \mathbb {R}. \end{aligned}$$

be blow-up limits of $u_0$ and $v_0$ in zero. Then $u_{00}$ and $v_{00}$ are still blow-up limits of the state functions $u_\Omega$ and $v_\Omega$ at $x_0\in \partial \Omega \cap D$ and Proposition 4.3 still applies. On the other hand, [29, Theorem 1.2] applies to the domain $\Omega _0$ and the function $u_0$, so the Boundary Harnack Principle (see [29, Definition 1.1]) holds on $\Omega _0$; since $u_0$ and $v_0$ are harmonic in $\Omega _0$, we get that the ratio $u_0/v_0$ is Hölder continuous up to the boundary $\partial \Omega _0$. This, in particular means that the second blow-ups $u_{00}$ and $v_{00}$ at any boundary point (and thus in zero) are proportional.

#### Lemma 4.4

Let $u_0,v_0:\mathbb {R}^d\rightarrow \mathbb {R}$ be non-negative Lipschitz functions on $\mathbb {R}^d$ with the same positivity set and let $\Omega _0:=\{u_0>0\}=\{v_0>0\}$. Suppose that $u_0$ and $v_0$ satisfy the conditions (1)-(6) from Proposition 4.3 and let $u_{00},v_{00}:\mathbb {R}^d\rightarrow \mathbb {R}$ be blow-ups of $u_0,v_0$ at zero. Then

1. there is a constant $\lambda \in (C_1,C_2)$ such that $u_{00}=\lambda v_{00}$ on $\mathbb {R}^d$;
2. the function $u_{00}$ is harmonic in the open set $\Omega _{00}:=\{u_{00}>0\}$;
3. there are constants ${\varepsilon }_0>0$ and $C>0$ such that
  $$\begin{aligned} {\varepsilon }_0 |B_r|\leqq |B_r(x_0)\cap \Omega _{00}| \leqq (1-{\varepsilon }_0)|B_r|, \end{aligned}$$
  for every $x_0\in \partial \Omega _{00}$, $r>0$, and
  $$\begin{aligned} |\{0<u_{00}<rt\}\cap B_r(x_0)|\leqq Ct|B_r|, \end{aligned}$$
  for every $$x_0\in \partial \Omega _{00}, r>0,t>0$$;
4. $0\in \partial \Omega _{00}$ and there is a constant $C_0 > 0$ such that
  $$\begin{aligned} \sup _{B_r(x_0)}u_{00} \ge C_0 r\quad \text {for every}\quad x_0\in {\overline{\Omega }}_{00}\quad \text {and every}\quad r>0; \end{aligned}$$
5. there is a constant $\Lambda >0$ such that, for every $R>0$,
  $$\begin{aligned} \left| \int _{B_{R}}\nabla u_{00}\cdot \nabla \varphi \,dx\right| \le \Lambda R^{d-1}\Vert \varphi \Vert _{L^\infty (B_{R})}; \end{aligned}$$
6. for every compactly supported smooth vector field $\xi \in C^\infty _c(\mathbb {R}^d;\mathbb {R}^d)$, we have
  4.7 $$\begin{aligned} \int _{\mathbb {R}^d} \bigg (- \nabla u_{00} \cdot \big ((\nabla \xi ) + (D\xi )\big )\nabla u_{00}+\Big (|\nabla u_{00}|^2+ {\lambda }Q(x_0)\mathbbm {1}_{\Omega _{00}} \Big ){{\,\textrm{div}\,}}\xi \bigg )\, dx=0. \end{aligned}$$

#### Proof

Let $R>0$ be fixed and let $\phi :=u_0$. In order to show that the Boundary Harnack Principle holds on $\Omega _0$, we check that $\Omega _0$ and $\phi$ satisfy the list of assumptions (a)–(g) from [29, Theorem 1.2] in the ball $B_R$:

- by definition of $\Omega _0$, we have $\phi >0$ in $\Omega _0$ and $\phi \equiv 0$ on $B_R\setminus \Omega _0$;
- by hypothesis $\phi$ is Lipschitz continuous in $\mathbb {R}^d$ and so, in $B_R$;
- since $\phi$ is harmonic in $\Omega _0$ and satisfies the condition (4) from Proposition Proposition 4.3, we can apply [38, Lemma 6.8]; thus, there is a constant $\kappa >0$ such that
  $$\begin{aligned} \phi \ge \kappa \,{{\,\textrm{dist}\,}}_{B_R\setminus \Omega _0}\quad \text {in }B_{{R}/{2}}; \end{aligned}$$
- since $\phi \geqq 0$ and $\Delta \phi =0$ in $\Omega _0$, we have that $\Delta \phi \ge 0$ in $\mathbb {R}^d$;
- for every $x_0\in \partial \Omega \cap B_{R}$, we have
  $$\begin{aligned} |B_r(x_0)\setminus \Omega _0|\ge {\varepsilon }_0 |B_r(x_0)|\qquad \text {for every }r\in (0,R-|x_0|); \end{aligned}$$
- for every $x_0\in \partial \Omega _0\cap B_{R}$ and every $r\in (0,R-|x_0|)$, we have
  $$\begin{aligned} \big |\{0<\phi <rt\}\cap B_{r}(x_0)\big |\le C t|B_r|\qquad \text {for every }t>0;. \end{aligned}$$
- by (4) of Proposition 4.3, for every $x_0\in \partial \Omega _0\cap B_{R}$ and every $r\in (0,R-|x_0|)$, we have
  $$\begin{aligned} \sup _{B_r(x_0)}\phi \ge C_0 r. \end{aligned}$$

Therefore, all the assumptions of [29, Theorem 1.2] are fulfilled and so, the ratio

$$\begin{aligned} \frac{u_0}{v_0}:\Omega _0\rightarrow \mathbb {R}, \end{aligned}$$

can be extended to a Hölder continuous function on ${\overline{\Omega }}_0\cap B_{{R}/{2}}$. Thus, the blow-ups $u_{00}$ and $v_{00}$ are proportional and all the other claims follow as in Proposition 4.3. $\square$

### Third Blow-Up

Take the state functions $u_\Omega$ and $v_\Omega$ on an optimal domain $\Omega$. Let

$$\begin{aligned} u_{00},v_{00}:\mathbb {R}^d\rightarrow \mathbb {R}, \end{aligned}$$

be the second blow-up limits of $u_\Omega$ and $v_\Omega$ at a free boundary point $x_0\in \partial \Omega \cap D$. Then, $u_{00}$ and $v_{00}$ are proportional and satisfy the conditions listed in Lemma 4.4. We will show that if we perform a further blow-up in zero, then we obtain functions $u_{000},v_{000}:\mathbb {R}^d\rightarrow \mathbb {R}$ that still satisfy the conditions from Lemma 4.4 but are also 1-homogeneous. Before we give the precise statement, we notice that the stationarity condition (4.7) implies the monotonicity of the associated Weiss’ boundary adjusted energy from [39].

#### Lemma 4.5

(Monotonicity formula) Let $B_R\subset \mathbb {R}^d$, $u \in H^1(B_R)$ be a continuous non-negative function, and let $\Omega :=\{u>0\}$. Suppose that for every smooth compactly supported vector field $\xi \in C^\infty _c(\mathbb {R}^d;\mathbb {R}^d)$, we have:

$$\begin{aligned} \int _\Omega \Big ({{\,\textrm{div}\,}}\xi \left( |\nabla u|^2 + \Lambda \right) - \nabla u \cdot ((\nabla \xi ) + (D\xi ))\nabla u\Big ) \, dx =0. \end{aligned}$$

Then, for every $x_0 \in \partial \Omega , r>0$ the map

$$\begin{aligned} r \mapsto W_\Lambda (u_{x_0,r}):= \int _{B_1}|\nabla u_{x_0,r}|^2\,dx+\Lambda |\{u_{x_0,r}>0\}\cap B_1|- \int _{\partial B_1} u_{x_0,r}^2 \, d\mathcal {H}^{d-1}, \end{aligned}$$

is non-decreasing in $(0,+\infty )$ and

$$\begin{aligned} \frac{\partial }{\partial r} W_\Lambda (u_{x_0,r}) \geqq \frac{2}{r}\int _{\partial B_1}|x\cdot \nabla u_{x_0,r}-u_{x_0,r}|^2\, d\mathcal {H}^{d-1}, \end{aligned}$$

In particular, if $r\mapsto W_\Lambda (u_{x_0,r})$ is constant, then *u* is 1-homogeneous.

#### Proof

See for instance [38, Proposition 9.9]. $\square$

#### Lemma 4.6

Let $u_{00},v_{00}:\mathbb {R}^d\rightarrow \mathbb {R}$ be non-negative Lipschitz functions on $\mathbb {R}^d$ with the same positivity set and let $\Omega _{00}:=\{u_{00}>0\}=\{v_{00}>0\}$. Suppose that $u_{00}$ and $v_{00}$ satisfy the conditions (1)-(6) from Lemma 4.4 and let $u_{000},v_{000}:\mathbb {R}^d\rightarrow \mathbb {R}$ be blow-ups of $u_{00},v_{00}$ at zero. Then:

1. $$u_{000}=\lambda v_{000}$$ on $$\mathbb {R}^d$$;
2. for every compactly supported smooth vector field $\xi \in C^\infty _c(\mathbb {R}^d;\mathbb {R}^d)$, we have
  4.8 $$\begin{aligned} \int _{\mathbb {R}^d} \bigg (- \nabla u_{000} \cdot \big ((\nabla \xi ) + (D\xi )\big )\nabla u_{000}+\Big (|\nabla u_{000}|^2+ {\lambda }Q(x_0)\mathbbm {1}_{\Omega _{000}} \Big ){{\,\textrm{div}\,}}\xi \bigg )\, dx=0;\nonumber \\ \end{aligned}$$
3. $u_{000}$ is 1-homogeneous in $\mathbb {R}^d$.

#### Proof

Set $\Lambda :={\lambda }Q(x_0)$ and for simplicity, let $u:=u_{00}$. By Lemma 4.5, the function

$$\begin{aligned} r\mapsto W_\Lambda \big (u_r\big ), \end{aligned}$$

is non-decreasing in *r* and so, it admits a limit $\Theta$ as $r\rightarrow 0$; moreover, by the Lipschitz continuity of *u*, $\Theta$ is finite. Let $r_k\rightarrow 0$ be such that $u_{r_k}\rightarrow u_{000}$. Then, by Lemma 4.1, $u_{r_k}$ converges to $u_{000}$ strongly in $H^1_{loc}$ and the level sets $\{u_{r_k}>0\}$ converge in $L^1_{loc}$ to $\Omega _{000}$. Thus, for any $s>0$

$$\begin{aligned} \Theta :=\lim _{k\rightarrow +\infty }W_\Lambda \big (u_{sr_k}\big )=W_\Lambda \big ((u_{000})_s\big ). \end{aligned}$$

Moreover, using again the strong convergence of $u_{r_k}$ and their level sets, we get that $u_{000}$ satisfies (4.8). Thus, using again Lemma 4.5 and the fact that $s\mapsto W_\Lambda \big ((u_{000})_s\big )$ is constantly equal to $\Theta$, we get that $u_{000}$ is homogeneous. $\square$

As an immediate consequence, we obtain the following proposition.

#### Proposition 4.7

(Existence of stationary 1-homogeneous blow-ups) Let $D\subset \mathbb {R}^d$ be a bounded open set and $\Omega$ be a solution to (1.3) with *f*, *g*, *Q* as in Theorem 1.2. Let $u:=u_\Omega$ and $v:=v_\Omega$ be the state functions on $\Omega$ defined in (1.1) and (1.6) and let $x_0 \in \partial \Omega \cap D$. Then, there is a sequence $r_k\rightarrow 0$ such that the corresponding blow-up limits

$$\begin{aligned} u_0:=\lim _{k\rightarrow \infty }u_{x_0,r_k}\qquad \text {and}\qquad v_0:=\lim _{k\rightarrow \infty }v_{x_0,r_k}\, \end{aligned}$$

satisfy the following conditions:

(i) $u_0$ and $v_0$ are 1-homogeneous in $\mathbb {R}^d$ and $u_{0}=\lambda v_{0}$ for some constant $\lambda \in (C_1,C_2)$;
(ii) for every compactly supported smooth vector field $\xi \in C^\infty _c(\mathbb {R}^d;\mathbb {R}^d)$, we have
  $$\begin{aligned} \int _{\mathbb {R}^d}\bigg (-\nabla u_0\cdot \big ((\nabla \xi )+(D\xi )\big )\nabla u_0+\Big (|\nabla u_0|^2+\lambda Q(x_0)\mathbbm {1}_{\Omega _0}\Big ){{\,\textrm{div}\,}}\xi \bigg )\,dx=0. \end{aligned}$$

#### Remark 4.8

The blow-up sequence arising from Proposition 4.7 is obtained by a diagonal argument between the three different blow-ups defined respectively in Sects. 4.2, 4.3 and 4.4. Therefore, by combining all the previous result, we can see that $u_0$ and $v_0$ fulfill the conditions (a), (b), (c), d and (e) in Definition 7.3.

## Regular and Singular Parts of the Free Boundary

Let $D\subset \mathbb {R}^d$ be a bounded open set and let $\Omega$ be a solution to the problem (1.3). As in Sect. 4, we denote by *u*, *v* the associated state variables.

### Definition 5.1

(*Regular and singular points*) We will say that a boundary point $x_0\in \partial \Omega \cap D$ is *regular* if there is a blow-up limit $(u_{0},v_0)$ of (*u*, *v*) at $x_0$ such that

$$\begin{aligned} u_{0}(x) = \alpha (x \cdot \nu )_+\qquad \text{ and }\qquad v_{0}(x)=\beta (x \cdot \nu )_+\, \end{aligned}$$

for some unit vector $\nu \in \mathbb {R}^d$ and some $\alpha >0$ and $\beta >0$ such that $\alpha \beta = Q(x_0)$. If such a blow-up limit does not exist, then we will say that $x_0$ is *singular*.

We will denote by $\text { Reg}(\partial \Omega )$ the set of all regular points on $\partial \Omega \cap D$ and by $\text { Sing}(\partial \Omega )$ the set of all singular points on $\partial \Omega \cap D$. Clearly, we have that

$$\begin{aligned} \text { Reg}(\partial \Omega )\cap \text { Sing}(\partial \Omega )=\emptyset \qquad \text {and}\qquad \text { Reg}(\partial \Omega )\cup \text { Sing}(\partial \Omega )=\partial \Omega \cup D. \end{aligned}$$

In Sect. 6 we will show that the regular part $\text { Reg}(\partial \Omega )$ is in fact, locally, a smooth manifold (and in particular, a relatively open subset of $\partial \Omega \cap D$), while in Sect. 8 we will give an estimate on the dimension of the singular set $\text { Sing}(\partial \Omega )$.

### Regular and Singular Parts in Dimension Two

One can easily show that in dimension $d=2$ the free boundary $\partial \Omega \cap D$ is composed only of regular points; in particular, in dimension two the proof of Theorem 1.2 is concluded already in Sect. 6, while the results from Sect. 8 are needed only when $d\ge 3$.

#### Lemma 5.2

Let *D* be a bounded open set in $\mathbb {R}^2$ and let $\Omega$ be a solution to (1.3). Then, every point $x_0 \in \partial \Omega \cap D$ is a regular point in the sense of Definition 5.1.

#### Proof

Let $r_k\rightarrow 0$ be a sequence such that the blow-up limits

$$\begin{aligned} u_0:=\lim _{k\rightarrow \infty }u_{r_k,x_0}\qquad \text {and}\qquad v_0:=\lim _{k\rightarrow \infty }v_{r_k,x_0}, \end{aligned}$$

are, as in Proposition 4.7, proportional ($u_0=\lambda v_0$), non-negative and 1-homogeneous functions on $\mathbb {R}^d$, which are harmonic on the positivity set $\Omega _0:=\{u_0>0\}=\{v_0>0\}$. Now, reasoning as in [38, Proposition 9.13], we write $u_0$ and $v_0$ in polar coordinates as

$$\begin{aligned} u_0(r,\theta )=r\phi (\theta )\qquad \text {and}\qquad v_0(r,\theta )=r\psi (\theta ), \end{aligned}$$

where (since $u_0$ and $v_0$ are harmonic) $\phi$ and $\psi$ are solutions to

$$\begin{aligned} -\phi ''(\theta )=\phi (\theta )\quad \text {and}\quad -\psi ''(\theta )=\psi (\theta )\quad \text {in}\quad \partial \Omega _0\cap \partial B_1. \end{aligned}$$

Since the only solutions of this equations (up to a rotation) are multiples of $\sin \theta$, and since $\mathcal {H}^{1}(\Omega _0\cap \partial B_1)<2\pi$ (this follows from the density estimate in Proposition 4.3), we get that

$$\begin{aligned} u_{0}(x)=\alpha (x\cdot \nu )_+\qquad \text{ and }\qquad v_{0}(x)=\beta (x\cdot \nu )_+\, \end{aligned}$$

for some unit vector $\nu \in \mathbb {R}^d$ and some $\alpha >0$ and $\beta >0$. Moreover, by Proposition 4.7, the function $u_0$ is critical point for the one-phase problem (that is, (4.8) holds). Thus,

$$\begin{aligned} |\nabla u_0|^2={\lambda }Q(x_0)\qquad \text {on }\partial \Omega _0=\{x\in \mathbb {R}^2:\,x\cdot \nu =0\}, \end{aligned}$$

where $\lambda =\alpha /\beta$. Since $|\nabla u_0|^2=\alpha ^2$, we get that $\alpha \beta =Q(x_0)$, which concludes the proof. $\square$

### A Geometric Condition for the Regularity in Every Dimension

By an argument similar to the one in Lemma 5.2, we have that the free boundary points admitting one-sided tangent ball are regular points. This result holds in every dimension and will be useful in Sect. 6.

#### Lemma 5.3

Let *D* be a bounded open set in $\mathbb {R}^d$, $d\ge 2$, and let $\Omega$ be a solution to (1.3). Suppose that there is a one-sided tangent ball at the boundary point $x_0 \in \partial \Omega \cap D$ in the sense that:

5.1 $$\begin{aligned} \text {there is} B_r(y_0)\subset \Omega \text {with} x_0\in \partial B_r(y_0) \text {or there is} B_r(z_0)\subset \mathbb {R}^d\setminus \overline{\Omega } \text {with} x_0\in \partial B_r(z_0). \end{aligned}$$

Then, $x_0$ is a regular point in the sense of Definition 5.1.

#### Proof

As in Lemma 5.2, we consider a sequence $r_k\rightarrow 0$ for which the blow-up limits

$$\begin{aligned} u_0:=\lim _{k\rightarrow \infty }u_{r_k,x_0}\qquad \text {and}\qquad v_0:=\lim _{k\rightarrow \infty }v_{r_k,x_0}, \end{aligned}$$

are 1-homogeneous, non-negative, harmonic on their positivity set $\Omega _0:=\{u_0>0\}$ and are such that $u_0=\lambda v_0$. The one-sided ball condition implies that there is a unit vector $\nu \in \mathbb {R}^d$ such that

$$\begin{aligned} \Omega _0\subset \{x\in \mathbb {R}^d:\,x\cdot \nu>0\}\qquad \text {or}\qquad \Omega _0\supseteq \{x\in \mathbb {R}^d:\,x\cdot \nu >0\}. \end{aligned}$$

Since $\Omega _0$ satisfies an exterior density estimate (by Proposition 4.3, claim Item (3)), the only possibility is that $\Omega _0=\{x\in \mathbb {R}^d:\,x\cdot \nu >0\}$ and

$$\begin{aligned} u_{0}(x) = \alpha (x \cdot \nu )_+\qquad \text{ and }\qquad v_{0}(x)=\beta (x \cdot \nu )_+\, \end{aligned}$$

for some $\alpha >0$ and $\beta >0$ with $\alpha /\beta =\lambda$. As in Lemma 5.2, since $u_0$ satisfies (4.8), we get that

$$\begin{aligned} |\nabla u_0|^2=\frac{\alpha }{\beta }Q(x_0)\qquad \text {on }\partial \Omega _0=\{x\in \mathbb {R}^2:\,x\cdot \nu =0\}, \end{aligned}$$

which gives that $\alpha \beta =Q(x_0)$. $\square$

## Regularity of $\text { Reg}(\partial \Omega )$

In this section we prove that the regular part $\text { Reg}(\partial \Omega )$ of the boundary of an optimal set $\Omega$ is locally the graph of a smooth function.

### Viscosity Formulation

In this subsection we prove that on the free boundary $\partial \Omega \cap D$ of an optimal set $\Omega$, solution to (1.3), we have the following optimality condition

$$\begin{aligned} |\nabla u_\Omega ||\nabla v_\Omega | = Q\qquad \text{ on } \partial \Omega , \end{aligned}$$

in viscosity sense, as in [28, Section 2], in terms of the blow-up limit of the state functions $u_\Omega$ and $v_\Omega$ at free boundary points (see Remark 6.2).

#### Definition 6.1

(*Viscosity solutions*) Let *D* be a bounded open set in $\mathbb {R}^d$ and let $f,g\in L^\infty (\mathbb {R}^d)$. Let $u,v :D\rightarrow \mathbb {R}$ be two non-negative continuous functions with the same support

$$\begin{aligned} \Omega :=\{u>0\}=\{v>0\}\,, \end{aligned}$$

on which they satisfy the PDE

6.1 $$\begin{aligned} -\Delta u = f\quad \text {and}\quad -\Delta v = g \quad \text{ in } \Omega \cap D\,. \end{aligned}$$

We say that the boundary condition

$$\begin{aligned} |\nabla u||\nabla v|=Q \qquad \text{ on } \partial \Omega \cap D\,, \end{aligned}$$

holds in viscosity sense if at any point $x_0\in \partial \Omega \cap D$, at which $\Omega$ admits a one-sided tangent ball in the sense of (5.1), there exist:

- a decreasing sequence $$r_k\rightarrow 0$$;
- two positive constants $\alpha ,\beta >0$ such that $\alpha \beta =Q(x_0)$;
- a unit vector $$\nu \in \mathbb {R}^d$$;

such that the rescalings

$$\begin{aligned} u_{x_0,r_k}(x):=\frac{u(x_0+r_kx)}{r_k}\qquad \text {and}\qquad v_{x_0,r_k}(x):=\frac{v(x_0+r_kx)}{r_k}\,, \end{aligned}$$

converge uniformly in every ball $B_R\subset \mathbb {R}^d$ respectively to the blow-up limits

6.2 $$\begin{aligned} u_0(x):=\alpha \left( x\cdot \nu \right) _+\qquad \text {and}\qquad v_0(x):=\beta \left( x\cdot \nu \right) _+\ . \end{aligned}$$

#### Remark 6.2

In [28] the authors addressed the ${\varepsilon }$-regularity theory for viscosity solutions of (1.8). In particular, in [28, Lemma 2.9] they proved that if the free boundary condition is satisfied in the sense of Definition 6.1, then for any smooth function $\varphi \in C^\infty (D)$ the following holds:

(i) If $\varphi _+$ touches $\sqrt{uv}$ from below at a point $x_0\in D\cap \partial \Omega$, then $|\nabla \varphi (x_0)|\le \sqrt{Q(x_0)}$.
(ii) If $\varphi _+$ touches $\sqrt{uv}$ from above at a point $x_0\in D\cap \partial \Omega$, then $|\nabla \varphi (x_0)|\ge \sqrt{Q(x_0)}$.
(iii) If *a* and *b* are constants such that
  $$\begin{aligned} a>0,\quad b>0\qquad \text {and}\qquad a b=Q(x_0), \end{aligned}$$
  and if $\varphi _+$ touches $w_{ab}:=\frac{1}{2}(au+bv)$ from above at $x_0\in D\cap \partial \Omega$, then $|\nabla \varphi (x_0)|\ge \sqrt{Q(x_0)}$.

#### Proposition 6.3

Let $D\subset \mathbb {R}^d$ be a bounded open set and let $f,g,Q:D\rightarrow \mathbb {R}$ be as in Theorem 1.2. Let $\Omega$ be a solution to (1.3). Then the state variables $u:=u_\Omega$ and $v:=v_\Omega$ satisfy

6.3 $$\begin{aligned} |\nabla u||\nabla v|=Q\qquad \text{ on } \partial \Omega \cap D, \end{aligned}$$

in the sense of Definition 6.1.

#### Proof

It follows as in the proof of Lemma 5.3. $\square$

We can now prove that $\text { Reg}(\partial \Omega )$ is $C^{1,\alpha }$-regular for some $\alpha \in (0,1)$ by exploiting the ${\varepsilon }$-regularity theory developed in [28].

#### Theorem 6.4

(Regularity of $\text { Reg}(\partial \Omega )$) Let *D* be a bounded open set in $\mathbb {R}^d$, where $d\ge 2$. Let

$$\begin{aligned} f:D\rightarrow \mathbb {R},\quad g:D\rightarrow \mathbb {R},\quad Q:D\rightarrow \mathbb {R}, \end{aligned}$$

be given non-negative functions. Suppose that the following conditions hold:

- $$f,g\in L^\infty (D)$$;
- there are constants $C_1,C_2 > 0$ such that
  $$\begin{aligned} 0\le C_1 g\le f\le C_2 g\qquad \text{ in } D. \end{aligned}$$
- $Q\in C^{0,\alpha _Q}(D)$, for some $\alpha _Q>0$, and there are a positive constants $c_Q,C_Q$ such that
  $$\begin{aligned} 0<c_Q\le Q(x)\leqq C_Q\qquad \text{ for } \text{ every } x\in D. \end{aligned}$$

Let $\Omega$ be a solution to (1.3). Then, the regular part $\text { Reg}(\partial \Omega )$, defined in Sect. 5, is locally the graph of a $C^{1,\alpha }$ function, for some $\alpha >0$.

#### Proof

The proof follows by the epsilon-regularity theory developed in [28]. Indeed, if $x_0 \in \text { Reg}(\partial \Omega )$ and $u_{x_0,\rho _k}, v_{x_0,\rho _k}$ are the blow-up sequences from the definition of $\text { Reg}(\partial \Omega )$. Then, there are $\alpha ,\beta >0, \nu \in \mathbb {R}^d$ such that $\alpha \beta =Q(x_0), |\nu |=1$ and

$$\begin{aligned} \lim _{k\rightarrow \infty }\left\| {u_{x_0,\rho _k}-\alpha (x\cdot \nu )_+} \right\| _{L^\infty (B_1)}=0\quad \text {and}\quad \lim _{k\rightarrow \infty }\left\| {v_{x_0,\rho _k}-\beta (x\cdot \nu )_+} \right\| _{L^\infty (B_1)}=0. \end{aligned}$$

Moreover, the Lipschitz continuity and the non-degeneracy imply that for every ${\varepsilon }>0$ there exists $k_0>0$ such that for every $k\geqq k_0$ we have

$$\begin{aligned} \alpha (x\cdot \nu -{\varepsilon })_+\le&u_{x_0,\rho _k}\le \alpha (x\cdot \nu + {\varepsilon })_+\qquad \text {for every }x\in B_1,\\ \beta (x\cdot \nu -{\varepsilon })_+\le&v_{x_0,\rho _k}\le \beta (x\cdot \nu + {\varepsilon })_+\qquad \text {for every }x\in B_1, \end{aligned}$$

that is, $u_{x_0,\rho _k}, v_{x_0,\rho _k}$ are ${\varepsilon }$-flat in the direction $\nu$ (see [28, Definition 1.2]). By rescaling the associated state equations, we have

$$\begin{aligned} -\Delta u_{x_0,\rho _k} = \rho _k^2 f_{x_0,\rho _k} \quad \text {and}\quad -\Delta v_{x_0,\rho _k} = \rho _k^2 g_{x_0,\rho _k}\quad \text{ in }\quad B_1\cap \{u_{x_0,\rho _k}>0\}, \end{aligned}$$

where

$$\begin{aligned} \left\| {\Delta u_{x_0,\rho _k}} \right\| _{L^\infty (B_1)}+ \left\| {\Delta v_{x_0,\rho _k}} \right\| _{L^\infty (B_1)} \leqq \rho _k\left( \left\| {f} \right\| _{L^\infty (B_1)}+\left\| {g} \right\| _{L^\infty (B_1)}\right) . \end{aligned}$$

On the other hand, since both $u_{x_0,\rho _k}$ and $v_{x_0,\rho _k}$ still satisfy (6.3) in viscosity sense, by applying [28, Theorem 3.1], $\partial \{u_{x_0,\rho _k}>0\}$ is $C^{1,\alpha }$ in $B_{1/2}$. Finally, the result follows by rescaling back to the original problem. $\square$

### Higher Regularity

We can pass from $C^{1,\alpha }$ to $C^\infty$-regularity of $\text { Reg}(\partial \Omega )$ by exploiting the higher order Boundary Harnack Principle for solutions to (1.8).

#### Proposition 6.5

(Higher regularity of $\text { Reg}(\partial \Omega )$) Let *D* be a bounded open set in $\mathbb {R}^d$ and let *f*, *g*, *Q* be as in Theorem 6.4. If $f,g,Q\in C^{k,\alpha }(D)$ for some $k\ge 1$ and $\alpha >0$, then the regular part $\text { Reg}(\partial \Omega )$ of the free boundary $\partial \Omega \cap D$ of any solution $\Omega$ to (1.3) is locally the graph of a $C^{1+k,\alpha }$ function for some $\alpha >0$. In particular, if *f*, *g*, *Q* are $C^\infty$, then $\text { Reg}(\partial \Omega )$ is locally the graph of a $C^\infty$ function.

#### Proof

We use a bootstrap argument as in [31, Section 5.4]. Suppose that $\text { Reg}(\partial \Omega )$ is locally the graph of a $C^{k,\alpha }$-regular function, for some $k\geqq 1$ (when $k=1$ the claim follows from Theorem 6.4). Let $x_0 \in \partial \Omega$ and $r>0$ be such that $\partial \Omega \cap B_r(x_0)= \text { Reg}(\partial \Omega )$. Since *u* and *v* satisfy (6.1) in $\Omega$ and since *v* is non-degenerate, we can apply the higher order Boundary Harnack Principle (for PDEs with right-hand side) from [27, Theorem 1.3] and [36, Theorem 1.3], obtaining a non-negative $C^{k,\alpha }$-regular function $w:B_r(x_0)\cap \overline{\Omega }\rightarrow \mathbb {R}$ satisfying $u =w v$ in $B_r(x_0)\cap \overline{\Omega }$. Then, we have:

$$\begin{aligned} |\nabla u||\nabla v|=Q\quad \text {and}\quad |\nabla u|=w|\nabla v|\quad \text {on}\quad \partial \Omega \cap B_r(x_0). \end{aligned}$$

Thus, *u* is a solution to the problem

$$\begin{aligned} -\Delta u=f\quad \text {in }\Omega \cap B_r(x_0)\,\qquad |\nabla u||\nabla u|=\sqrt{wQ}\quad \text {on }\partial \Omega \cap B_r(x_0), \end{aligned}$$

which by [24, Theorem 2] implies that $\text { Reg}(\partial \Omega )$ is locally the graph of a $C^{k+1,\alpha }$ function. $\square$

## Stable Homogeneous Solutions of the One-Phase Bernoulli Problem

In this section we study the singular set of the stable global solutions of the one-phase Bernoulli problem (see Definition 7.3 for the definition of global stable solution). The main results are Theorems 7.8 and 7.9, which we will use in Theorem 8.1 in order to estimate the dimension of the singular set of the free boundary of the optimal sets for (1.3). The present section can be read separately from the rest of the paper; we will use only the Taylor expansions from Sect. 2 and the general results from Sect. 4.

### Solutions of PDEs in Unbounded Domains

In $\mathbb {R}^d$, $d\ge 3$, we define

$$\begin{aligned} \dot{H}^1(\mathbb {R}^d):=\Big \{u\in L^{2^*}(\mathbb {R}^d)\ :\ \nabla u\in L^2(\mathbb {R}^d;\mathbb {R}^d)\Big \}\quad \text{ where }\quad 2^*:=\frac{2d}{d-2}. \end{aligned}$$

It is well known that $\dot{H}^1(\mathbb {R}^d)$ is a Hilbert space equipped with the norm $\Vert u\Vert _{\dot{H}^1(\mathbb {R}^d)}:=\Vert \nabla u\Vert _{L^2(\mathbb {R}^d;\mathbb {R}^d)}$ and that $C^\infty _c(\mathbb {R}^d)$ is dense in $\dot{H}^1(\mathbb {R}^d)$. Moreover, given an open (bounded or unbounded) set $\Omega \subset \mathbb {R}^d$, we define the space $\dot{H}^1_0(\Omega )$ as the closure of $C^\infty _c(\Omega )$ with respect to $\Vert \cdot \Vert _{\dot{H}^1(\mathbb {R}^d)}$.

Let $\Omega$ be an open (bounded or unbounded) subset of $\mathbb {R}^d$ and let $F\in L^2(\mathbb {R}^d;\mathbb {R}^d)$ be a given vector field. We say that *w* is a weak solution of the PDE

7.1 $$\begin{aligned} -\Delta w={{\,\textrm{div}\,}}\,F\quad \text {in }\Omega ,\qquad w\in \dot{H}^1_0(\Omega ), \end{aligned}$$

if $$w\in \dot{H}^1_0(\Omega )$$ and

$$\begin{aligned} \int _{\mathbb {R}^d}\nabla w\cdot \nabla \varphi \,dx=-\int _{\mathbb {R}^d}\nabla \varphi \cdot F\,dx\qquad \text {for every }\varphi \in \dot{H}^1_0(\Omega ). \end{aligned}$$

It is standard to check that *w* is a solution to (7.1) if and only if *w* minimizes the functional

7.2 $$\begin{aligned} J(\varphi ):=\frac{1}{2}\int _{\mathbb {R}^d}|\nabla \varphi |^2\,dx+\int _{\mathbb {R}^d}\nabla \varphi \cdot F\,dx, \end{aligned}$$

among all functions $\varphi \in \dot{H}^1_0(\Omega )$. Since it is immediate to check that a minimizer of (7.2) in $\dot{H}^1_0(\Omega )$ exists and is unique, we get that also the solution to (7.1) exists and is unique. Finally, we notice that if $w\in \dot{H}^1_0(\Omega )$ is the solution to (7.1), then

$$\begin{aligned} \int _{\mathbb {R}^d}|\nabla w|^2\,dx=-\int _{\mathbb {R}^d}\nabla w\cdot F\le \Vert F\Vert _{L^2}\Vert \nabla w\Vert _{L^2}, \end{aligned}$$

which gives

7.3 $$\begin{aligned} \int _{\mathbb {R}^d}|\nabla w|^2\,dx\le \int _{\mathbb {R}^d}|F|^2\,dx. \end{aligned}$$

#### Remark 7.1

(Exterior density estimate and the space $\dot{H}^1_0$) We say that an open set $\Omega \subset \mathbb {R}^d$ satisfies a uniform exterior density estimate with a constant $c>0$ if

7.4 $$\begin{aligned} |B_r(x)\setminus \Omega |\ge c|B_r|\quad \text {for every}\quad r\in (0,1)\quad \text {and every}\quad x\in \mathbb {R}^d\setminus \Omega . \end{aligned}$$

It is known (see for example [17]) that if an open set $\Omega \subset \mathbb {R}^d$ satisfies (7.4) then the space $\dot{H}^1_0(\Omega )$ can be characterized as:

7.5 $$\begin{aligned} \dot{H}^1_0(\Omega )=\Big \{u\in \dot{H}^1(\mathbb {R}^d)\ :\ u=0\ \text{ a.e. } \text{ on } \ \mathbb {R}^d\setminus \Omega \Big \}. \end{aligned}$$

#### Lemma 7.2

(Convergence of solutions) Let $\Omega _n$ be a sequence of open sets in $\mathbb {R}^d$, $d\ge 3$ such that:

- there is a constant $c>0$ such that, for every $n\ge 1$, $\Omega _n$ satisfies the exterior density estimate (7.4);
- there is an open set $\Omega _\infty \subset \mathbb {R}^d$ satisfying the exterior density estimate (7.4) with the constant $c>0$ and such that the sequence of characteristic functions $\mathbbm {1}_{\Omega _n}$ converges pointwise almost-everywhere in $\mathbb {R}^d$ to $\mathbbm {1}_{\Omega _\infty }$.

Let $F_n\in L^2(\mathbb {R}^d;\mathbb {R}^d)$ be a sequence of vector fields converging strongly in $L^2(\mathbb {R}^d;\mathbb {R}^d)$ to the vector field $F_\infty \in L^2(\mathbb {R}^d;\mathbb {R}^d)$. For every $n\ge 1$, let $w_n$ be the solution to the PDE

$$\begin{aligned} -\Delta w_n={{\,\textrm{div}\,}}\,F_n\quad \text {in}\quad \Omega _n\,\qquad w_n\in \dot{H}^1_0(\Omega _n). \end{aligned}$$

Then, $w_n$ converges strongly in $\dot{H}^1(\mathbb {R}^d)$ to the solution $w_\infty$ of

$$\begin{aligned} -\Delta w_\infty ={{\,\textrm{div}\,}}\,F_\infty \quad \text {in}\quad \Omega _\infty \,\qquad w_\infty \in \dot{H}^1_0(\Omega _\infty ). \end{aligned}$$

#### Proof

First of all, we notice that the sequence $w_n$ is bounded in $\dot{H}^1(\mathbb {R}^d)$ (by (7.3)). Thus, we can extract a subsequence, that we still denote by $w_{n}$, which converges weakly in $\dot{H}^1(\mathbb {R}^d)$ and pointwise almost-everywhere on $\mathbb {R}^d$ to a function $w\in \dot{H}^1(\mathbb {R}^d)$. We will show that $w=w_\infty$.

First, notice that for almost every $x\in \mathbb {R}^d\setminus \Omega _\infty$ we have that

$$\begin{aligned} w_n(x)\rightarrow w(x)\qquad \text {and}\qquad \mathbbm {1}_{\Omega _n}(x)\rightarrow \mathbbm {1}_{\Omega _\infty }(x)=0. \end{aligned}$$

But then $w_n(x)=0$ (by (7.5)) and so $w(x)=0$. Thus, using again (7.5), we get $w\in \dot{H}^1_0(\Omega _\infty )$.

Now, let $\varphi \in C^\infty _c(\Omega _\infty )$. We will show that for large enough *n*, $\varphi \in C^\infty _c(\Omega _n)$. Indeed, let $\delta >0$ be a constant such that $B_\delta (x)\subset \Omega _\infty$ for every *x* in the support of $\varphi$. Suppose by contradiction that there is a sequence $x_n\in \overline{\{\varphi \ne 0\}}$ such that $x_n\notin \Omega _n$. Then, by the density estimate for $\Omega _n$, $|B_\delta (x_n)\cap \Omega _n|\le (1-c)|B_{\delta }|$. Now, up to a subsequence, $x_n\rightarrow x_\infty \in \overline{\{\varphi \ne 0\}}$. But then,

$$\begin{aligned} (1-c)|B_{\delta }|\ge \lim _{n\rightarrow \infty }|B_\delta (x_n)\cap \Omega _n|=|B_\delta (x_\infty )\cap \Omega _\infty |=|B_\delta |, \end{aligned}$$

which is a contradiction. Thus, for large *n*, $\overline{\{\varphi \ne 0\}}\subset \Omega _n$. Now, using the equation for $w_n$,

$$\begin{aligned} \int _{\mathbb {R}^d}\nabla w_n\cdot \nabla \varphi \,dx=-\int _{\mathbb {R}^d}\nabla \varphi \cdot F_n\,dx\, \end{aligned}$$

and passing to the limit, we get that

$$\begin{aligned} \int _{\mathbb {R}^d}\nabla w_\infty \cdot \nabla \varphi \,dx=-\int _{\mathbb {R}^d}\nabla \varphi \cdot F_\infty \,dx, \end{aligned}$$

that is $$w=w_\infty $$.

Finally, in order to prove that the convergence is strong, we use the equations for $w_n$ and $w_\infty$ and the strong convergence of $F_n$ to $F_\infty$:

$$\begin{aligned} \int _{\mathbb {R}^d}|\nabla w_n|^2\,dx=-\int _{\mathbb {R}^d}\nabla w_n\cdot F_n\,dx\rightarrow -\int _{\mathbb {R}^d}\nabla w_\infty \cdot F_\infty \,dx=\int _{\mathbb {R}^d}|\nabla w_\infty |^2\,dx, \end{aligned}$$

which concludes the proof. $\square$

### Global Stable Solutions of the One-Phase Problem

We define the functionals

$$\begin{aligned} \delta \mathcal G: C^{0,1}(\mathbb {R})\times C^\infty _c(\mathbb {R}^d;\mathbb {R}^d)\rightarrow \mathbb {R}\qquad \text {and}\qquad \delta ^2\mathcal G: C^{0,1}(\mathbb {R})\times C^\infty _c(\mathbb {R}^d;\mathbb {R}^d)\rightarrow \mathbb {R}, \end{aligned}$$

as follows. Given:

- a Lipschitz function $$u:\mathbb {R}^d\rightarrow \mathbb {R}$$,
- a smooth compactly supported vector field $\xi \in C^\infty _c(\mathbb {R}^d;\mathbb {R}^d)$,

we set:

$$\begin{aligned} \delta \mathcal G(u)[\xi ]:=\int _{\mathbb {R}^d} \Big (\nabla u \cdot \delta A\nabla u+ \mathbbm {1}_{\Omega _u} {{\,\textrm{div}\,}}\xi \Big )\, dx, \end{aligned}$$

7.6 $$\begin{aligned} \begin{aligned} \delta ^2 \mathcal G(u)[\xi ]&:=\, \int _{\mathbb {R}^d}2\nabla u\cdot (\delta ^2 A)\nabla u-2|\nabla (\delta u)|^2\,dx\\  &\qquad +\int _{\mathbb {R}^d}\mathbbm {1}_{\Omega _u}\Big (({{\,\text {div}\,}}\xi )^2+\xi \cdot \nabla ({{\,\text {div}\,}}\,\xi )\Big )\,dx, \end{aligned} \end{aligned}$$

where $\Omega _u:=\{u>0\}$, and where $\delta A,\,\delta ^2A$ are defined as in (2.6) and where $\delta u\in \dot{H}^1_0(\Omega _u)$ is the weak solution to the PDE

7.7 $$\begin{aligned} -\Delta (\delta u)={{\,\text {div}\,}}\big ((\delta A)\nabla u\big )\quad \text{ in } \Omega _u\ ,\qquad \delta u\in \dot{H}^1_0(\Omega _u). \end{aligned}$$

#### Definition 7.3

(*Global stable solutions of the one-phase problem*) We say that a function $u:\mathbb {R}^d\rightarrow \mathbb {R}$ is a global stable solution of the one-phase problem if, for every compactly supported smooth vector field $\xi \in C^\infty _c(\mathbb {R}^d;\mathbb {R}^d)$, we have:

7.8 $$\begin{aligned} \delta \mathcal G(u)[\xi ]=0\qquad \text {and}\qquad \delta ^2 \mathcal G(u)[\xi ]\ge 0, \end{aligned}$$

and if the following conditions hold:

- *u* is globally Lipschitz continuous and non-negative on $\mathbb {R}^d$;
- *u* is harmonic in the open set $\Omega _u:=\{u>0\}$;
- there is a constant $c>0$ such that
  $$\begin{aligned} |B_r(x_0)\cap \Omega _u| \leqq (1-c)|B_r|, \end{aligned}$$
  for every $x_0\in \mathbb {R}^d\setminus \Omega _u$ and every $r>0$;
- $0\in \partial \Omega _u$ and there is a constant $\eta > 0$ such that
  $$\begin{aligned} \sup _{B_r(x_0)} u \ge \eta r\quad \text {for every}\quad x_0\in {\overline{\Omega }}_{u}\quad \text {and every}\quad r>0; \end{aligned}$$
- there is a constant $C>0$ such that, for every $R>0$,
  $$\begin{aligned} \left| \int _{B_{R}}\nabla u\cdot \nabla \varphi \,dx\right| \le C R^{d-1}\Vert \varphi \Vert _{L^\infty (B_{R})}\quad \text {for every}\quad \varphi \in C^\infty _c(B_R). \end{aligned}$$

#### Remark 7.4

The functionals $\delta \mathcal {G}$ and $\delta ^2\mathcal {G}$ correspond to the first and the second variation along vector fields of the one-phase Alt–Caffarelli functional^1^

$$\begin{aligned} \mathcal {G}(u)=\int _{\mathbb {R}^d}\Big (|\nabla u|^2+\mathbbm {1}_{\{u>0\}}\Big )\,dx, \end{aligned}$$

so one may expect that the natural definition of a global stable solution is a function $u:\mathbb {R}^d\rightarrow \mathbb {R}$ that satisfies (7.8). Unfortunately, the condition (7.8) alone seems to be quite weak. For instance,

$$\begin{aligned} u(x,y)=1+|x|\quad \text {e}\quad u(x,y)=1+|xy|\, \end{aligned}$$

satisfy (7.8), but they are not even harmonic in $\{u>0\}$, while the function

$$\begin{aligned} u(x,y)=|xy|, \end{aligned}$$

is harmonic in $\{u>0\}$, but the free boundary $\partial \{u>0\}$ has a cross-like singularity in zero; more generally, the solutions of optimal partition problems satisfy (7.8) and the structure of their nodal sets can be very different from the one of the one-phase free boundaries.

We add the conditions (a), (b), (c), d, (e) in order to have a regularity theory for one-phase stable solutions, which is similar to the one available for minimizers of the Alt–Caffarelli functional. The Lipschitz continuity (a) and the non-degeneracy d guarantee the existence of non-trivial blow-up limits obtained by 1-homogeneous rescalings of *u*. The condition (e) is needed for the strong convergence of the blow-up sequences (see Lemma 4.1), which together with the exterior density estimate (c) allows to transfer the stability condition (7.8) to the blow-up limits of *u* (thanks to Lemma 7.2). We also notice that the conditions (e) and (b) imply the Lipschitz continuity of *u*, so we could actually avoid adding (a) to the list.

Finally, we highlight that the conditions (a)–(b)–(c)-d–(e) are satisfied by the blow-ups of numerous one-phase problems, for instance by the state functions on the domains minimizing (1.3) (see Sect. 4), and of course, by the global minimizers of the classical one-phase Bernoulli problem.

#### Remark 7.5

(Blow-ups of global stable solutions) We notice that if $u:\mathbb {R}^d\rightarrow \mathbb {R}$ satisfies (a), (b), (c), d and (e), then any blow-up $u_0:\mathbb {R}^d\rightarrow \mathbb {R}$ of *u* at $x_0\in \partial \Omega _u$,

$$\begin{aligned} u_0=\lim _{n\rightarrow \infty }u_{x_0,r_n}\,\quad \text {with}\quad u_{x_0,r_n}(x):=\frac{u(x_0+r_nx)}{r_n}\quad \text {and}\quad \lim _{n\rightarrow \infty }r_n=0, \end{aligned}$$

still satisfies (a), (b), (c), d and (e). In particular, by Lemma 4.1, this means that the convergence $u_{x_0,r_n}\rightarrow u_0$ is strong in $H^1_{loc}$ and thus, by Lemmas 7.2 and 4.5, if *u* is a global stable solution, then $u_0$ is a 1-homogeneous global stable solution.

#### Remark 7.6

(Decomposition of the free boundary) Let $u:\mathbb {R}^d\rightarrow \mathbb {R}$ be a global stable solution in the sense of Definition 7.3 and let $\Omega _u:=\{u>0\}$. We decompose the free boundary $\partial \Omega _u$ as

$$\begin{aligned} \partial \Omega _u=\text { Reg}(\partial \Omega _u)\cup \text { Sing}(\partial \Omega _u), \end{aligned}$$

where the regular part $\text { Reg}(\partial \Omega _u)$ consists of all points $x_0\in \partial \Omega _u$ at which there is a blow-up limit $u_0$ which is a half-space solution, that is,

$$\begin{aligned} u_0(x)=(x\cdot \nu )_+\quad \text {for some unit vector}\quad \nu \in \mathbb {R}^d, \end{aligned}$$

while the singular part is given by $\text { Sing}(\partial \Omega _u)=\partial \Omega _u\setminus \text { Reg}(\partial \Omega _u)$. As in Sect. 6, it is immediate to check that the global stable solutions satisfy the optimality condition

$$\begin{aligned} |\nabla u|=1\quad \text {on}\quad \partial \Omega _u \end{aligned}$$

in viscosity sense, so the ${\varepsilon }$-regularity theorem of [18] holds and we have that the regular part is a relatively open subset of $\partial \Omega _u$ and a $C^\infty$ manifold. We notice that this decomposition is precisely the one from Sect. 5 with $\alpha =\beta =Q=1$, $u=v$, and $f=g=0$.

#### Definition 7.7

(*Critical dimension for stable solutions*) We define $d^*$ to be the smallest dimension admitting a 1-homogeneous global stable solution (in the sense of Definition 7.3) $u:\mathbb {R}^d\rightarrow \mathbb {R}$ with $0\in \text { Sing}(\partial \Omega _u)$.

#### Theorem 7.8

(Dimension reduction for stable solutions) Suppose that $u:\mathbb {R}^d\rightarrow \mathbb {R}$ is a 1-homogeneous global stable solution of the one-phase problem (in the sense of Definition 7.3).

(i) If $d< d^*$, then *u* is a half-plane solution.
(ii) If $d=d^*$, then $\text { Sing}(\partial \Omega _u)=\{0\}$ or *u* is a half-plane solution.
(iii) If $d>d^*$, then the Hausdorff dimension of $\text { Sing}(\partial \Omega _u)$ is at most $d-d^*$, that is,
  $$\begin{aligned} \mathcal {H}^{d-d^*+{\varepsilon }}\big (\text { Sing}(\partial \Omega _u)\big )=0\quad \text {for every}\quad {\varepsilon }>0, \end{aligned}$$

where $d^*$ is the critical dimension from Definition 7.7.

#### Proof

The proof follows from a classical argument that can be found for instance in [38] (in particular, [38, Proposition 10.13]). $\square$

#### Theorem 7.9

(Bounds on the critical dimension for stable solutions) $5\le d^*\le 7$, where $d^*$ is the critical dimension from Definition 7.7.

#### Proof

The claim follows from Propositions 7.11 and 7.12 below. $\square$

### Global Minimizers and Global Stable Solutions

Given an open set $D\subset \mathbb {R}^d$ and a function $u\in H^1(D)$, we define:

$$\begin{aligned} \mathcal G(u,D):=\int _{D}\Big (|\nabla u|^2+\mathbbm {1}_{\{u>0\}}\Big )\,dx. \end{aligned}$$

#### Definition 7.10

(*Global minimizers*) We say that a function $u:\mathbb {R}^d\rightarrow \mathbb {R}$ is a global minimizer of the Alt–Caffarelli functional, if:

- *u* is non-negative and $u\in H^1_{loc}(\mathbb {R}^d)$;
- $\mathcal G(u,B_R)\le \mathcal G(v,B_R)$, for every $B_R\subset \mathbb {R}^d$ and every $v:\mathbb {R}^d\rightarrow \mathbb {R}$ such that $v-u\in H^1_0(B_R)$.

#### Proposition 7.11

Suppose that $u:\mathbb {R}^d\rightarrow \mathbb {R}$ is a global minimizer in the sense of Definition 7.10. Then, *u* is a global stable solution in the sense of Definition 7.3. In particular, $d^*\le 7$, where $d^*$ is the critical dimension from Definition 7.7.

#### Proof

It is well-known that the global minimizers satisfy the conditions (a)-(b)-(c)-d-(e) of Definition 7.3 (see for instance [38]). Moreover, by [38, Lemma 9.5 and Lemma 9.6], the global minimizers are critical points, that is,

$$\begin{aligned} \delta \mathcal {G}(u)[\xi ]=0\qquad \text {for every}\qquad \xi \in C^\infty _c(\mathbb {R}^d;\mathbb {R}^d). \end{aligned}$$

Thus, it only remains to prove the positivity of the second variation:

$$\begin{aligned} \delta ^2 \mathcal G(u)[\xi ]\ge 0\qquad \text {for every}\qquad \xi \in C^\infty _c(\mathbb {R}^d;\mathbb {R}^d). \end{aligned}$$

Let $\xi \in C^\infty _c(\mathbb {R}^d;\mathbb {R}^d), \Phi _t$ be the associated defined by (2.5) and let $\Omega _t:= \Phi _t(\Omega )$, for $t\in \mathbb {R}$. In $B_R$, we consider the solution $u_t$ to the problem

$$\begin{aligned} -\Delta u_t=0\quad \text {in}\quad \Omega _t\cap B_R,\qquad u_t=0\quad \text {on}\quad \partial \Omega _t\cap B_R, \qquad u_t=u\quad \text {on}\quad \partial B_R. \end{aligned}$$

By the optimality of $u_t$, we have that

$$\begin{aligned} \mathcal G(u_t,B_R)\ge \mathcal G(u,B_R)\quad \text {for every}\quad t\in \mathbb {R}, \end{aligned}$$

so,

$$\begin{aligned} \frac{d}{dt}\Big |_{t=0}\mathcal G(u_t,B_R)=0\quad \text {and}\quad \frac{d^2}{dt^2}\Big |_{t=0}\mathcal G(u_t,B_R)\ge 0. \end{aligned}$$

By Lemma 2.8, we have that

$$\begin{aligned} \begin{aligned}&\frac{d^2}{dt^2}\Big |_{t=0}\mathcal G(u_t,B_R):=\, \int _{B_R}2\nabla u\cdot (\delta ^2 A)\nabla u-2|\nabla w_R|^2\\&+\mathbbm {1}_{\Omega _u}\Big (({{\,\textrm{div}\,}}\xi )^2+\xi \cdot \nabla ({{\,\textrm{div}\,}}\,\xi )\Big )\,dx, \end{aligned} \end{aligned}$$

where $\Omega _u=\{u>0\}$ and $w_R$ is the solution to the PDE

$$\begin{aligned} -\Delta w_R={{\,\textrm{div}\,}}\big ((\delta A)\nabla u\big )\quad \text {in}\quad \Omega _u\cap B_R\,\qquad w_R\in H^1_0(\Omega _u\cap B_R). \end{aligned}$$

Thus, for every $R>0$,

$$\begin{aligned} \int _{\mathbb {R}^d}|\nabla w_R|^2\,dx\ge \, \int _{B_R}\nabla u\cdot (\delta ^2 A)\nabla u+\frac{1}{2}\mathbbm {1}_{\Omega _u}\Big (({{\,\textrm{div}\,}}\xi )^2+\xi \cdot \nabla ({{\,\textrm{div}\,}}\,\xi )\Big )\,dx\,. \end{aligned}$$

Since by Lemma 7.2 the sequence $w_R\rightarrow \delta u$ strongly in $\dot{H}^1(\mathbb {R}^d)$ as $R\rightarrow \infty$, we get that

$$\begin{aligned} \int _{\mathbb {R}^d}|\nabla (\delta u)|^2\,dx\ge \, \int _{B_R}\nabla u\cdot (\delta ^2 A)\nabla u+\frac{1}{2}\mathbbm {1}_{\Omega _u}\Big (({{\,\textrm{div}\,}}\xi )^2+\xi \cdot \nabla ({{\,\textrm{div}\,}}\,\xi )\big )\Big )\,dx\,, \end{aligned}$$

which is precisely the inequality $\delta ^2\mathcal {G}(u)[\xi ]\ge 0$. Finally, the bound $d^*\le 7$ follows by the example of a singular 1-homogeneous global minimizer constructed by De Silva and Jerison in [19]. $\square$

### Global Stable Solutions and the Stability Inequality of Caffarelli–Jerison–Kenig

Let $u:\mathbb {R}^d\rightarrow \mathbb {R}$ be a 1-homogeneous global stable solution of the one-phase problem with an isolated singularity in zero, that is,

$$\begin{aligned} \text { Sing}(\partial \Omega _u)=\{0\}. \end{aligned}$$

In particular, we have that the regular part

$$\begin{aligned} \text { Reg}(\partial \Omega _u):=\partial \Omega _u\setminus \{0\}, \end{aligned}$$

is a smooth $C^\infty$ manifold and the function *u* is $C^\infty$ in ${\overline{\Omega }}_u\setminus \{0\}$, up to the boundary $\partial \Omega _u\setminus \{0\}$. Thus, *u* is a classical solution to the PDE

7.9 $$\begin{aligned} \Delta u=0\quad \text {in }\Omega _u,\qquad |\nabla u|=1\quad \text {on }\partial \Omega _u\setminus \{0\}. \end{aligned}$$

Together with the homogeneity of *u* this implies (see for instance [22]) that

$$\begin{aligned} H>0\quad \text {on }\partial \Omega _u\setminus \{0\}, \end{aligned}$$

where *H* is the mean curvature of $\partial \Omega _u$ oriented towards the complement of $\Omega _u$.

We will say that $\Omega _u$ supports the *stability inequality of Caffarelli-Jerison-Kenig* if

7.10 $$\begin{aligned} \int _{\Omega _u}|\nabla \varphi |^2\,dx\ge \int _{\partial \Omega _u}H\varphi ^2\,d\mathcal {H}^{d-1}\qquad \text {for every }\varphi \in C^\infty _c(\mathbb {R}^d\setminus \{0\}). \end{aligned}$$

In [14] and [22] it was shown that if:

- $d=3$ (see [14]) or $d=4$ (see [22]);
- $u:\mathbb {R}^d\rightarrow \mathbb {R}$ is a 1-homogeneous non-negative Lipschitz function;
- $$\partial \Omega _u\setminus \{0\}$$ is $$C^\infty $$ smooth;
- *u* is a solution of the one-phase Bernoulli problem (7.9);
- $\Omega _u$ supports the stability inequality (7.10);

then *u* is a half space solution, that is,

$$\begin{aligned} u(x)=(x\cdot \nu )_+\quad \text {for some unit vector}\quad \nu \in \mathbb {R}^d. \end{aligned}$$

Thus, in order to show that in dimension 3 and 4 there are no global stable solutions (in the sense of Definition 7.3) with singularities, it is sufficient to prove the following proposition.

#### Proposition 7.12

(The global stable cones satisfy the stability inequality) Let $u:\mathbb {R}^d\rightarrow \mathbb {R}$ be a 1-homogeneous global stable solution (in the sense of Definition 7.3) with $\text { Sing}(\partial \Omega _u)=\{0\}$. Then, $\Omega _u$ supports the stability inequality (7.10). In particular, $d^*\ge 5$, where $d^*$ is the critical dimension from Definition 7.7.

#### Remark 7.13

Following the proof of Proposition 7.12, it is immediate to check that also the converse is true. Precisely, if $u:\mathbb {R}^d\rightarrow \mathbb {R}$ is a 1-homogeneous function, if $\partial \Omega _u\setminus \{0\}$ is smooth and if *u* is a solution to (7.9) such that $\Omega _u$ supports the stability inequality (7.10), then *u* is a global stable solution in the sense of Definition 7.3.

#### Proof

For any bounded open set $D\subset \mathbb {R}^d$ and any function $u\in H^1(D)$, we define:

$$\begin{aligned} \mathcal G(u,D):=\int _D|\nabla u|^2\,dx+|D\cap \{u>0\}|. \end{aligned}$$

Moreover, for any $R>1$, we call the annulus

$$\begin{aligned} A_R:=B_R\setminus {\overline{B}}_{{1}/{R}}. \end{aligned}$$

We fix a smooth vector field $\xi \in C^\infty _c(A_R,\mathbb {R}^d)$ and we define the open set

$$\begin{aligned} \Omega _t:=\Phi _t(\Omega _u)\quad \text {for every}\quad t\in \mathbb {R}, \end{aligned}$$

where $\Phi _t$ is the flow of $\xi$ defined by (2.5). Let $u_t:A_R\rightarrow \mathbb {R}$ be the solution of the PDE

$$\begin{aligned} \Delta u_t=0\quad \text {in}\quad \Omega _t\cap A_R,\qquad u_t=0\quad \text {on}\quad \partial \Omega _t\cap A_R,\qquad u_t=u\quad \text {in}\quad \Omega _t\cap \partial A_R. \end{aligned}$$

**Step 1.** We will show that

7.11 $$\begin{aligned} \frac{d^2}{dt^2}\Big |_{t=0}\mathcal G(u_t,A_R)\ge \delta ^2\mathcal {G}(u)[\xi ], \end{aligned}$$

where $\delta ^2\mathcal {G}$ is defined in (7.6). Following Lemma 2.8 we have that

$$\begin{aligned}&\frac{d^2}{dt^2}\Big |_{t=0}\mathcal G(u_t,A_R)\\&=\int _{A_R\cap \Omega _u}2\nabla u\cdot (\delta ^2 A)\nabla u +\Big (({{\,\textrm{div}\,}}\xi )^2+\xi \cdot \nabla ({{\,\textrm{div}\,}}\,\xi )\Big )-2|\nabla w_R|^2\,dx. \end{aligned}$$

where $\delta A$ and $\delta ^2A$ are defined in (2.6) and $w_R$ is the solution of the PDE

7.12 $$\begin{aligned} -\Delta w_R={{\,\textrm{div}\,}}\big ((\delta A)\nabla u\big )\quad \text {in }\Omega _u\cap A_R\,\qquad w_R\in H^1_0(\Omega _u\cap A_R). \end{aligned}$$

Now, let $\delta u$ be the solution to (7.7). By the variational characterization of (7.7) in $\dot{H}^1_0(\Omega _u)$, the fact that $w_R\in \dot{H}^1_0(\Omega _u)$, and an integration by parts, we have that

$$\begin{aligned} -\frac{1}{2}\int _{B_R}|\nabla (\delta u)|^2\,dx&=\frac{1}{2}\int _{B_R}|\nabla (\delta u)|^2\,dx+\int _{B_R}\nabla (\delta u)\cdot (\delta A)\nabla u\,dx\\&\le \frac{1}{2}\int _{B_R}|\nabla w_R|^2\,dx+\int _{B_R}\nabla w_R\cdot (\delta A)\nabla u\,dx\\&=-\frac{1}{2}\int _{B_R}|\nabla w_R|^2\,dx, \end{aligned}$$

which gives (7.11).

**Step 2.** We set $u'$ to be the solution of the PDE

$$\begin{aligned} {\left\{ \begin{array}{ll} \Delta u' = 0 &  \text{ in }\quad \Omega _u\cap A_{R},\\ u' = \xi \cdot \nu &  \text{ on }\quad \partial \Omega _u\cap A_{R},\\ u' = 0 &  \text{ on }\quad \Omega _u\cap \partial A_{R}, \end{array}\right. } \end{aligned}$$

where $\nu$ is the outer normal to $\partial \Omega _u$. We will first show that

$$\begin{aligned} u'=w_R-\xi \cdot \nabla u. \end{aligned}$$

Indeed, since $\xi$ is supported in $A_R$ and since $\nabla u=-\nu$ on $\partial \Omega _u\cap A_R$, we have:

$$\begin{aligned} u'=w_R-\xi \cdot \nabla u\quad \text {on}\quad \partial (\Omega _u\cap A_R). \end{aligned}$$

In order to show that $u'$ is harmonic in $\Omega _u$, we compute (using the repeated index summation convention)

$$\begin{aligned} {{\,\textrm{div}\,}}\big ((\delta A)\nabla u\big )&=\partial _j\big [-\partial _i\xi _j\partial _iu-\partial _j\xi _i\partial _iu+\partial _i\xi _i\partial _ju\big ]\\&=-\partial _{ij}\xi _j\partial _iu-\partial _i\xi _j\partial _{ij}u-\partial _{jj}\xi _i\partial _iu-\partial _j\xi _i\partial _{ij}u+\partial _{ij}\xi _i\partial _ju\\&=-2\partial _i\xi _j\partial _{ij}u-\partial _{jj}\xi _i\partial _iu=-\partial _j\big [\partial _j\xi _i\partial _iu+\xi _i\partial _{ij}u\big ]=-\Delta (\xi \cdot \nabla u), \end{aligned}$$

and then we use the equation (7.12) for $w_R$.

**Step 3.** We next compute the second derivative of $\mathcal G(u_t,A_R)$ in terms of $u'$. For the sake of simplicity, through the rest of the proof we use the notations introduced in Sect. 1.5.

$$\begin{aligned} -\int _{\Omega _u\cap A_R}|\nabla w_R|^2\,dx=&\int _{A_R}-|\nabla (u'+\xi \cdot \nabla u)|^2\,dx\\ =&\int _{\Omega _u\cap A_R}-|\nabla u'|^2-2\nabla u'\cdot \nabla (\xi \cdot \nabla u)-|\nabla (\xi \cdot \nabla u)|^2\,dx\\ =&-\int _{\Omega _u\cap A_R}\Big (|\nabla u'|^2+|\nabla (\xi \cdot \nabla u)|^2\Big )\,dx\\&-2\int _{\partial (\Omega _u\cap A_R)}\frac{\partial u'}{\partial \nu } (\xi \cdot \nabla u)\,d\mathcal {H}^{d-1}\\ =&-\int _{\Omega _u\cap A_R}\Big (|\nabla u'|^2+|\nabla (\xi \cdot \nabla u)|^2\Big )\,dx\\&-2\int _{\partial (\Omega _u\cap A_R)}\frac{\partial u'}{\partial \nu } u'\,d\mathcal {H}^{d-1}\\ =&\int _{\Omega _u\cap A_R}\Big (|\nabla u'|^2-|\nabla (\xi \cdot \nabla u)|^2\Big )\,dx. \end{aligned}$$

On the other hand

$$\begin{aligned} \int _{\Omega _u\cap A_R}\nabla u\cdot (\delta ^2 A)\nabla u\,dx&=\int _{\Omega _u\cap A_R}|D\xi (\nabla u)|^2+\nabla u\cdot (D\xi )^2(\nabla u)\,dx\\&\quad -\int _{\Omega _u\cap A_R}\nabla u\cdot \Big ((\xi \cdot \nabla )[D\xi ]\Big )\nabla u\,dx\\&\quad -\int _{\Omega _u\cap A_R}2\,{{\,\textrm{div}\,}}\,\xi \,\nabla u\cdot D\xi \nabla u\,dx\\&\quad +\int _{\Omega _u\cap A_R}\frac{1}{2}|\nabla u|^2{{\,\textrm{div}\,}}\Big (\xi {{\,\textrm{div}\,}}\,\xi \Big )\,dx\\&=\int _{\Omega _u\cap A_R}|D\xi (\nabla u)|^2+\nabla u\cdot (D\xi )^2(\nabla u)\,dx\\&\quad -\int _{\Omega _u\cap A_R}\nabla u\cdot \Big ((\xi \cdot \nabla )[\nabla \xi ]\Big )\nabla u\,dx\\&\quad -\int _{\Omega _u\cap A_R}2\,{{\,\textrm{div}\,}}\,\xi \,\nabla u\cdot \nabla \xi \nabla u\,dx\\&\quad -\int _{\Omega _u\cap A_R}D^2 u(\nabla u)\cdot \xi ({{\,\textrm{div}\,}}\,\xi )\,dx\\&\quad +\int _{\partial \Omega _u\cap A_R}\frac{1}{2}|\nabla u|^2{{\,\textrm{div}\,}}\,\xi (\xi \cdot \nu )\,d\mathcal {H}^{d-1}. \end{aligned}$$

Notice that

$$\begin{aligned} -\nabla u\cdot \Big ((\xi \cdot \nabla )[\nabla \xi ]\Big )\nabla u&=-\partial _iu\,\xi _k\partial _{ki}\xi _j\,\partial _ju\\&=-\partial _k\Big [\partial _iu\,\xi _k\partial _{i}\xi _j\,\partial _ju\Big ]+\partial _{ki}u\,\xi _k\partial _{i}\xi _j\,\partial _ju\\&\qquad +\partial _iu\,\xi _k\partial _{i}\xi _j\,\partial _{kj}u+({{\,\textrm{div}\,}}\,\xi )\partial _iu\,\partial _{i}\xi _j\,\partial _ju \end{aligned}$$

and

$$\begin{aligned} -D^2 u(\nabla u)\cdot \xi ({{\,\textrm{div}\,}}\,\xi )&=-\partial _{ij}u\,\partial _ju\xi _i({{\,\textrm{div}\,}}\,\xi )\\&=-\partial _j\Big [\partial _{i}u\,\partial _ju\xi _i({{\,\textrm{div}\,}}\,\xi )\Big ]\\&\qquad +\partial _{i}u\,\partial _ju\partial _j\xi _i({{\,\textrm{div}\,}}\,\xi )+\partial _{i}u\,\partial _ju\xi _i\partial _j({{\,\textrm{div}\,}}\,\xi ), \end{aligned}$$

which leads to

$$\begin{aligned} -\nabla u\cdot \Big ((\xi \cdot \nabla )[\nabla \xi ]\Big )&\nabla u-2{{\,\textrm{div}\,}}\,\xi \,\nabla u\cdot \nabla \xi (\nabla u)-D^2 u(\nabla u)\cdot \xi ({{\,\textrm{div}\,}}\,\xi )\\&=-\partial _k\Big [\partial _iu\,\xi _k\partial _{i}\xi _j\,\partial _ju\Big ]-\partial _j\Big [\partial _{i}u\,\partial _ju\xi _i({{\,\textrm{div}\,}}\,\xi )\Big ]\\&\qquad +\partial _{ki}u\,\xi _k\partial _{i}\xi _j\,\partial _ju+\partial _iu\,\xi _k\partial _{i}\xi _j\,\partial _{kj}u+\xi _i\,\partial _{i}u\,\partial _ju\,\partial _j({{\,\textrm{div}\,}}\,\xi ). \end{aligned}$$

Similarly, we can compute

$$\begin{aligned} -|\nabla (\xi \cdot \nabla u)|^2=-\partial _k(\xi _i\partial _iu)\partial _k(\xi _j\partial _ju)&=-(\partial _k\xi _i\partial _iu+\xi _i\partial _{ki}u)(\partial _k\xi _j\partial _ju+\xi _j\partial _{kj}u)\\&=-|D\xi (\nabla u)|^2-\xi _i\partial _{ki}u\xi _j\partial _{kj}u-2\partial _k\xi _i\partial _iu\xi _j\partial _{kj}u\,, \end{aligned}$$

and so

$$\begin{aligned}&|\nabla \xi (\nabla u)|^2+\nabla u\cdot (\nabla \xi )^2(\nabla u)-|\nabla (\xi \cdot \nabla u)|^2\\&\quad =\partial _iu\,\partial _i\xi _j\partial _j\xi _k\partial _ku-2\partial _k\xi _i\partial _iu\xi _j\partial _{kj}u-\xi _i\partial _{ki}u\xi _j\partial _{kj}u\\&\quad =\partial _iu\,\partial _i\xi _j\partial _j\xi _k\partial _ku-2\partial _k\xi _i\partial _iu\xi _j\partial _{kj}u\\&\qquad -\partial _k\Big [\xi _i\partial _{i}u\xi _j\partial _{kj}u\Big ]+\partial _k\xi _i\partial _{i}u\xi _j\partial _{kj}u+\xi _i\partial _{i}u\partial _k\xi _j\partial _{kj}u\\&\quad =\partial _iu\,\partial _i\xi _j\partial _j\xi _k\partial _ku-2\partial _k\xi _i\partial _iu\xi _j\partial _{kj}u\\&\qquad -\partial _k\Big [\xi _i\partial _{i}u\xi _j\partial _{kj}u\Big ]+\partial _k\xi _i\partial _{i}u\xi _j\partial _{kj}u\\&\qquad +\partial _j\Big [\xi _i\partial _{i}u\partial _k\xi _j\partial _{k}u\Big ]-\partial _j\xi _i\partial _{i}u\partial _k\xi _j\partial _{k}u-\xi _i\partial _{ij}u\partial _k\xi _j\partial _{k}u-\xi _i\partial _{i}u\partial _k({{\,\textrm{div}\,}}\xi )\partial _{k}u. \end{aligned}$$

Finally, by collecting the previous computations, we obtain the identityThus, integrating by parts and using that $\nabla u= - \nu$ on $\partial \Omega _u\cap A_R$, we obtainwhere *H* is the mean curvature of $\partial \Omega _u\cap A_R$ oriented towards the complement of $\Omega _u$.

**Step 4. Conclusion.** Given any $\varphi \in C^\infty _c(\mathbb {R}^d{\setminus }\{0\})$, we consider an annulus $A_R$ containing the support of $\varphi$ and the vector field $\xi :=\varphi \nabla u$. Thus, by the minimality of $u'$ in $\Omega _u\cap A_R$, we get

$$\begin{aligned} \int _{\Omega _u}|\nabla \varphi |^2\,dx-\int _{\partial \Omega _u}\varphi ^2H\,d\mathcal {H}^{d-1}&\ge \int _{\Omega _u\cap A_R}|\nabla u'|^2\,dx-\int _{\partial \Omega _u\cap A_R}(u')^2H\,d\mathcal {H}^{d-1}\\&=\frac{1}{2}\frac{d^2}{dt^2}\Big |_{t=0}\mathcal G(u_t,A_R)\ge \frac{1}{2}\delta ^2\mathcal {G}(u)[\xi ]\ge 0, \end{aligned}$$

where the last inequality follows from (7.11). $\square$

## Hausdorff Dimension of $\text { Sing}(\partial \Omega )$

In this section we will estimate the dimension of the singular set $\text { Sing}(\partial \Omega )$ of a solution $\Omega$ to the shape optimization problem (1.3); our main result is the following.

### Theorem 8.1

(Dimension of the singular set) Let *D* be a bounded open set in $\mathbb {R}^d$ and let *f*, *g*, *Q* be as in Theorem 1.2, namely:

- $$f,g\in C^2(D)\cap L^\infty (D)$$;
- there are constants $C_1,C_2 > 0$ such that $0\le C_1 g\le f\le C_2 g$ in *D*;
- $Q\in C^{2}(D)$ and there are constants $c_Q,C_Q$ such that $0<c_Q\le Q\leqq C_Q$ on *D*.

Let $\Omega$ be a solution to (1.3) and let the singular part $\text { Sing}(\partial \Omega )$ of the free boundary $\partial \Omega \cap D$ be as in Sect. 5. Then, the following holds:

(i) If $$d< d^*$$, then $$\text { Sing}(\partial \Omega )=\emptyset $$.
(ii) If $d\ge d^*$, then the Hausdorff dimension of $\text { Sing}(\partial \Omega )$ is at most $d-d^*$, that is,
  $$\begin{aligned} \mathcal {H}^{d-d^*+{\varepsilon }}\big (\text { Sing}(\partial \Omega )\big )=0\quad \text {for every}\quad {\varepsilon }>0. \end{aligned}$$

In order to prove Theorem 8.1 above, we will first show that the stability of $\Omega$, expressed in terms of the state functions $u_\Omega$ and $v_\Omega$ as in Lemma 2.8, passes to a blow-up limit.

### Lemma 8.2

(Stability of the blow-up limits) Let *D* be a bounded open set in $\mathbb {R}^d$ and let *f*, *g*, *Q* be as in Theorems 1.2 and 8.1. Let $\Omega$ be an optimal set for (1.3) and let $u:=u_\Omega$ and $v:=v_\Omega$ be the state functions defined in (1.1) and (1.6). Suppose that the couple $u_0,v_0:\mathbb {R}^d\rightarrow \mathbb {R}$ is a blow-up limit of *u*, *v* at a point $x_0\in \partial \Omega \cap D$. Then,

8.1 $$\begin{aligned} \int _{\Omega _0}\Big (\nabla u_0\cdot (\delta ^2A)\nabla v_0-\nabla (\delta u_0)\cdot \nabla (\delta v_0) +Q(x_0)\frac{({{\,\textrm{div}\,}}\xi )^2+\xi \cdot \nabla ({{\,\textrm{div}\,}}\xi )}{2}\Big )\,dx\ge 0,\nonumber \\ \end{aligned}$$

where $\Omega _0:=\{u_0>0\}=\{v_0>0\}$, and $\delta u_0$ and $\delta v_0$ are the solutions respectively to the PDEs

8.2 $$\begin{aligned} \begin{aligned} -\Delta (\delta u_0)={{\,\textrm{div}\,}}\big ((\delta A)\nabla u_0\big )\quad \text {in }\Omega _0\ ,\qquad \delta u_0\in \dot{H}^1_0(\Omega _0)\,,\\ -\Delta (\delta v_0)={{\,\textrm{div}\,}}\big ((\delta A)\nabla u_{0}\big )\quad \text {in }\Omega _0\ ,\qquad \delta v_0\in \dot{H}^1_0(\Omega _0)\,, \end{aligned} \end{aligned}$$

in the sense explained in Sect. 7.1. In particular, if $u_0$ and $v_0$ are proportional, then there is a constant $\lambda >0$ such that $\lambda u_0$ is a global stable solution of the one-phase Bernoulli problem in the sense of Definition 7.3.

### Proof

Let $r_k\rightarrow 0$, be a sequence such that

$$\begin{aligned} u_k(x):=\frac{1}{r_k}u(x_0+r_kx)\qquad \text {and}\qquad v_k(x):=\frac{1}{r_k}v(x_0+r_kx), \end{aligned}$$

converge respectively to $u_0$ and $v_0$ locally uniformly and (by Proposition 4.3) strongly in $H^1_{loc}(\mathbb {R}^d)$. We define:

$$\begin{aligned} f_k(x):={r_k}f(x_0+r_kx),\quad g_k(x):={r_k}g(x_0+r_kx),\quad Q_{k}(x):=Q(x_0+r_kx). \end{aligned}$$

Then, the functions $u_k$ and $v_k$ satisfy the PDEs

$$\begin{aligned} -\Delta u_k=f_k\quad \text {in }\Omega _k\,\qquad u_k\in H^1_0(\Omega _k), \\ -\Delta v_k=g_k\quad \text {in }\Omega _k\,\qquad v_k\in H^1_0(\Omega _k), \end{aligned}$$

where

$$\begin{aligned} \Omega _k:=\frac{1}{r_k}(-x_0+\Omega ). \end{aligned}$$

Moreover, $\Omega _k$ is optimal in the rescaled domain

$$\begin{aligned} D_k:=\frac{1}{r_k}(-x_0+D), \end{aligned}$$

for the functional

$$\begin{aligned} \mathcal {F}_{k}(A):=\int _A\Big (\nabla u_A\cdot \nabla v_A-u_Ag_{k}-v_A f_{k}+Q_{k}\Big )\,dx\, \end{aligned}$$

where, this time, by $u_A$ and $v_A$ we denote the solutions to

$$\begin{aligned} -\Delta u_A=f_{k}\quad \text {in}\quad A\,\qquad u_A\in H^1_0(A), \\ -\Delta v_A=g_{k}\quad \text {in}\quad A\,\qquad v_A\in H^1_0(A). \end{aligned}$$

We fix a compactly supported smooth vector field $\xi \in C^\infty _c(\mathbb {R}^d;\mathbb {R}^d)$. Since $r_k\rightarrow 0$, for *k* large enough the support of $\xi$ is contained in $D_k$ and the stability of $\Omega _k$ (Lemma 2.8) reads as

8.3 $$\begin{aligned} \int _{\mathbb {R}^d}\Big (\nabla u_{k}\cdot (\delta ^2A)\nabla v_k-\nabla (\delta u_k)\cdot \nabla (\delta v_k)-(\delta ^2 f_k) v_k-(\delta ^2 g_k) u_k+\delta ^2Q_k\Big )\,dx\ge 0,\nonumber \\ \end{aligned}$$

where $\delta u_k$ and $\delta v_{k}$ are the solutions to

$$\begin{aligned} -\Delta (\delta u_k)={{\,\textrm{div}\,}}\big ((\delta A)\nabla u_k\big )+\delta f_k={{\,\textrm{div}\,}}\big ((\delta A)\nabla u_{k}+f_k\xi \big )\quad \text {in}\quad \Omega _k\,\qquad \delta u_k\in H^1_0(\Omega _k), \\ -\Delta (\delta v_{k})={{\,\textrm{div}\,}}\big ((\delta A)\nabla v_{k}\big )+\delta g_{k}={{\,\textrm{div}\,}}\big ((\delta A)\nabla v_{k}+g_k\xi \big )\quad \text {in}\quad \Omega _k\,\qquad \delta v_{k}\in H^1_0(\Omega _k), \end{aligned}$$

and where we used the following notation:

- $\delta A$ and $\delta ^2A$ are the matrices defined in (2.6) (we notice that $\delta A$ and $\delta ^2A$ are defined in terms of $\xi$ only);
- the variations $\delta f_{k}$ and $\delta ^2 f_{k}$ ($\delta g_{k}$ and $\delta ^2 g_{k}$ are defined analogously) are given by;
  $$\begin{aligned} \begin{aligned} \delta f_k(x)&:={{\,\textrm{div}\,}}(f_k\xi )=r_k^2\nabla f(x_0+r_kx)\cdot \xi +r_kf(x_0+r_kx)\,{{\,\textrm{div}\,}}\,\xi \,,\\ \delta ^2 f_k(x)&:= \frac{r_k^3}{2} \xi \cdot (D^2f(x_0+r_kx))\xi +\frac{r_k^2}{2}\nabla f(x_0+r_kx)\cdot D\xi [\xi ]\\&\quad +r_kf(x_0+r_kx)\frac{({{\,\textrm{div}\,}}\xi )^2+\xi \cdot \nabla [{{\,\textrm{div}\,}}\,\xi ]}{2}+r_k^2(\nabla f(x_0+r_kx) \cdot \xi ){{\,\textrm{div}\,}}\xi \,, \end{aligned} \end{aligned}$$
- $\delta ^2Q_k$ is given by
  $$\begin{aligned} \begin{aligned} \delta ^2Q_k(x)&:=(\xi \cdot \nabla Q_k){{\,\textrm{div}\,}}\xi +\frac{1}{2}\xi \cdot D^2 Q_k \xi +Q_k\frac{1}{2}\Big (({{\,\textrm{div}\,}}\xi )^2+\xi \cdot \nabla ({{\,\textrm{div}\,}}\xi )\Big )\\&=r_k(\xi \cdot \nabla Q(x+r_kx)){{\,\textrm{div}\,}}\xi +\frac{r_k^2}{2}\xi \cdot D^2 Q(x_0+r_kx) \xi \\&\qquad +\frac{1}{2}Q(x_0+r_k)\Big (({{\,\textrm{div}\,}}\xi )^2+\xi \cdot \nabla ({{\,\textrm{div}\,}}\xi )\Big ), \end{aligned} \end{aligned}$$
  where in all the formulas above, the field $\xi$ and its derivatives are all computed in *x*.

We notice that $\delta f_k$ and $\delta ^2f_k$ vanish outside the support of $\xi$. Moreover, since *f* and *g* are $C^2$, we get that $\delta f_k$ and $\delta ^2f_k$ converge to zero uniformly. Similarly, since *Q* is $C^2$, we get that

$$\begin{aligned} \delta ^2Q_k\rightarrow \frac{1}{2}Q(x_0)\Big (({{\,\textrm{div}\,}}\xi )^2+\xi \cdot \nabla ({{\,\textrm{div}\,}}\xi )\Big )\quad \text {strongly in}\quad L^1(\mathbb {R}^d). \end{aligned}$$

Next, we notice that by Proposition 4.3, $u_k$ and $v_k$ converge strongly in $H^1_{loc}(\mathbb {R}^d)$ to the blow-up limits $u_0$, $v_0$. Thus, since the support of $\delta ^2A$ is compact, we get

$$\begin{aligned} \lim _{k\rightarrow \infty }\int _{\mathbb {R}^d}\nabla u_k\cdot (\delta ^2A)\nabla v_k\,dx= \int _{\mathbb {R}^d}\nabla u_0\cdot (\delta ^2A)\nabla v_0\,dx. \end{aligned}$$

Similarly, we have that

$$\begin{aligned} (\delta A)\nabla u_{k}+f_k\xi \rightarrow (\delta A)\nabla u_0\qquad \text {and}\qquad (\delta A)\nabla v_{k}+g_k\xi \rightarrow (\delta A)\nabla v_0, \end{aligned}$$

strongly in $L^2(\mathbb {R}^d;\mathbb {R}^d)$. Thus, by Lemma 7.2, we get that $\delta u_k$ and $\delta v_k$ converge respectively to the solutions $\delta u_0$ and $\delta v_0$ of (8.2). Now, (8.1) follows by passing to the limit (8.3). $\square$

### Proof of Theorem 8.1

The strategy is similar to the one for minimizers of the one-phase problem. We split the proof in two different cases.

Case 1: $d<d^*$. Let $x_0 \in \partial \Omega _u\cap D$ and $r_k \rightarrow 0^+$ be the infinitesimal sequence such that the rescalings $u_{x_0,r_k}$ and $v_{x_0,r_k}$ converge to the 1-homogeneous blow-up limits $u_0$ and $v_0$ given by Proposition 4.7. Then, given $\lambda \in (C_1,C_2)$ as in Proposition 4.7, the blow-up limits

8.4 $$\begin{aligned} u_0:=\lim _{k\rightarrow \infty }\frac{1}{ {\sqrt{\lambda } Q(x_0)}}u_{x_0,r_k}\qquad \text {and}\qquad v_0:=\lim _{k\rightarrow \infty }\sqrt{\lambda }v_{x_0,r_k}\,\end{aligned}$$

coincide up to a multiplicative constant (that is $v_0=Q(x_0)v_0$) and, by Lemma 8.2 and Proposition 4.7, $u_0$ is a 1-homogeneous stable solutions of the one-phase problem in the sense of Definition 7.3. Therefore, by definition of $d^*$, $\text { Sing}(\partial \Omega _{u_0})=\emptyset$ and $u_0$ is a half space solution:

$$\begin{aligned} u_0(x)=(x\cdot \nu )_+\quad \text {for some unit vector}\quad \nu \in \mathbb {R}^d. \end{aligned}$$

Finally, by rewriting the result in terms of the blow-up sequence of Proposition 4.7, we deduce the existence $\alpha >0$ and $\beta >0$ such that $\alpha \beta =Q(x_0)$ such that

$$\begin{aligned} \frac{1}{r_k}u(x_0+r_k x) \rightarrow \alpha (x\cdot \nu )_+,\quad \text{ and }\quad \frac{1}{r_k}v(x_0+r_k x) \rightarrow \beta (x\cdot \nu )_+ \end{aligned}$$

as $k\rightarrow \infty$. Thus, by definition, $x_0 \in \text { Reg}(\partial \Omega )$. Since this is true at every free boundary point $x_0\in \partial \Omega \cap D$, we get that $\text { Sing}(\partial \Omega )=\emptyset$.

Case 2: $d\geqq d^*$. Given ${\varepsilon }>0$, let us prove that

$$\begin{aligned} \mathcal {H}^{d-d^*+{\varepsilon }}\big (\text { Sing}(\partial \Omega )\big )=0. \end{aligned}$$

We will apply consecutively the three blow-ups from Sect. 4. By contradiction, assume that

$$\begin{aligned} \mathcal {H}^{d-d^*+{\varepsilon }}\big (\text { Sing}(\partial \Omega )\big )\ge C>0. \end{aligned}$$

Therefore, by [37, Lemma 10.5], there are $x_0 \in \text { Sing}(\partial \Omega )$ and a sequence $r_k\rightarrow 0$ such that

$$\begin{aligned} \mathcal {H}^{d-d^*+{\varepsilon }}\big ( \text{ Sing }(\partial \Omega )\cap B_{r_k}(x_0)\big )\ge C r_k^{d-d^*+{\varepsilon }}. \end{aligned}$$

By the first blow-up analysis from Sect. 4.2 (see Proposition 4.3), we deduce that the blow-up sequences $u_{x_0,r_k},v_{x_0,r_k}$ and $\Omega _{x_0,r_k}$ converge to some limits $u_0, v_0$ and $\Omega _0$ such that

$$\begin{aligned} \mathcal {H}^{d-d^*+{\varepsilon }}\big ( \text{ Sing }(\partial \Omega _0)\cap B_{1}\big )\ge C, \end{aligned}$$

where $u_0$, $v_0$ and $\Omega _0$ are as in Proposition 4.3 and where $u_0$ and $v_0$ satisfy the stability condition (8.1) from Lemma 8.2. By applying again [37, Lemma 10.5], there exists a point $x_{00} \in \text { Sing}(\partial \Omega _0)$ and another sequence $r_k\rightarrow 0^+$ such that

$$\begin{aligned} \mathcal {H}^{d-d^*+{\varepsilon }}\big ( \text{ Sing }(\partial \Omega _0)\cap B_{r_k}(x_{00})\big )\ge C r_k^{d-d^*+{\varepsilon }}. \end{aligned}$$

Hence, by applying the second blow-up analysis of Sect. 4.3 to the sequences

$$\begin{aligned} \frac{1}{r_k\sqrt{\lambda }\,Q(x_0)}u_0(x_{00}+r_kx),\qquad \frac{\sqrt{\lambda }}{r_k}v_0(x_{00}+r_kx)\quad \text{ and }\quad \frac{1}{r_k}(\Omega _0-x_{00}), \end{aligned}$$

we get that

$$\begin{aligned} \mathcal {H}^{d-d^*+{\varepsilon }}\big ( \text{ Sing }(\partial \Omega _{00})\cap B_{1}\big )\ge C, \end{aligned}$$

where $v_{00}=Q(x_0) u_{00}$ and $u_{00}$ satisfies (2)-(3)-(4)-(5)-(6) in Lemma 4.4. Moreover, by the strong convergence of the blow-up sequence, $u_{00}$ and $v_{00}$ still satisfy (8.1), so $u_{00}$ is a global stable solution of the one-phase problem in the sense of Definition 7.3. Finally, by applying for the last time [37, Lemma 10.5], we deduce that

8.5 $$\begin{aligned} \mathcal {H}^{d-d^*+{\varepsilon }}\big ( \text{ Sing }(\partial \Omega _{000})\cap B_{1}\big )\ge C, \end{aligned}$$

in which $\Omega _{000}=\{u_{000}>0\}$ and $u_{000}$ is a blow-up limit of $u_{00}$ at some free boundary point $x_{000}\in \partial \Omega _{00}$. But now $u_{000}$ is a 1-homogeneous stable solution of the one-phase problem in the sense of Definition 7.3, so (8.5) contradicts Theorem 7.8. $\square$

## Existence of Optimal Sets in $\mathbb {R}^d$ and Proof of Theorem 1.6

In this section we prove Theorem 1.6; we prove the existence of an optimal set for (1.4) and then we show how to obtain the regularity of the optimal sets as a consequence from Theorem 1.2.

### Statement of the Problem in the Class of Measurable Sets

One can not obtain the existence of optimal sets (for (1.4) or (1.3)) directly in the class of open sets. Thus, we extend the definition of the shape functional to the class of measurable sets. Precisely, if $\Omega$ is a Lebesgue measurable set of finite measure in $\mathbb {R}^d$, we define the space ${\widetilde{H}}^1_0(\Omega )$ of all $H^1(\mathbb {R}^d)$ functions vanishing (Lebesgue-)almost-everywhere outside $\Omega$. We will say that *u* is a (weak) solution to the problem

9.1 $$\begin{aligned} -\Delta u=f\quad \text {in }\Omega ,\qquad u\in {\widetilde{H}}^1_0(\Omega ), \end{aligned}$$

if $$u\in {\widetilde{H}}^1_0(\Omega )$$ and

9.2 $$\begin{aligned} \int _\Omega \nabla v\cdot \nabla u\,dx=\int _\Omega vf\,dx\quad \text {for every}\quad v\in {\widetilde{H}}^1_0(\Omega ). \end{aligned}$$

It is immediate to check that *u* satisfies (9.2) if and only if

9.3 $$\begin{aligned}  &   \frac{1}{2}\int _{\mathbb {R}^d}|\nabla u|^2\,dx-\int _\Omega uf(x)\,dx\le \frac{1}{2}\int _{\mathbb {R}^d}|\nabla v|^2\,dx\nonumber \\  &   -\int _\Omega vf(x)\,dx\quad \text {for every }v\in {\widetilde{H}}^1_0(\Omega ). \end{aligned}$$

From now on, we will denote the unique solution to (9.1) by ${\widetilde{u}}_\Omega$.

We will first prove the following lemma, which in particular implies that for every open set $\Omega \subset \mathbb {R}^d$ of finite measure and non-negative functions $f,g\in L^2(\Omega )$, we have

$$\begin{aligned} \int _\Omega \Big (-g(x){\widetilde{u}}_\Omega +Q(x)\Big )\,dx\le \int _\Omega \Big (-g(x) u_\Omega +Q(x)\Big )\,dx, \end{aligned}$$

where $u_\Omega$ is the weak solution to (1.1).

#### Lemma 9.1

Let $\Omega$ be a bounded open set in $\mathbb {R}^d$ and $f\in L^2(\Omega )$ a non-negative function. Then

9.4 $$\begin{aligned} 0\le u_\Omega \le {\widetilde{u}}_\Omega , \end{aligned}$$

where ${\widetilde{u}}_\Omega \in {\widetilde{H}}^1_0(\Omega )$ is the solution to (9.1) and $u_\Omega \in H^1_0(\Omega )$ is the weak solution to (1.1).

Moreover, if $\Omega$ satisfies the following exterior density estimate:

9.5 $$\begin{aligned} \begin{array}{ll} \text {there are constants } r_0>0 \text { and } c>0 \text { such that }\\ \qquad \text {for every } x_0\in \partial \Omega \text { and every } r\le r_0 \quad |B_r(x_0)\setminus \Omega |\ge c|B_r|, \end{array} \end{aligned}$$

then $$H^1_0(\Omega )={\widetilde{H}}^1_0(\Omega )$$ and $$ u_\Omega ={\widetilde{u}}_\Omega $$.

#### Proof

First we notice that $u_\Omega$ and ${\widetilde{u}}_\Omega$ are the unique minimizers of the functional (9.3) in $H^1_0(\Omega )$ and ${\widetilde{H}}^1_0(\Omega )$. Since $f\ge 0$, by testing the optimality of $u_\Omega$ with $u_\Omega \vee 0\in H^1_0(\Omega )$ and the optimality of ${\widetilde{u}}_\Omega$ with ${\widetilde{u}}_\Omega \vee 0\in \widetilde{H}^1_0(\Omega )$, we get that $u_\Omega \ge 0$ and ${\widetilde{u}}_\Omega \ge 0$ in $\Omega$. Now, a standard argument (see for instance [38, Lemma 2.6]) gives that $\Delta u_\Omega +f\ge 0$ on $\mathbb {R}^d$ in the sense of distributions, precisely:

9.6 $$\begin{aligned} -\int _{\mathbb {R}^d}\nabla u_\Omega \cdot \nabla \varphi \,dx+\int _{\mathbb {R}^d}\varphi f\,dx\ge 0\quad \text {for every }\varphi \ge 0,\ \varphi \in H^1(\mathbb {R}^d). \end{aligned}$$

Next, we notice that for every open set $\Omega$, we have the inclusion $H^1_0(\Omega )\subset {\widetilde{H}}^1_0(\Omega )$. Thus, if we take $\varphi$ to be the negative part of ${\widetilde{u}}_\Omega -u_\Omega$,

$$\begin{aligned} \varphi :=-\Big (({\widetilde{u}}_\Omega -u_\Omega )\wedge 0\Big ), \end{aligned}$$

we have that $\varphi \in {\widetilde{H}}^1_0(\Omega )$. So, using the (weak) equation for ${\widetilde{u}}_\Omega$, we get

$$\begin{aligned} \int _{\mathbb {R}^d}\nabla \varphi \cdot \nabla {\widetilde{u}}_\Omega \,dx=\int _\Omega \varphi \,f(x)\,dx \end{aligned}$$

On the other hand, by the positivity of $\varphi$ and (9.6),

$$\begin{aligned} \int _{\mathbb {R}^d}\nabla \varphi \cdot \nabla u_\Omega \,dx\le \int _\Omega \varphi \,f(x)\,dx. \end{aligned}$$

Combining the two, we obtain

$$\begin{aligned} \int _{\mathbb {R}^d}|\nabla \varphi |^2\,dx=-\int _{\mathbb {R}^d}\nabla \varphi \cdot \nabla ({\widetilde{u}}_\Omega -u_\Omega )\,dx\le 0, \end{aligned}$$

which gives that $\varphi \equiv 0$ and so (9.4) holds. Finally, it is known (see for instance [17, Proposition 4.7]) that in the presence of the density estimate (9.5), we have that $H^1_0(\Omega )={\widetilde{H}}^1_0(\Omega )$. Thus, the equality $u_\Omega ={\widetilde{u}}_\Omega$ follows by the fact that the minimizer of the functional in (9.3) is unique. $\square$

### Existence of Optimal Measurable Sets in $\mathbb {R}^d$

For all measurable set $\Omega \subset \mathbb {R}^d$, we set

$$\begin{aligned} {\widetilde{J}}(\Omega ):=\int _\Omega -g(x)u_\Omega +Q(x)\,dx. \end{aligned}$$

We can prove the following existence result for the minimization of ${\widetilde{J}}$.

#### Lemma 9.2

If $d\ge 3$, let $f,g\in L^2(\mathbb {R}^d)$, while if $d=2$, let $f,g\in L^1(\mathbb {R}^2)\cap L^\infty (\mathbb {R}^2)$ be nonnegative functions, and let $Q:\mathbb {R}^d\rightarrow \mathbb {R}$ be a measurable function bounded from below by a positive constant $c_Q>0$. Then, the following variational problem has a solution

9.7 $$\begin{aligned} \text{ min }\bigg \{\int _\Omega \Big (-g(x){\widetilde{u}}_\Omega +Q(x)\Big )\,dx\ :\ \Omega \subset \mathbb {R}^d,\ \Omega \ \text{ measurable },\ |\Omega |<+\infty \bigg \}.\nonumber \\ \end{aligned}$$

#### Proof

Let $d\ge 3$, let $\Omega _n$ be a minimizing sequence of sets of finite Lebesgue measure for (9.7). We set $u_n:={\widetilde{u}}_{\Omega _n}$. By the Poincaré inequality, the equation for $u_n$ and the Hölder inequality, there is a dimensional constant $C_d$ such that

$$\begin{aligned}  &   \Vert u_n\Vert _{L^2(\Omega _n)}^2\le C_d|\Omega _n|^{{2}/{d}}\int _{\Omega _n}|\nabla u_n|^2\,dx\\  &   =C_d|\Omega _n|^{{2}/{d}}\int _{\Omega _n}u_nf\,dx\le C_d|\Omega _n|^{{2}/{d}}\Vert f\Vert _{L^2(\mathbb {R}^d)}\Vert u_n\Vert _{L^2(\mathbb {R}^d)}. \end{aligned}$$

Thus,

$$\begin{aligned} \Vert u_n\Vert _{L^2(\Omega _n)}\le C_d|\Omega _n|^{{2}/{d}}\Vert f\Vert _{L^2(\mathbb {R}^d)}, \end{aligned}$$

and so (since we can suppose that ${\widetilde{J}}(\Omega _n)\le {\widetilde{J}}(\emptyset )=0$), we get

$$\begin{aligned}  &   0\ge {\widetilde{J}}(\Omega _n)=\int _{\Omega _n}\Big (-g(x)u_n+Q(x)\Big )\,dx\\  &   \ge -C_d|\Omega _n|^{{2}/{d}}\Vert f\Vert _{L^2(\mathbb {R}^d)}\Vert g\Vert _{L^2(\mathbb {R}^d)}+c_Q|\Omega _n|, \end{aligned}$$

which implies that the sequence of measures $|\Omega _n|$ is bounded

9.8 $$\begin{aligned} |\Omega _n|^{\frac{d-2}{d}}\le \frac{C_d}{c_Q}\Vert f\Vert _{L^2(\mathbb {R}^d)}\Vert g\Vert _{L^2(\mathbb {R}^d)}. \end{aligned}$$

If $d=2$, to obtain a bound on the measure analogous to (9.8), we need to use a generalized Poincaré-Sobolev (or generalized Faber–Krahn) inequality (see for example [3, equation (1.2)]) instead of the classical one, namely, for $2<q<+\infty$, there exists a dimensional constant ${\widetilde{C}}_{2}$ such that (using also Hölder inequality)

$$\begin{aligned}\begin{aligned} \Vert u_n\Vert ^2_{L^q(\Omega _n)}&\le {\widetilde{C}}_{2}|\Omega _n|^{1+\tfrac{2-q}{q}}\int _{\Omega _n}|\nabla u_n|^2\,dx={\widetilde{C}}_{2}|\Omega _n|^{1+\tfrac{2-q}{q}}\int _{\Omega _n}u_nf\,dx\\&\le \widetilde{C}_{2}|\Omega _n|^{1+\tfrac{2-q}{q}}\Vert f\Vert _{L^{q'}(\mathbb {R}^2)}\Vert u_n\Vert _{L^q(\Omega _n)}. \end{aligned}\end{aligned}$$

We immediately deduce that

9.9 $$\begin{aligned} \Vert u_n\Vert _{L^q(\Omega _n)}\leqq \widetilde{C}_{2}|\Omega _n|^{1+\tfrac{2-q}{q}}\Vert f\Vert _{L^{q'}(\mathbb {R}^2)}, \end{aligned}$$

and again supposing that ${\widetilde{J}}(\Omega _n)\le \widetilde{J}(\emptyset )=0$, we obtain, using (9.9)

$$\begin{aligned}  &   0\ge {\widetilde{J}}(\Omega _n)=\int _{\Omega _n}\Big (-g(x)u_n+Q(x)\Big )\,dx\\  &   \ge -{\widetilde{C}}_2|\Omega _n|^{1+\tfrac{2-q}{q}}\Vert f\Vert _{L^{q'}(\mathbb {R}^2)}\Vert g\Vert _{L^{q'}(\mathbb {R}^2)}+c_Q|\Omega _n|, \end{aligned}$$

which, since $q>2$ implies that the sequence of measures $|\Omega _n|$ is bounded, namely

$$\begin{aligned} |\Omega _n|^{\frac{q-2}{q}}\le \frac{\widetilde{C}_2}{c_Q}\Vert f\Vert _{L^{q'}(\mathbb {R}^2)}\Vert g\Vert _{L^{q'}(\mathbb {R}^2)}. \end{aligned}$$

Now the proof continues in the same way both for $d=2$ and $d\geqq 3$. Using again the equation for $u_n$ we get that the sequence $u_n$ is bounded in $H^1(\mathbb {R}^d)$. Thus, up to pass to a subsequence, we have that $u_n$ converges weakly in $H^1_{\textrm{loc}}(\mathbb {R}^d)$ and strongly in $L^2_{\textrm{loc}}(\mathbb {R}^d)$ (and, up to another subsequence, also pointwise a.e. in $\mathbb {R}^d$) to a certain $u\in H^1_{\textrm{loc}}(\mathbb {R}^d).$ Now, for all $R>0$

$$\begin{aligned} |\{u\ne 0\}\cap B_R|\le \liminf _{n\rightarrow \infty }|\{u_n\ne 0\}\cap B_R|\le \liminf _{n\rightarrow \infty }|\Omega _n\cap B_R|\le \liminf _{n\rightarrow \infty }|\Omega _n|. \end{aligned}$$

Taking a supremum over $R>0$, we obtain that

$$\begin{aligned} |\{u\ne 0\}|\leqq \liminf _{n\rightarrow \infty }|\Omega _n|, \end{aligned}$$

and as a consequence (using Fatou Lemma),

$$\begin{aligned} \int _{\{u\ne 0\}}Q(x)\,dx\leqq \liminf _{n\rightarrow +\infty }\int _{\Omega _n}Q(x)\,dx, \end{aligned}$$

Then one can prove also that $u\in H^1(\mathbb {R}^d)\cap \widetilde{H}^1_0(\{u\ne 0\})$, as, for all $R>0$,

$$\begin{aligned} \int _{B_R}|\nabla u|^2\,dx\leqq \liminf _{n\rightarrow +\infty }\int _{B_R\cap \Omega _n}|\nabla u_n|^2\,dx\leqq \liminf _{n\rightarrow +\infty }\int _{\Omega _n}|\nabla u_n|^2\,dx, \end{aligned}$$

and then we can take the supremum over $R>0$. We note that from the pointwise a.e. convergence and the fact that $u_n\geqq 0$ (since $f\geqq 0$), we deduce $u\geqq 0$. As a consequence, using also that $g\geqq 0$, for all $R>0$ we have

$$\begin{aligned} -\int _{\{u\ne 0\}}gu\,dx\leqq -\int _{\{u\ne 0\}\cap B_R}gu\,dx\leqq \liminf _{n\rightarrow +\infty }-\int _{\Omega _n\cap B_R}gu_n\,dx. \end{aligned}$$

We then conclude that $\Omega =\{u\ne 0\}$ is a solution to (9.7). $\square$

#### Lemma 9.3

Let $d\ge 2$, $f,g\in L^1(\mathbb {R}^d)\cap L^\infty (\mathbb {R}^d)$ be non-negative, and let $Q:\mathbb {R}^d\rightarrow \mathbb {R}$ be a measurable function bounded from below by a positive constant $c_Q>0$. If the measurable set $\Omega \subset \mathbb {R}^d$, $|\Omega |<+\infty$, is a solution to (9.7), then $\Omega$ is bounded.

#### Proof

For any set $E\subset \mathbb {R}^d$ of finite measure and any function $h\in L^2(E)$, we will denote by ${\widetilde{R}}_E(h)$ the unique solution to the problem

$$\begin{aligned} -\Delta u=h\quad \text {in}\quad E\,\qquad u\in \widetilde{H}^1_0(E). \end{aligned}$$

We have that ${\widetilde{R}}_E$ is linear, $\widetilde{R}_E(h_1+h_2)={\widetilde{R}}_E(h_1)+{\widetilde{R}}_E(h_2)$ and positive: if $h\ge 0$, then ${\widetilde{R}}_E(h)\ge 0$. Moreover, by the weak maximum principle, if $E_1\subset E_2$ and $h\ge 0$, then ${\widetilde{R}}_{E_2}(h)\ge {\widetilde{R}}_{E_1}(h).$

Let $\omega$ be any measurable set contained in $\Omega$. Then, the minimality of $\Omega$ gives that

$$\begin{aligned} \int _\Omega \Big (-g(x){\widetilde{R}}_\Omega (f)+Q(x)\Big )\,dx\le \int _\omega \Big (-g(x){\widetilde{R}}_\omega (f)+Q(x)\Big )\,dx. \end{aligned}$$

So, rearranging the terms and using the positivity of *f* and *g*, and the inequality

$$\begin{aligned} 0\le {\widetilde{R}}_\Omega (f)-{\widetilde{R}}_\omega (f)\le \Vert f\Vert _{L^\infty }\Big ({\widetilde{R}}_\Omega (1)-\widetilde{R}_\omega (1)\Big ), \end{aligned}$$

we get

$$\begin{aligned} c_Q|\Omega \setminus \omega |&\le \int _\Omega Q(x)\,dx-\int _\omega Q(x)\,dx\le \int _\Omega g(x)\Big ({\widetilde{R}}_\Omega (f)-{\widetilde{R}}_\omega (f)\Big )\,dx\\&\le \Vert f\Vert _{L^\infty }\int _\Omega g(x)\Big ({\widetilde{R}}_\Omega (1)-{\widetilde{R}}_\omega (1)\Big )\,dx= \Vert f\Vert _{L^\infty }\int _\Omega \Big ({\widetilde{R}}_\Omega (g)-{\widetilde{R}}_\omega (g)\Big )\,dx\\&\le \Vert f\Vert _{L^\infty }\Vert g\Vert _{L^\infty }\int _\Omega \Big ({\widetilde{R}}_\Omega (1)-{\widetilde{R}}_\omega (1)\Big )\,dx\,. \end{aligned}$$

Finally, rearranging the terms again, we get that

$$\begin{aligned} -\frac{1}{2}\int _\Omega \widetilde{R}_\Omega (1)\,dx+\frac{c_Q}{2\Vert f\Vert _{L^\infty }\Vert g\Vert _{L^\infty }}|\Omega |\le -\frac{1}{2}\int _\omega \widetilde{R}_\omega (1)\,dx+\frac{c_Q}{2\Vert f\Vert _{L^\infty }\Vert g\Vert _{L^\infty }}|\omega |, \end{aligned}$$

so the set $\Omega$ is inwards minimizing (or, in terms of [9], a shape subsolution) for the functional

$$\begin{aligned} \mathcal E(\Omega )=-\frac{1}{2}\int _\Omega {\widetilde{R}}_\Omega (1)\,dx+\frac{c_Q}{2\Vert f\Vert _{L^\infty }\Vert g\Vert _{L^\infty }}|\Omega |. \end{aligned}$$

Thus, applying [9, Theorem 3.13], for every

$$\begin{aligned} 0<\eta \le \frac{c_Q}{2\Vert f\Vert _{L^\infty }\Vert g\Vert _{L^\infty }}, \end{aligned}$$

the set $\Omega$ is contained in an open set $A\subset \mathbb {R}^d$ obtained as a finite union of *N* balls $B_\rho (x_i)$, $i=1,\dots ,N$, with *N* and $\rho$ depending only on the dimension *d* and $\eta$. In particular, *A* is bounded and the diameter of any connected component of *A* is at most $N\rho$. $\square$

### Proof of Theorem 1.6

The existence of an optimal set is a consequence of Proposition 9.4 below, while the regularity follows from Theorem 1.2.

#### Proposition 9.4

In $\mathbb {R}^d$, $d\ge 2$, let $f,g,Q:\mathbb {R}^d\rightarrow \mathbb {R}$ be non-negative functions. Suppose that:

- $f,g\in L^\infty (\mathbb {R}^d)\cap L^1(\mathbb {R}^d)\cap C(\mathbb {R}^d)$ and that $f>0$ and $g>0$ on $\mathbb {R}^d$ ;
- there is a constant $c_Q>0$ such that $c_Q\le Q$ on $\mathbb {R}^d$.

Then, there exists a solution $\Omega \subset \mathbb {R}^d$ to the shape optimization problem (1.4). Moreover, every solution to (1.4) is also a solution to (9.7).

#### Proof

By Lemma 9.2, there is a measurable set $\Omega$ (of finite measure) that minimizes (9.7). By Lemma 9.3, $\Omega$ is contained in some ball $B_R\subset \mathbb {R}^d$. Since *f* and *g* are continuous and strictly positive on ${\overline{B}}_R$, we can find positive constants $C_1,C_2$ such that $C_1g\le f\le C_2g$ on ${\overline{B}}_R$. Thus, reasoning as in Proposition 3.1 and Corollary 3.3 we get that the set

$$\begin{aligned} A:=\{{\widetilde{u}}_\Omega >0\} \end{aligned}$$

is open. Now, the optimality of $\Omega$ gives that $|\Omega \Delta A|=0$, so we have ${\widetilde{u}}_A={\widetilde{u}}_\Omega .$ Moreover, by Proposition 3.5, *A* satisfies the exterior density estimate from Lemma 9.1. Thus, $u_A={\widetilde{u}}_A$. In order to check that the open set *A* minimizes (1.4), we notice that, for any open set $E\subset \mathbb {R}^d$,

$$\begin{aligned} \int _A\Big (-g(x) u_A+Q(x)\Big )\,dx&=\int _A\Big (-g(x) {\widetilde{u}}_A+Q(x)\Big )\,dx\\&\le \int _E\Big (-g(x) {\widetilde{u}}_E+Q(x)\Big )\,dx\\&\le \int _E\Big (-g(x) u_E+Q(x)\Big )\,dx\,. \end{aligned}$$

Moreover, by the same chain of inequalities, we obtain that if the open set *E* is a solution to (1.4), then it also minimizes (9.7). $\square$

## Data Availability

Data sharing is not applicable to this article as no datasets were generated or analysed during the current study.

## Appendix A. An Optimization Problem in Heat Conduction

As a consequence of our analysis from Sects. 2, 4 and 7, we obtain an estimate on the dimension of the singular part of the boundary of the optimal sets arising in a heat conduction problem studied by Aguilera, Caffarelli and Spruck in [1].

### A.1 Statement of the Problem and Known Results

Let *D* be a smooth bounded open set in $\mathbb {R}^d$. Let $\phi :\partial D\rightarrow \mathbb {R}$ be a smooth function on $\partial D$ bounded from below and above by positive constants $0<c\le C$. For every open set $\Omega \subset D$, for which the set $K:= D\setminus \Omega$ is a compact subset of *D*, we consider the state function $u_\Omega \in H^1(D)$ solution to the problem

A.1 $$\begin{aligned} \Delta u_\Omega =0\quad \text {in }\Omega ,\qquad u_\Omega =\phi \quad \text {on }\partial D,\qquad u_\Omega \equiv 0\quad \text {on }K=D\setminus \Omega , \end{aligned}$$

and we define the functional

$$\begin{aligned} \mathcal {F}(\Omega )=\int _{\partial D}\frac{\partial u_\Omega }{\partial \nu }\,d\mathcal {H}^{d-1}+\Lambda |\Omega |\, \end{aligned}$$

where $\Lambda >0$ is a real number and $\nu$ is the outer unit normal to $\partial D$. In [1] it was shown that there is a solution to the following shape optimization problem:

A.2 $$\begin{aligned} \text {min}\Big \{\mathcal {F}(\Omega )\,\ \Omega \subset D;\ \Omega \,-\,\text {open};\ D\setminus \Omega \,-\,\text {compact subset of\ } D\Big \}, \end{aligned}$$

and that for every solution $\Omega$ to (A.2) the following holds.

- The state function $u_\Omega$ is Lipschitz continuous in *D* and smooth in a neighborhood of the fixed boundary $\partial D$.
- $u_\Omega$ is non-degenerate in the sense that there is a constant $\eta >0$, for which
  $$\begin{aligned} \frac{1}{r^{d-1}}\int _{\partial B_r(x)}u_\Omega \,d\mathcal {H}^{d-1}\ge \eta \,r\quad \text {whenever}\quad x\in {\overline{\Omega }}\quad \text {and}\quad B_r(x)\subset D. \end{aligned}$$
- There is ${\varepsilon }>0$ such that, for every *x* on the boundary of the set $K:=D\setminus \Omega$, we have:
  $$\begin{aligned} {\varepsilon }_0|B_r|\le |\Omega \cap B_r(x)|\le (1-{\varepsilon }_0)|B_r|\quad \text {whenever}\quad B_r(x)\subset D. \end{aligned}$$
- There is a constant $\tilde{M}>0$ such that, for every $B_{2r}(x_0)\subset D$, we have the bound
  $$\begin{aligned} 0\le \bigg |\int _{B_{2r}(x_0)}\nabla u_\Omega \cdot \nabla \varphi \,dx\bigg |\le \tilde{M} \Vert \varphi \Vert _{L^\infty (B_{2r}(x_0))}\quad \text {for every}\quad \varphi \in C^{0,1}_c(B_{2r}(x_0)), \end{aligned}$$
  where $C^{0,1}_c(B_{2r}(x_0))$ is the space of Lipschitz functions with compact support in $B_{2r}(x_0)$.

Then, they showed that the reduced boundary $\partial ^*\Omega \cap D$ is $C^\infty$ smooth and that

A.3 $$\begin{aligned} |\nabla u_\Omega ||\nabla v_\Omega |=\Lambda \quad \text {on }\partial ^*\Omega \cap D, \end{aligned}$$

where $v_\Omega$ is the solution to the PDE

A.4 $$\begin{aligned} \Delta v_\Omega =0\quad \text {in }\Omega ,\qquad v_\Omega =1\quad \text {on }\partial D,\qquad v_\Omega \equiv 0\quad \text {on }K=D\setminus \Omega . \end{aligned}$$

In Theorem A.4, we will improve this result in low dimension ($d\ge 4$) by showing that the whole free boundary $\partial \Omega \cap D$ is smooth.

#### Remark A.1

In the problem originally considered by Aguilera, Caffarelli and Spruck in [1], the minimization of $\mathcal {F}$ is among all domains with prescribed measure. Thus, all the results from [1] apply to (A.2) with the only difference that the constant $\Lambda$ from (A.3) is an unknown positive Lagrange multiplier. In this last section of the present paper, we choose to work with the penalized version (A.2) since it allows to apply all the results from Sects. 2, 7 and 8 directly, without the need to add technical details related to the Lagrange multiplier.

### A.2 First and Second Variation

By an integration by parts, we can rewrite $\mathcal {F}(\Omega )$ as

$$\begin{aligned} \mathcal {F}(\Omega ):=\int _\Omega \nabla u_\Omega \cdot \nabla v_\Omega \,dx+\Lambda |\Omega |, \end{aligned}$$

where $u_\Omega$ and $v_\Omega$ are given by (A.1) and (A.4). Now, let $\xi \in C^\infty _c(D;\mathbb {R}^d)$ be a smooth compactly supported vector field in *D*; let $\Phi _t:D\rightarrow D$, $t\in \mathbb {R}$, be the flow associated to $\xi$ and let $\Omega _t:=\Phi _t(\Omega )$. Reasoning as in Sect. 7, we get that

$$\begin{aligned} \begin{aligned} \frac{\partial }{\partial t}\bigg |_{t=0}\mathcal {F}({\Omega _t})&=\,\int _\Omega \Big (\nabla u_\Omega \cdot (\delta A)\nabla v_\Omega +\Lambda {{\,\textrm{div}\,}}\xi \Big ) \, dx\,,\\ \frac{1}{2}\frac{\partial ^2}{\partial t^2}\bigg |_{t=0}\mathcal {F}({\Omega _t})&=\, \int _\Omega \Big (\nabla u_\Omega \cdot (\delta ^2 A)\nabla v_\Omega -\nabla (\delta u)\cdot \nabla (\delta v)+\frac{\Lambda }{2}\big (({{\,\textrm{div}\,}}\xi )^2+\xi \cdot \nabla ({{\,\textrm{div}\,}}\,\xi )\big )\Big ) dx\,, \end{aligned} \end{aligned}$$

where $\delta A$ and $\delta ^2A$ are given by (2.6), and where $\delta u$ and $\delta v$ are the solutions to the PDEs

$$\begin{aligned} \begin{aligned} -\Delta (\delta u)={{\,\textrm{div}\,}}\big ((\delta A)\nabla u_\Omega \big )\quad \text {in }\Omega \ ,\qquad \delta u\in H^1_0(\Omega )\,;\\ -\Delta (\delta v)={{\,\textrm{div}\,}}\big ((\delta A)\nabla v_\Omega \big )\quad \text {in }\Omega \ ,\qquad \delta v\in H^1_0(\Omega )\,. \end{aligned} \end{aligned}$$

Using the blow-up analysis from Sect. 4 and the argument from Lemma 8.2, we get

#### Lemma A.2

Let $\Omega$ be an optimal set for (A.2) and let $u:=u_\Omega$ and $v:=v_\Omega$ be the state functions from (A.1) and (A.4). Suppose that $x_0\in \partial \Omega \cap D$ and that $r_k\rightarrow 0$ are such that

$$\begin{aligned} \lim _{k\rightarrow \infty }u_{x_0,r_k}=u_0\qquad \text {and}\qquad \lim _{k\rightarrow \infty }v_{x_0,r_k}=v_0\, \end{aligned}$$

where $u_0,v_0$ are non-negative Lipschitz functions on $\mathbb {R}^d$ and both limits are locally uniform in $\mathbb {R}^d$, and where, as usual, we set:

$$\begin{aligned} u_{x_0,r_k}(x):=\frac{1}{r_k}u(x_0+r_kx)\qquad \text {and}\qquad v_{x_0,r_k}(x):=\frac{1}{r_k}v(x_0+r_kx). \end{aligned}$$

Then, $u_0$ and $v_0$ are proportional and there is a constant $\lambda >0$ such that $\lambda u_0$ is a global stable solution of the one-phase Bernoulli problem in the sense of Definition 7.3.

#### Proof

By the analysis of the blow-up sequences from Sect. 4, we get that $u_{x_0,r_k}$ and $v_{x_0,r_k}$ converge strongly in $H^1_{loc}(\mathbb {R}^d)$ respectively to $u_0$ and $v_0$, and also that the sets $\frac{1}{r_k}(\Omega -x_0)$ converge (in the local Hausdorff distance) to $\Omega _0=\{u_0>0\}=\{v_0>0\}$. Then, as in Lemma 8.2, for every smooth compactly supported vector field $\xi \in C^\infty _c(\mathbb {R}^d;\mathbb {R}^d)$, the first and the second variation of $\mathcal {F}$ pass to the limit, that is,

A.5 $$\begin{aligned} \begin{aligned}&\int _{\Omega _0} \Big (\nabla u_0\cdot (\delta A)\nabla v_0 + \Lambda {{\,\textrm{div}\,}}\xi \Big ) \, dx=0\,,\\&\int _{\Omega _0}\Big (\nabla u_0\cdot (\delta ^2 A)\nabla v_0-\nabla (\delta u_0)\cdot \nabla (\delta v_0)+\frac{\Lambda }{2}\big (({{\,\textrm{div}\,}}\xi )^2+\xi \cdot \nabla ({{\,\textrm{div}\,}}\,\xi )\big )\Big ) dx\geqq 0\,, \end{aligned} \end{aligned}$$

where $\delta A$ and $\delta ^2A$ are as in (2.6), and where $\delta u_0$ and $\delta v_0$ solve the PDE (see Sect. 7.1)

$$\begin{aligned} \begin{aligned} -\Delta (\delta u_0)={{\,\textrm{div}\,}}\big ((\delta A)\nabla u_0\big )\quad \text {in }\Omega _0\ ,\qquad \delta u_0\in \dot{H}^1_0(\Omega _0)\,;\\ -\Delta (\delta v_0)={{\,\textrm{div}\,}}\big ((\delta A)\nabla v_0\big )\quad \text {in }\Omega _0\ ,\qquad \delta v_0\in \dot{H}^1_0(\Omega _0)\,. \end{aligned} \end{aligned}$$

On the other hand, since by [1] the Boundary Harnack Principle holds on the optimal set $\Omega$, we get that the blow-up limits are proportional, that is, $v_0=Cu_0$ for some $C>0$. Finally, we notice that, up to multiplying $u_0$ by a constant, the inequalities (A.5) correspond precisely to the stability condition (7.8) in Definition 7.3, which concludes the proof of the lemma. $\square$

### A.3 Main Theorem

Let now $\Omega$ be an optimal set for (A.2). We define the regular and the singular parts of the free boundary $\partial \Omega \cap D$ as follows. The regular part, $\text { Reg}(\partial \Omega )$, is the set of points $x_0\in \partial \Omega \cap D$ at which there exists a blow up limit $u_0,v_0:\mathbb {R}^d\rightarrow \mathbb {R}$ of the form

$$\begin{aligned} u_0(x)=\alpha (x\cdot \nu )_+\quad \text {and}\quad v_0(x)=\beta (x\cdot \nu )_+, \end{aligned}$$

for some unit vector $\nu \in \mathbb {R}^d$ and some constants $\alpha >0$ and $\beta >0$ such that $\alpha \beta =\Lambda$. The remaining part of the free boundary is the singular set:

$$\begin{aligned} \text { Sing}(\partial \Omega ):=(\partial \Omega \cap D)\setminus \text { Reg}(\partial \Omega ). \end{aligned}$$

Then, in terms of the decomposition into $\partial \Omega \cap D=\text { Reg}(\partial \Omega )\cup \text { Sing}(\partial \Omega )$, we can rewrite the regularity theorem of Aguilera–Caffarelli–Spruck (for the penalized problem) as follows:

#### Theorem A.3

(Aguilera–Caffarelli–Spruck [1]) If $\Omega$ is a solution to (A.2) in a domain $D\subset \mathbb {R}^d$, then $\text { Reg}(\partial \Omega )$ is a relatively open subset of $\partial \Omega \cap D$ and (locally) a $C^\infty$ manifold.

For what concerns the singular set, by reasoning as in Theorem 8.1 and by applying Theorems 7.9 and 7.8, we obtain the following result, which in particular implies that in the physically relevant dimension $d=3$ (and also in $d=2$ and $d=4$) the free boundary $\partial \Omega \cap D$ of a solution $\Omega$ to (A.2) is $C^\infty$ smooth.

#### Theorem A.4

(Dimension of the singular set for solutions to (A.2)) If $\Omega$ is a solution to (A.2) in a domain $D\subset \mathbb {R}^d$, then the following holds:

(i) if $$d< d^*$$, then $$\text { Sing}(\partial \Omega )=\emptyset $$;
(ii) if $d\ge d^*$, then the Hausdorff dimension of $\text { Sing}(\partial \Omega )$ is at most $d-d^*$;

where $d^*$ is the critical dimension from Definition 7.7.

## Footnotes

1 Precisely, since $\mathcal G(u)=\infty$ as soon as *u* has positivity set of infinite measure, we will work with a localized version of $\mathcal G$, in which the integration is over compact sets.
